# Transcriptomic Analysis Using Olive Varieties and Breeding Progenies Identifies Candidate Genes Involved in Plant Architecture

**DOI:** 10.3389/fpls.2016.00240

**Published:** 2016-03-02

**Authors:** Juan J. González-Plaza, Inmaculada Ortiz-Martín, Antonio Muñoz-Mérida, Carmen García-López, José F. Sánchez-Sevilla, Francisco Luque, Oswaldo Trelles, Eduardo R. Bejarano, Raúl De La Rosa, Victoriano Valpuesta, Carmen R. Beuzón

**Affiliations:** ^1^Departamento de Biología Celular, Genética y Fisiología, Facultad de Ciencias, Instituto de Hortofruticultura Subtropical y Mediterránea, Universidad de Málaga - Consejo Superior de Investigaciones CientíficasMálaga, Spain; ^2^Departamento Arquitectura de Computadores, Escuela Técnica Superior de Ingeniería Informática, Universidad de MálagaMálaga, Spain; ^3^Center for Advanced Studies in Olive Grove and Olive Oils, University of JaénJaén, Spain; ^4^Centro IFAPA de ChurrianaMálaga, Spain; ^5^Centro IFAPA Alameda del ObispoCórdoba, Spain; ^6^Departamento de Biología Molecular y Bioquímica, Facultad de Ciencias, Instituto de Hortofruticultura Subtropical y Mediterránea, Universidad de Málaga - Consejo Superior de Investigaciones CientíficasMálaga, Spain

**Keywords:** plant architecture, *Olea europea*, RT-*q*PCR, dwarf phenotype, microarray, transcriptomics

## Abstract

Plant architecture is a critical trait in fruit crops that can significantly influence yield, pruning, planting density and harvesting. Little is known about how plant architecture is genetically determined in olive, were most of the existing varieties are traditional with an architecture poorly suited for modern growing and harvesting systems. In the present study, we have carried out microarray analysis of meristematic tissue to compare expression profiles of olive varieties displaying differences in architecture, as well as seedlings from their cross pooled on the basis of their sharing architecture-related phenotypes. The microarray used, previously developed by our group has already been applied to identify candidates genes involved in regulating juvenile to adult transition in the shoot apex of seedlings. Varieties with distinct architecture phenotypes and individuals from segregating progenies displaying opposite architecture features were used to link phenotype to expression. Here, we identify 2252 differentially expressed genes (DEGs) associated to differences in plant architecture. Microarray results were validated by quantitative RT-PCR carried out on genes with functional annotation likely related to plant architecture. Twelve of these genes were further analyzed in individual seedlings of the corresponding pool. We also examined *Arabidopsis* mutants in putative orthologs of these targeted candidate genes, finding altered architecture for most of them. This supports a functional conservation between species and potential biological relevance of the candidate genes identified. This study is the first to identify genes associated to plant architecture in olive, and the results obtained could be of great help in future programs aimed at selecting phenotypes adapted to modern cultivation practices in this species.

## Introduction

Plant architecture is a factor of paramount importance for agriculture, affecting the suitability of a plant for cultivation, yield, light assimilation, and harvesting (Reinhardt and Kuhlemeier, 2002; Hanan et al., 2003). A balance between endogenous growth processes and environmental limitations determines plant architecture (Barthélémy and Caraglio, 2007), however genotype is believed to be the main factor (Busov et al., 2008). A good example of the extreme importance of genetic determination of plant architecture for agriculture is the impact that variation at *teosinte branched 1* has had in the domestication of maize (Doebley et al., 1995). Plant architecture results from the branching pattern, size, shape, and position of leaves and flowers in the plant (Reinhardt and Kuhlemeier, 2002; McSteen and Leyser, 2005). Its complexity is defined by the ability to establish new axis of growth during post-embryonic development, through differentiation of axillary meristems. Axillary meristems can initiate growth after they are formed or remain dormant before developing (Costes et al., 2006). The regulation of shoot growth is also a factor that defines the vegetative branching pattern (McSteen and Leyser, 2005; Schmitz and Theres, 2005). Time and degree of shoot branching is determined by environmental or endogenous signals, being the hormones auxin, cytokinins, gibberellins or strigolactones, examples of the later type of signals (Gomez-Roldan et al., 2008; Umehara et al., 2008; Vogel et al., 2010). In *Arabidopsis*, the central molecular mechanism of growth regulation in the shoot apical meristem (SAM), is the WUS-CLV feedback loop (Turnbull, 2005; Wang and Li, 2008), in which the product of *WUS* promotes growth in the meristem, and it is itself repressed by the product of the *SYD* gen (Kwon et al., 2005). The activity of lateral meristems during reproductive development is key to the establishment of the different structures that lead to flower formation, and one of the most important elements for architecture and reproductive success (Schmitz and Theres, 2005).

The molecular determination of plant architecture has been studied mainly in annual crops as *Arabidopsis thaliana, Antirrhinum majus*, petunia (*Petunia hybrida*), pea (*Pisum sativum*), tomato (*Solanum lycopersicum*), maize (*Zea mays*) or rice (*Oryza sativa*), showing that plant height control is important as it directly influences yield (Wang and Li, 2006). Much less is known of the molecular mechanisms that regulate plant architecture in trees. Trees are perennial woody plants with a trunk or prominent primary shoot from which lateral branches emerge that have appeared repeatedly during natural evolution (Hollender and Dardick, 2015). Overall height, pattern and periodicity of branching, size, growth angle and orientation of each branch, are the main but not the only parameters that determine tree. Trees are known to adjust their structures in response to environmental stimuli, mainly to light, nutrient availability and crowding (Tomlinson, 1983), however, growth habit, which determines plant architecture, has been shown to be highly dependent on the genotype, which constrains these responses (Segura et al., 2009; Baldi et al., 2013). Hormones such as auxin, cytokinins and giberellins have also been shown to play an important role in determining plant architecture in trees, while the role of others such as strigolactones has been less clearly established than for herbaceous annual species (Hollender and Dardick, 2015). Also, although *Wuschel-related homeobOX* or *WOX* genes can be found in all plant species sequenced to date, their role in regulating the SAM has only been characterized in herbaceous species (Costanzo et al., 2014). Tree architecture is also critical in fruit orchards to determine the suitability for a given growing system, plant density or mechanical harvesting (Costes et al., 2006; Badenes and Byrne, 2011). As an example, columnar growth habit is potentially beneficial for apple growers since they would allow higher density planting and require less pruning than standard tree types, however, since none of the columnar varieties available to date can compete with commercially successful varieties in terms of fruit quality and disease resistance, breeding for columnar growth habit in commercially competitive apple varieties would be of great interest (Looney and Lane, 1984; Tobutt, 1985; Lauri and Lespinnasse, 1993; Meulenbroek et al., 1998; Moriya et al., 2009, 2012; Petersen and Krost, 2013).

Olive (*Olea europaea* L.) is an economically relevant crop, since olive oil is one of the most important vegetable oils in the world (Conde et al., 2008). However, most of the existing varieties are traditional (Haouane et al., 2011; Belaj et al., 2012), and not well adapted to new trends in olive growing (Barranco et al., 2010). These trends include increases from the traditional 100 trees per ha to intensive plantations of 400 or even 2000 trees/ha, in hedgerow growing systems (Villalobos et al., 2006; Baptista and Biswas, 2010). Adapting canopy size and shape to high planting densities is currently achieved by pruning, aimed to reach the highest leaf/wood ratio (García-Ortiz et al., 2004; Rosati et al., 2013), while reducing shading (Boardman, 1977; Gregoriou et al., 2007). Such practices are applied for example to plantations of Arbequina (Tous and Romero, 1993; Barranco et al., 2005), a Spanish variety widely used in intensive and hedgerow orchards due to its medium to low vigor and good agronomic behavior (Rallo et al., 2008; Larbi et al., 2011; Rosati et al., 2013). However, even varieties of this vigor show early competition for radiation in high-density orchards (Rallo et al., 2008; Connor et al., 2009), which can result in significant yield losses if adequate pruning practices are not applied (García-Ortiz et al., 2004; Pastor et al., 2005). To date, breeders have obtained two varieties with a tree architecture specifically adapted to high planting densities, Askal in Israel (Lavee et al., 2003) and Chiquitita or Sikitita (hereafter Chiquitita) in Spain (Rallo et al., 2008). Such bred varieties can potentially maximize the usage of light, and would thus facilitate maintenance reducing the need for pruning. However, despite the interest in breeding for specific tree architecture in olive, only few works have attempted to analyze its genetic basis, either through heredability studies (Hammami et al., 2011, 2012; Ben Sadok et al., 2013) or QTL analysis (Sadok et al., 2013), and none of them have identified genes associated to growth habit.

Genomics and associated tools are enabling researchers to tackle questions regarding the molecular and genetic mechanisms underlying determination of growth habit in historically intractable organisms for genetic analysis such as trees (Hollender and Dardick, 2015). Although the analysis of quantitative trait loci (QTL) has been very useful for breeding, they are often species-specific or even cultivar-specific. Thus, the identification and functional characterization of genes that contribute to specific aspects of tree architecture is critical to fully exploit tree genomes for crop improvement, enabling both conventional breeding and biotechnological improvements, as well as providing fundamental knowledge about tree development. The identification of candidate genes for complex traits like plant architecture can be greatly helped by the use of high-throughput analysis, such as microarrays analysis, often applied to this purpose in non-sequenced species (Alba et al., 2004; Utsumi et al., 2012). Microarray analysis have been successfully applied to the identification of candidate genes in tree species, such as *Populus trichocarpa* (Cohen et al., 2010; Di Baccio et al., 2011), or more recently to grapevine (*Vitis vinifera*) (Díaz-Riquelme et al., 2012) and olive (García-López et al., 2014). They have also been used to discover the genetic determinants of columnar growth habit in apple (Krost et al., 2012; Petersen and Krost, 2013). Here, we use a transcriptomic approach for the identification of candidate genes involved in plant architecture in olive. We use an olive microarray developed by the OLEAGEN Consortium (Muñoz-Mérida et al., 2013), and already applied to the analysis of juvenile to adult transition in this species (García-López et al., 2014). In the framework of an olive-breeding program carried out by the University of Córdoba and IFAPA, we analyze RNA from varieties with distinct architecture phenotypes, as well as groups of seedlings with a common genetic background, pooled by their architectural features. One of the varieties selected was Chiquitita, bred within this program and displaying a very peculiar shrubby growth habit with a very compact yet weeping canopy, very suitable for high-density orchards and harvesting with straddle machines (Rallo et al., 2008). Trees with non-standard growth habits can provide an excellent starting point for understanding the complex developmental processes that determine tree architecture (Hollender and Dardick, 2015). Transcriptomic analysis of the microarray data generated, allowed us to identify 2252 differentially expressed genes (DEGs) as potentially involved in determining plant architecture in olive. Quantitative RT-PCR assays on selected varieties and individual seedlings were additionally used to confirm microarray results, and to further link phenotype to expression. Functional proof was sought for through phenotypic analysis of *Arabidopsis* mutants in putative orthologs of selected candidate genes. The altered architecture phenotypes found for most of them support the conservation of their potential association to plant architecture and their possible future interest for olive breeding.

## Materials and methods

### Plant material and phenotyping

**Figure 1:**
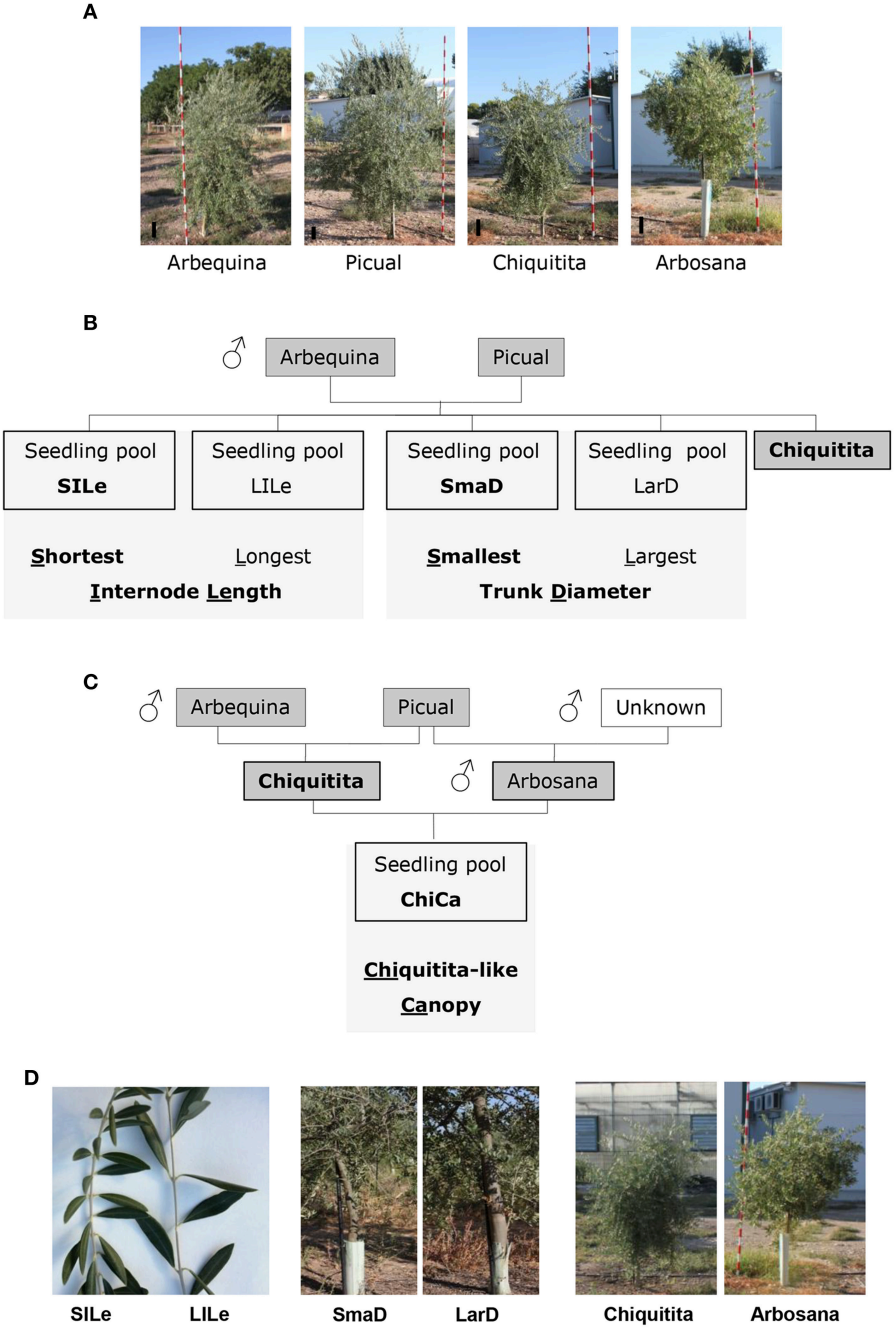
**Varieties and seedling pools used in this study. (A)** Pictures of 4-year old trees representative for the varieties Arbequina, Picual, Chiquitita, and Arbosana. A reference for the scale is included as a black line in all images; **(B,C)** Genetic relationships between varieties (shaded in dark gray), architectural traits (shaded in light gray) and phenotypes (boxed) for seedling pools selected from the Picual × Arbequina **(B)** and the Chiquitita × Arbequina **(C)** crosses. The phenotypes for each trait potentially best suited for high density planting are shown in bold. **(D)** Phenotypes of the seedling pools used in the microarray analysis: left panel shows images of typical phenotypes of individual seedlings selected to form the short internode length (SILe) and long internode length (LILe), selected for the internode length-based comparisons; center panel shows typical phenotypes of individual seedlings selected to form the small trunk diameter (SmaD) and large trunk diameter (LarD) selected for the diameter size-based comparisons; right panel shows images of the phenotypes of the varieties (Chiquitita and Arbosana) used for the growth habit-based comparisons.

Olive material was provided by the olive breeding program of Cordoba (De La Rosa et al., 2013). We carried out our study on olive varieties used as parents in olive breeding programs of Córdoba (De La Rosa et al., 2013), and seedlings from their crosses displaying distinct architectural phenotypes. One of the selected varieties, Chiquitita, displays a particular shrubby growth habit with a compact yet weeping canopy and was obtained in this breeding program from a Picual × Arbequina cross, which were also included in our dataset (Figure 1 and Table 1) (Rallo et al., 2008). Among a hundred and twenty seedlings, obtained from a Picual × Arbequina cross (León et al., 2004; Pérez et al., 2014), and previously phenotyped for architecture-related traits (Atienza et al., 2014) and data not shown), we selected those individuals that displayed the lowest and highest values for internode length or trunk diameter, traits that show a direct correlation with size and shape of the olive canopy (Hammami et al., 2011, 2012; Tables 1, 2). The length of four internodes was measured in five representative 1-year old branches for each seedling (Hammami et al., 2011). Diameter was measured in the main seedling trunk at 1 m height, as previously reported (Hammami et al., 2011). We completed the analysis with the seedling pool including those seedlings from a Chiquitita × Arbosana cross previously evaluated as displaying a Chiquitita-like growth habit and categorized as Ideotype 3 in a previous study (Hammami et al., 2011, 2012; Tables 1, 2). The shape of the canopy is the most complex of the traits analyzed, and as expected from a multi-loci associated trait, shows a continuous distribution in the Chiquitita × Arbosana progeny. Thus, only clear Chiquitita-like shrubby canopies, undistinguishable from that of the parent Chiquitita, and clearly distinguishable from its other parent Arbosana, were selected for the pool.

**Table 1:** **Varieties used in this study**.

**Sample**	**Tissue**	**Description**	**Use**
Chiquitita	Active meristems	Chiquitita (Picual (♀) × Arbequina (♂))	Microarray hybridization, RT-qPCRs
Picual	Active meristems	Picual	Microarray hybridization
Arbequina	Active meristems	Arbequina	Microarray hybridization
Arbosana	Active meristems	Arbosana (Arbosana (♀) × unknown (♂))	Microarray hybridization, RT-qPCRs
77.3	Active meristems	Chiquitita (♀) × Arbosana (♂)	RT-qPCRs
79.5	Active meristems	Chiquitita (♀) × Arbosana (♂)	RT-qPCRs
81.4	Active meristems	Chiquitita (♀) × Arbosana (♂)	RT-qPCRs
83.7	Active meristems	Chiquitita (♀) × Arbosana (♂)	RT-qPCRs
85.2	Active meristems	Chiquitita (♀) × Arbosana (♂)	RT-qPCRs
SILe pool	Active meristems	Picual (♀) × Arbequina (♂)	Microarray hybridization
LILe pool	Active meristems	Picual (♀) × Arbequina (♂)	Microarray hybridization
ChiCa pool	Active meristems	Chiquitita (♀) × Arbosana (♂)	Microarray hybridization
LarD pool	Active meristems	Picual (♀) × Arbequina (♂)	Microarray hybridization
SmaD pool	Active meristems	Picual (♀) × Arbequina (♂)	Microarray hybridization

*Pool samples contain several seedlings. SILe, short internodes length; LILe, long internodes length; ChiCa, Chiquitita-like canopy; LarD, large trunk diameter; SmaD, small trunk diameter*.

**Table 2:** **Phenotypic data for trunk diameter and internode length for the individual seedlings selected for the seedling pools**.

**Seedling**	**Mother**	**Pollen donor**	**Diameter (mm)**	**Mean internode length of branch 1 (mm)**	**Mean internode length of branch 2 (mm)**	**Mean internode length of branch 3 (mm)**	**Mean internode length of branch 4 (mm)**	**Mean internode length of branch 5 (mm)**	**Mean internode length (mm)**	**Seedling pool**
52	Picual	Arbequina	42.12	3.28	3.20	2.93	2.13	1.95	2.70	SmaD
44	Picual	Arbequina	53.81	2.50	4.15	3.08	2.75	2.13	2.92	SmaD
49	Picual	Arbequina	53.48	2.75	2.33	1.95	2.35	2.43	2.36	SmaD
52	Picual	Arbequina	53.49	1.35	2.33	1.90	2.38	2.13	2.02	SmaD
38	Picual	Arbequina	48.00	1.68	0.90	1.05	0.63	1.00	1.05	SmaD
40	Picual	Arbequina	50.68	0.85	0.88	0.83	0.80	0.85	0.84	SmaD
39	Picual	Arbequina	106.52	1.15	1.63	0.70	1.18	1.10	1.15	LarD
44	Picual	Arbequina	105.25	0.88	1.65	1.93	1.45	1.80	1.54	LarD
59	Picual	Arbequina	110.83	1.85	1.68	1.10	1.50	1.50	1.53	LarD
50	Picual	Arbequina	92.81	1.43	1.38	1.50	0.95	1.15	1.28	LarD
59	Picual	Arbequina	101.92	1.63	1.53	1.80	2.05	1.68	1.74	LarD
45	Picual	Arbequina	59.83	1.73	2.13	1.18	1.63	1.05	1.54	SILe
60	Picual	Arbequina	77.72	1.20	1.63	1.10	1.35	1.50	1.36	SILe
46	Picual	Arbequina	87.76	0.88	1.50	2.03	1.80	1.98	1.64	SILe
42	Picual	Arbequina	42.24	1.95	1.75	1.13	1.38	1.15	1.47	SILe
47	Picual	Arbequina	66.02	1.55	1.25	0.88	1.58	0.73	1.20	SILe
49	Picual	Arbequina	90.15	1.55	1.25	1.33	1.83	1.35	1.46	SILe
50	Picual	Arbequina	85.91	2.80	2.43	2.15	2.00	1.55	2.19	LILe
47	Picual	Arbequina	92.45	2.30	3.13	2.73	2.38	1.73	2.45	LILe
53	Picual	Arbequina	86.37	2.58	2.48	2.73	2.38	2.10	2.45	LILe
59	Picual	Arbequina	108.65	2.53	1.80	1.95	1.85	1.83	1.99	LILe
62	Picual	Arbequina	119.19	2.43	2.93	2.03	2.00	2.90	2.46	LILe
3	Chiquitita	Arbosana	57.67	2.15	2.35	2.35	2.08	2.60	2.31	ChiCa
5	Chiquitita	Arbosana	72.23	2.13	1.93	2.48	2.18	2.50	2.24	ChiCa
4	Chiquitita	Arbosana	75.71	2.15	2.15	2.50	1.70	1.75	2.05	ChiCa
7	Chiquitita	Arbosana	67.78	2.00	2.20	2.13	2.00	2.03	2.07	ChiCa
2	Chiquitita	Arbosana	60.38	1.75	1.80	1.55	2.50	2.40	2.00	ChiCa

### RNA processing and microarray analysis

Transcriptomic analysis were carried out using actively growing shoots, key in determining plant architecture (Reinhardt and Kuhlemeier, 2002; Schmitz and Theres, 2005), and successfully analyzed for this purpose in apple (Krost et al., 2012). Harvesting was carried out at the end of Spring. Samples were immediately frozen in liquid nitrogen, and maintained at −80°C. Actively growing shoots were selected and approximately 1–2-cms from its tip (including the SAM) harvested for processing. Samples were composed of active shoots harvested from a single individual, which was processed individually or in pools depending on the type of sample and/or experiment. Four to six seedlings were used to generate pools, and were harvested individually and 0.2 g of tissue per individual mixed and processed prior to RNA extraction. The number of individuals displaying each of the selected phenotypes within the segregating progenies available determined the exact number of seedlings used for each pool.

RNA was isolated from olive meristems using a previously reported protocol (Bilgin et al., 2009). Shoots were harvested into three separate samples (biological replicates). One g of frozen tissue was used for RNA extraction in olive varieties. For RNA extraction of seedling pools, 0.2 g of each individual was used to complete the 1 g of tissue. *Arabidopsis* RNA was extracted using Trisure™ (Bioline, USA) following the vendor methodology. All samples were treated with RNase-free DNAse I (Takara Bio Inc., Japan), following the instructions provided. RNA quality was tested by electrophoresis and Nanodrop spectrophotometer (Thermo Fisher Scientific, USA), which was also used to determine concentration. RNA extractions were tested by PCR to ensure no trace of contaminating genomic DNA was detectable. Genomic DNA, used as a PCR positive control, was extracted using the Jet Flex Extraction kit (Genomed, Germany).

Olive cDNA was synthesized using Transcriptor First Strand cDNA Synthesis kit (Roche, Germany) with random hexanucleotides (Promega, USA). cDNAs were diluted 1:5 in molecular grade biology water and used as template for RT-*q*PCR. *Arabidopsis* cDNA synthesis was carried out using SuperscriptII reverse transcriptase (Life Technologies, USA), and iScript (BioRad, USA). Technical triplicates were obtained using the same cDNA preparation. Three biological replicates were hybridized against the microarray for each genotype or pool. Expression profiles for each of the replicates showed a high correlation in each of the 9 samples used to hybridize the array (Table S3).

We used a previously reported 60-mer oligonucleotide microarray (García-López et al., 2014), designed by Roche Nimblegen Inc. (Madison, USA), including 37449 unigenes assembled from part of the sequences obtained by Muñoz-Mérida et al. (2013). Labeling, hybridization, and scanning were performed following Nimblegen procedures. The results obtained were normalized to eliminate systematic variation not caused by biological effects (Bremer and Doerge, 2010). The correlation between biological replicates was tested following a previously described method (Zahn et al., 2010). Expression data accessible through GEO Series accession number GSE60284 at NCBI Gene Expression Omnibus (Edgar et al., 2002). Expression profiles were compared following an approach previously applied to *Populus* (Street et al., 2006) and *Eschscholzia californica* (Zahn et al., 2010) using an interwoven loop design and pooled individuals from the same cross, sharing the relevant phenotype. In the three-way comparisons for quantitative traits, genes selected displayed significant differences in expression (more than a 2-fold, α < 0.05, Student's *T*-test) between the pools of individuals displaying opposite phenotypes (Small trunk diameter or SmaD vs. large trunk diameter or LarD, and short internode length SILe vs. long internode length LILe), but their expression was not significantly different between Chiquitita and the pool with the similar phenotype (SmaD or SILe, respectively). For the three-way comparisons carried out for growth habit, the genes selected were those showing significant differences in expression between Chiquitita and Arbosana, but not between Chiquitita and the Chiquitita-like canopy (ChiCa) pool.

### Functional annotation

Unigenes in the microarray were functionally annotated using Sma3s (Muñoz-Mérida et al., 2014), and compared with the published transcriptome (Muñoz-Mérida et al., 2013). The annotation generated was loaded as a tab-separated file in the Blast2GO 3.0 suite (Conesa et al., 2005), to retrieve the GO structure of our data. A directed acyclic graph (DAG) (Conesa et al., 2005) was made with default variables. Statistical analysis of GO terms enrichment was carried out using the Blast2GO suite (Conesa et al., 2005) to perform a two-tailed Fisher's exact test (Bremer and Doerge, 2010), a non-parametric test for independence that calculates the false discovery rate (FDR) (Benjamini and Hochberg, 1995) (term filter value of 0.05). For MapMan (Thimm et al., 2004) suite, a functional annotation of the AS8trim3 assembly (Muñoz-Mérida et al., 2013) was performed using the Mercator web tool (Crowhurst et al., 2008). Putative orthologs for olive candidate genes in *Arabidopsis* were identified using BlastX analysis and an *e*-value limit of 3 × 10^−8^, adjusted to a Rost curve (Rost, 1999).

### Real-time PCR

RNA and cDNA samples for real-time PCR analysis were obtained as described above for microarray analysis. SYBR Green RT-PCR reactions of olive samples were performed using Sofast Evagreen Supermix (BioRad, USA) in 10 μl volumes containing 1 μl of cDNA template and 10 μM forward and reverse primers (Table S1). The program used was: 30 s at 98°C; 40 cycles of 98°C (5 s), 60°C (10 s), 72 (3 s), and 78°C (3 s); with a melting curve from 65°C to 95°C (increment 0.5°C/s). Reactions were run in BioRad CFX96 (BioRad, USA) and analyzed by MyIQ™ software. Threshold detection parameters were adjusted automatically. Expression of triplicate samples of three independent experiments for each transcript was calculated using the ΔCt method (Real-Time PCR Applications Guide, 2006, BioRad, USA). Technical triplicates were obtained using the same cDNA preparation. An internal control of constitutive olive actin was used for the normalization of results. The constitutive normalization control was previously selected as the most constant in expression following comparison of several genes and primer pairs on different olive tissues (García-López et al., 2014).

### Genotyping and phenotyping *Arabidopsis* mutants

Mutants of *Arabidopsis* were ordered from Nottingham *Arabidopsis* Stock Centre (NASC) (Scholl et al., 2000; Table S1). Mutants *svp*-41 and *dwf* 4-101/*snp*2-1, were kindly donated by Dr. Peter Huijser (Department of Molecular Plant Genetics, Max Planck Institute for Plant Breeding Research, Cologne, Germany), and by Dr. Kotaro T. Yamamoto (Division of Biological Sciences, Graduate School of Science and Graduate School of Environmental Earth Science, University of Hokkaido, Sapporo, Japan), respectively. Plants were grown in chambers with 16 h light: 8 h dark cycles at 21°C.

Mutants were genotyped to determine homozygosis (Table S1) prior to evaluation. Oligonucleotides for genotyping *Arabidopsis* mutants (Alonso et al., 2003) were designed using the tool provided by the Salk Institute Genomic Analysis Laboratory (http://signal.salk.edu/tdnaprimers.2.html) (Table S1). Genomic DNA was extracted using a modification of a previously published method (Edwards et al., 1991). Briefly, 400 μL of extraction buffer (200 mM Tris-Base, C_4_H_11_NO_3_, HCl adjusted pH 7.5; 250 mM NaCl; 25 mM EDTA, C_10_H_14_N_2_Na_2_O${}_{8}^{*}$2H_2_O; 0.5% V/V SDS, C_12_H_25_NaO_4_S) were placed in a 1.5 mL microcentrifuge tube with three metal beads. A segment of leaf, cut using the lid of the tube, was disaggregated in Tissuelyser II (Qiagen, Germany) using two pulses of 30 s each. Supernatant was transferred and centrifuged at 20,200 g for 5 min. Three hundred micro liter of supernatant was mixed with 300 μL 2-propanol and centrifuged at 20,200 g for 5 min. Supernatant was discarded and pellet washed with 500 μL 70% V/V ethanol, and centrifuged for 2 min at 20,200 g. the resulting pellet was dried and resuspended in 50 μL Tris pH8.

Amplification was performed with Techne® Endurance TC-412 96-well thermocycler, using GoTaq® Flexi DNA Polymerase (Promega; Madison, WI, USA) and the program: 95°C5 min; 32 cycles 30 s at 94°C, 30 s at 55°C, and 1 min at 72°C; followed by 7 min at 72°C. Mutants *svp41* have a restriction site recognized by NlaIV (New England Biolabs Inc., USA), absent in wild plants. The restriction reaction was performed according to the vendor indications.

Phenotypic characterization of the genotyped mutants was carried out using a modification of a previously published method (Boyes et al., 2001) detailed in Table S2 and included analysis of mRNA accumulation.

### Statistical analysis

A Student's *T*-test (2 tails, type 2) was performed to establish genes displaying differential expression whenever the following statements were satisfied: α < 0.05 and 2 $<\bar{x}∕ȳ>$ 0.5, where *x*∕ȳ represents the Fold Change. A Pearson's correlation test (Zahn et al., 2010) was performed between biological replicates. We used a Kruskal-Wallis test to establish significant differences for median expression values of candidate genes sets. Tests were carried out using Sigmastat Centurion XVI. Multiexperiment Viewer (Saeed et al., 2003) was used to obtain hierarchical grouping of our samples according to their expression profiles. In order to contrast the significance of the lists of DEG obtained, random sets of genes with the same number of elements that the selected ones were generated using Excel, they were used as negative controls for statistical purposes.

## Results

### Comparative transcriptomic analysis

Transcriptomic analysis included Chiquitita, obtained within the breeding program of the University of Cordoba and displaying a very peculiar, non-standard, shrubby growth habit with a very compact yet weeping canopy (Figure 1). Chiquitita descends from a Picual × Arbequina, which generates a highly segregating progeny for plant architecture-related phenotypes among others (Figure 1). We generated pools composed of individual seedlings among 120 available from a Picual × Arbequina cross. These individual seedlings were selected on the basis of their displaying maximal differences in internode length or trunk diameter (Tables 1, 2). The growth habit of Chiquitita is markedly distinct from that of its parent Picual, the most cultivated variety in Spain, with high vigor and a spread out canopy, also used in this study. The shrubby canopy displayed by Chiquitita is also distinct from its other parent, Arbequina, and its half sibling Arbosana, which complete our reference set (Table 1). Although more similar in vigor to Chiquitita than Picual, Arbequina, and Arbosana have distinct canopies and reach notably higher heights in older trees, requiring considerable pruning in older trees when grown in high density orchards. Individuals with the distinct shrubby canopy displayed by Chiquitita are obtained from a Chiquitita × Arbosana cross. Such individuals were also used to generate a seedling pool, the Chiquitita-like canopy pool (ChiCa) (Tables 1, 2; Figure 1).

**Figure 2:**
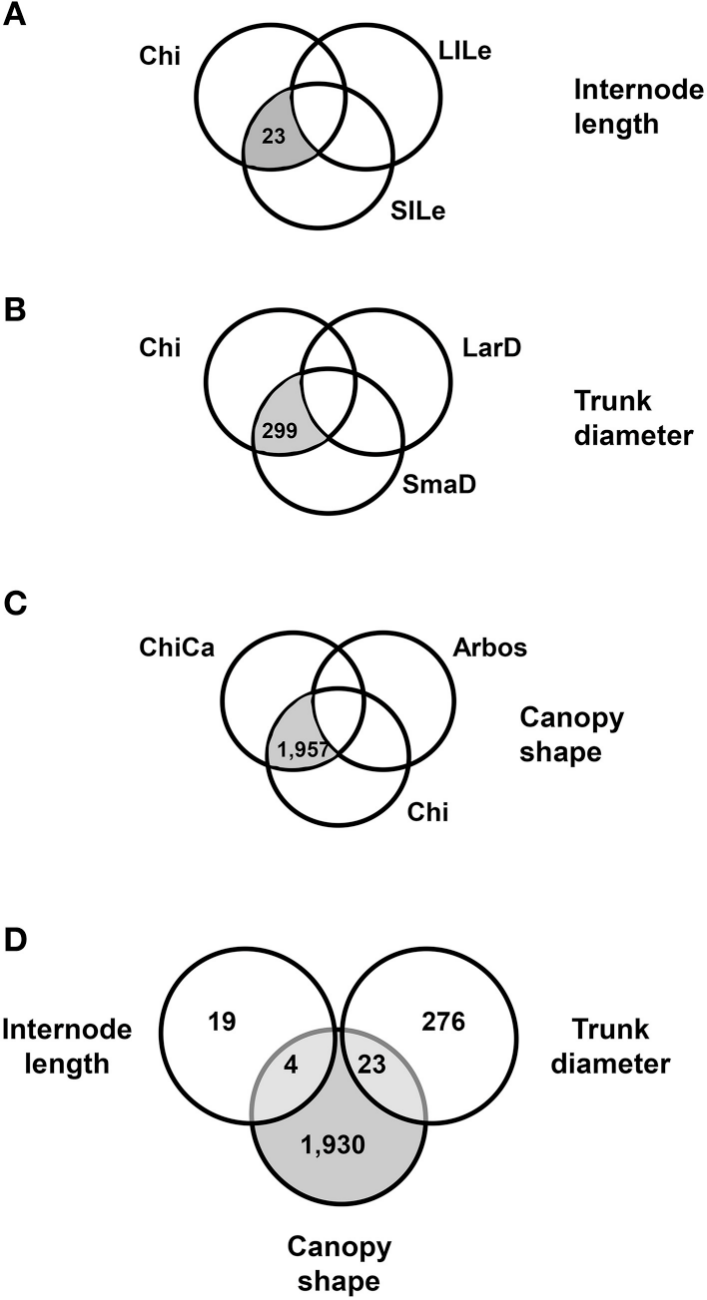
**Venn diagrams showing the number of candidate genes identified through phenotype-based three-way comparisons carried out among samples obtained from active meristems from genetically related genotypes. (A)** Comparisons carried out on the basis of internode length: selected candidate genes (shaded in gray) displayed similar expression (differences were not statistically significant) in Chiquitita (Chi) and the seedling pool corresponding to small internode length (SILe), but significantly different expression in the seedling pool corresponding to long internode length (LILe). **(B)** Comparisons carried out on the basis of trunk diameter: selected candidate genes (shaded in gray) displayed similar expression (differences were not statistically significant) in Chiquitita (Chi) and the seedling pool corresponding to small trunk diameter (SmaD), but significantly different expression in the seedling pool corresponding to large trunk diameter (LarD). **(C)** Comparisons carried out on the bases of canopy shape: selected candidate genes (shaded in gray) displayed similar expression (differences were not statistically significant) in Chiquitita (Chi) and the seedling pool corresponding to Chiquitita-like canopy (ChiCa), but significantly different expression in Arbosana (Arbos). **(D)** Venn diagram showing the overlap between candidate-gene sets obtained through the three-way comparisons.

We compared the expression profiles for the pools of seedlings displaying opposite phenotypes for two quantitative traits, internode length and trunk diameter (Figure 1D). We found 201 genes differentially expressed (DEGs) between the short (SILe) and long (LILe) internode length pools (Figure 2A), and 896 DEGs between the small (SmaD) and large (LarD) trunk diameter pools (Figure 2B). To further refine our search for DEGs associated to plant architecture, we carried out three-way comparisons, based on phenotype and genetic relationship, introducing Chiquitita in each comparison, as it displays the phenotype of interest for each trait and has a close genetic relationship with the samples analyzed (Figure 1). The three-way comparisons were designed to select among the DEGs between pools with opposite phenotypes, those with similar expression in Chiquitita and the pools displaying Chiquitita-like phenotypes (short internode length or SILe, and small trunk diameter or SmaD). Following this design, 23 out of the 201 DEGs between the short (SILe) and long (LiLe) internode length pools (Figure 2A), and 299 out of the 896 DEGS between the small (SmaD) and large (LarD) trunk diameter pools (Figure 2B), displayed similar expression in Chiquitita and their siblings from the short internode pool (SILe) and the small trunk diameter pool (SmaD), respectively.

As a further statistical validation of the selected DEGs, differences between the median expression values for the 299 genes selected for being statistically significant between SmaD and LarD were still statistically significant (*P* = 0) when all 9 samples generated in the study were considered, whereas those of a randomly selected 299-genes group were not (*P* = 0.9999). Differences between the median expression values of the candidates genes selected for internode length, were however not significant across all samples of the study, probably owing to the small size of this group.

We completed the analysis with the seedling pool including those seedlings from a Chiquitita × Arbosana cross displaying a Chiquitita-like growth habit (ChiCa). Almost two thousand genes (1957) displayed significant differences between their expression in Chiquitita or in its descendants forming the ChiCa pool, and their expression in Arbosana (Figure 2C). The differences between the median expression values for these 1957 candidate genes were also significant (*P* = 7.37 × 10^−9^) across all 9 samples generated in the study, whereas those displayed by a randomly selected 1957-genes group were not (*P* = 0.9652).

Four of the 23 candidate genes selected in the internodes length, and 23 of the 299 candidates selected in the trunk diameter three-way comparisons, were also identified as part of the 1957 candidates for canopy shape (Figure 2D). None of the candidate genes was identified in all three analyses, probably due to the specificity of some of the traits involved. The degree of overlap between these analyses was significantly higher than expected by chance, as determined through the analysis of the Poisson distributions generated (*P* = 0.0078; *P* = 0.029; respectively). These results further support the significance of our findings, and serve as statistical validation of the results obtained in the analysis of internodes length, the smaller candidate genes set. Therefore, the total number of genes identified through this analysis as potentially involved in the determination of plant architecture was 2252 (Table S4).

### Functional annotation of the candidate genes

**Figure 3:**
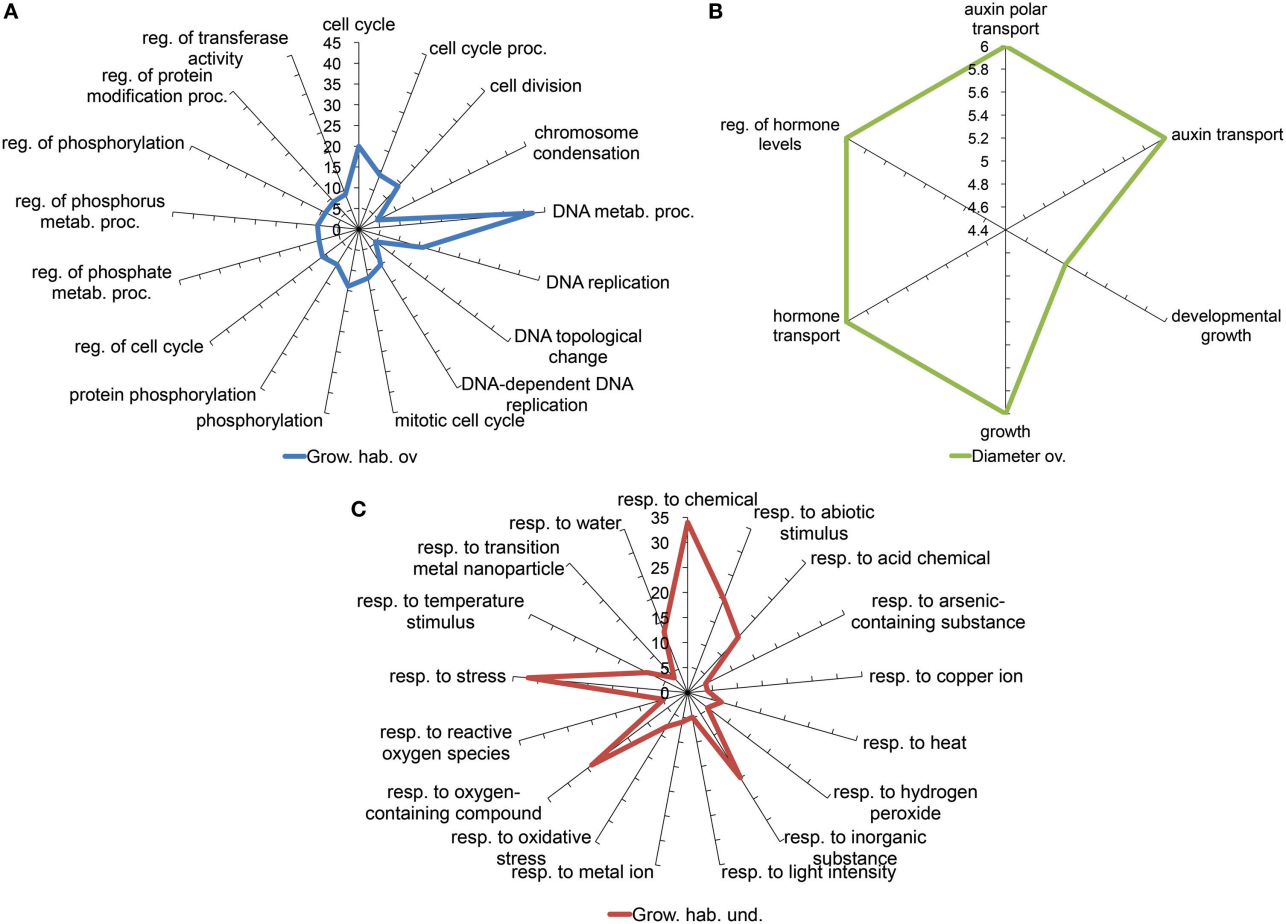
**Spider plots representing GO terms over-represented in the list of DEGs obtained from the (A) growth habit, or (B) trunk diameter comparisons, overexpressed in association to the desired phenotypes (overexpressed in Chiquitita or ChiCa vs. Arbosana, and SmaD vs. LarD, respectively); and over-represented in the lists of DEGS obtained from the growth habit comparisons under-expressed in association to the desired phenotype (C)**. Over-represented GO terms were identified through a Fisher's Exact Test performed comparing terms associated to each DEGs list and those associated to the whole array. The axis display the number of DEGs for each functional term. Grow. hab. ov., Growth habit overexpressed; Diameter ov., Diameter overexpressed; Grow. hab. und., Growth habit underexpressed; reg., regulation; metab., metabolic; proc., process; resp., response.

**Figure 4:**
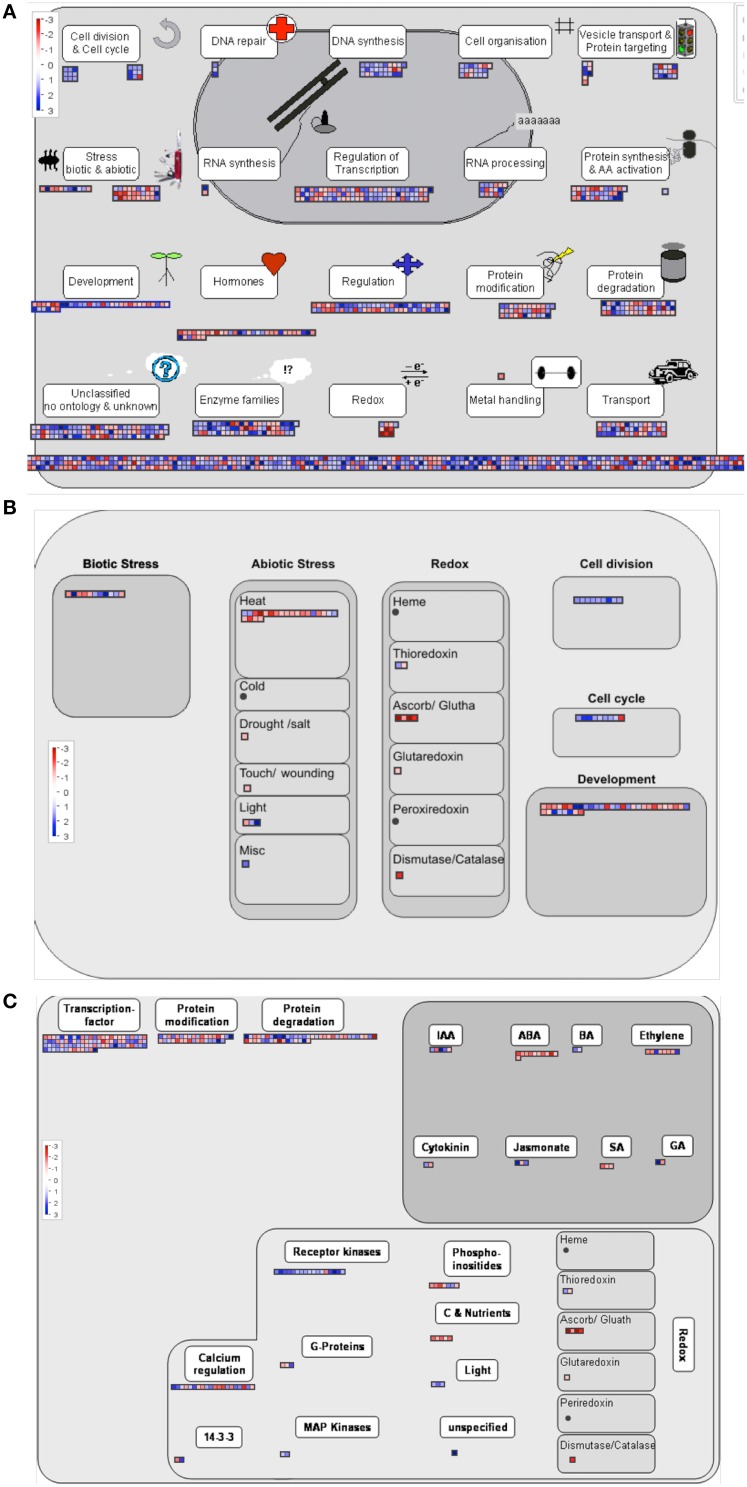
**MapMan visualization of functional annotation of the candidate genes**. The Chiquitita/Arbosana expression ratio for genes with a functional annotation among the 1957 candidates selected from the growth habit comparisons are shown to be associated to different processes: **(A)** Cell responses overview, **(B)** Cell functions overview, and **(C)** Regulation overview. Color scale represents: higher expression in Chiquitita than Arbosana (blue), or lower expression in Chiquitita than in Arbosana (red).

Two approaches were followed to get an overview of the type of processes represented in our selected candidate gene set. Both are based on the use of gene ontology annotations or GO terms (Yon Rhee et al., 2008). A Fisher test was performed in order to determine which GO terms are significantly over or underrepresented among the candidate genes in comparison to the complete microarray. Since the relative sizes of the lists generated in the different comparisons are very different, to avoid biasing the assay toward the type of processes identified in the larger candidate set, we analyzed the individual lists obtained from the internode length, trunk diameter and growth habit comparisons, separately. We further split the lists into those overexpressed or underexpressed in the samples corresponding to the Chiquitita-like phenotypes phenotypes, for a more detailed description. Figure 3 shows the GO terms related to biological processes over represented in the list of genes either overexpressed (1111) (Figure 3A) or underexpressed (846) (Figure 3B) in Chiquita vs. Arbosana corresponding to the 1957 DEGs selected in the growth habit comparisons, and the 235 overexpressed DEGs from the list of 299-candidate DEGs selected in the diameter size comparisons (Figure 3C). In the later case, only genes overexpressed in the SmaD/LarD ratio were shown since no GO term displayed significant enrichment in the list of those underexpressed. Similarly, no GO terms displayed significant enrichment in the list of genes selected from the internode length comparisons, probably due to the small size of these gene sets (Beissbarth, 2006). The most represented terms among those genes obtained from the growth habit comparison that are overexpressed in Chiquitita relate to processes associated to regulation of meristematic activity, such as regulation of cell cycle, DNA replication, and chromosome condensation (Figure 3A). Those terms enriched in the genes obtained from the same comparison but underexpressed in Chiquitita were majorly associated to responses to abiotic signals and hormone regulation (Figure 3B). Finally the set of candidate genes obtained in the trunk diameter comparisons and overexpressed in seedlings with small diameter was enriched in growth related terms, such as developmental growth or auxin transport (Figure 3C). Visualization by MapMan, used as a complementary means to describe the functions associated to our selected gene candidates, mostly supported these results. Although many of the DEGs grouped in the unknown unclassified category, a considerable number of genes were found in association to cell functions in keeping with the results obtained using Fisher's exact test, such as transcriptional regulation, regulation, protein synthesis, modification and degradation, development, response to hormones and response to biotic and abiotic stresses, DNA synthesis and cell division, as well as more general categories such as enzymes or transport (Figure 4A). This is particularly clear when an overview of the cellular functions associated to these DEGs was represented (Figure 4B). Interestingly, we found a considerable number of genes associated to response to biotic stress, which could result from cross annotation with those associated to abiotic stresses. We also found a considerable number of annotations for transcription factors (Figure 4C). MapMan analysis associated most of these annotations to MYB (Martin and Paz-Ares, 1997), and WRKY factors (Rushton et al., 2010). In addition, this analysis found a number of genes annotated as associated to ubiquitin-mediated protein degradation, in particular to F-Box-like proteins (Figure 4C and data not shown).

### Real time PCR expression analyses

**Figure 5:**
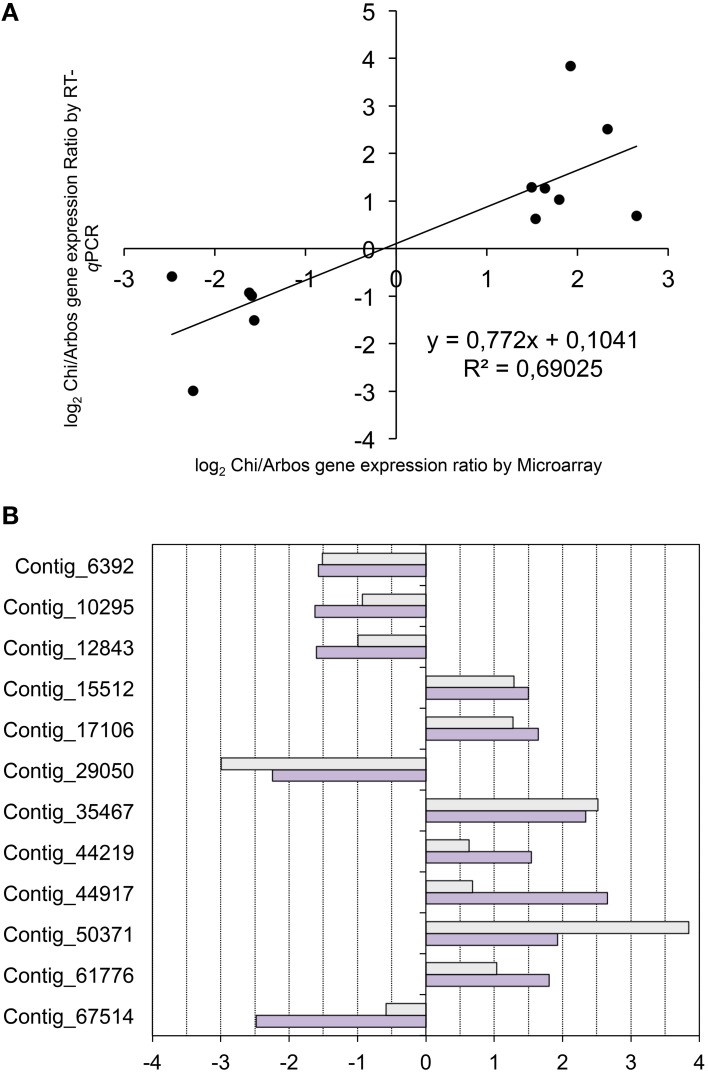
**(A)** Correlation analysis between expression values (log2 Chi/Arbos) obtained from the RT-*q*PCR assays vs. those obtained from the microarray analysis. Correlation values and the corresponding linear equation are represented in the chart. **(B)** Histogram representing the comparison between log2 Chi/Arbos for the 12 candidate genes selected obtained using either RT-*q*PCR (light gray) or microarray (dark gray).

The candidate gene list was queried and subjected to filtering and manual curation in order to select 12 genes for expression analyses. The following criteria was followed to select these genes: (*i*) elevated expression values and significant fold change differences; (*ii*) functional annotation compatible with expected functions (e.g., transcriptional factors), and (*iii*) potential functional conservation estimated by comparing with the *Arabidopsis* database (The Uniprot, 2008). Since the genes selected were identified as part of the subset of candidate genes with similar expression profile in the comparisons of the Chiquitita-like growth habit, they displayed differential expression in Chiquitita and Arbosana. Therefore, we used these varieties to confirm the expression differences detected through microarray analysis, using quantitative RT-PCR. The results by these two methods displayed a high correlation (*r* = 0.831, critical value 0.708 at α = 0.005) (Figure 5).

**Figure 6:**
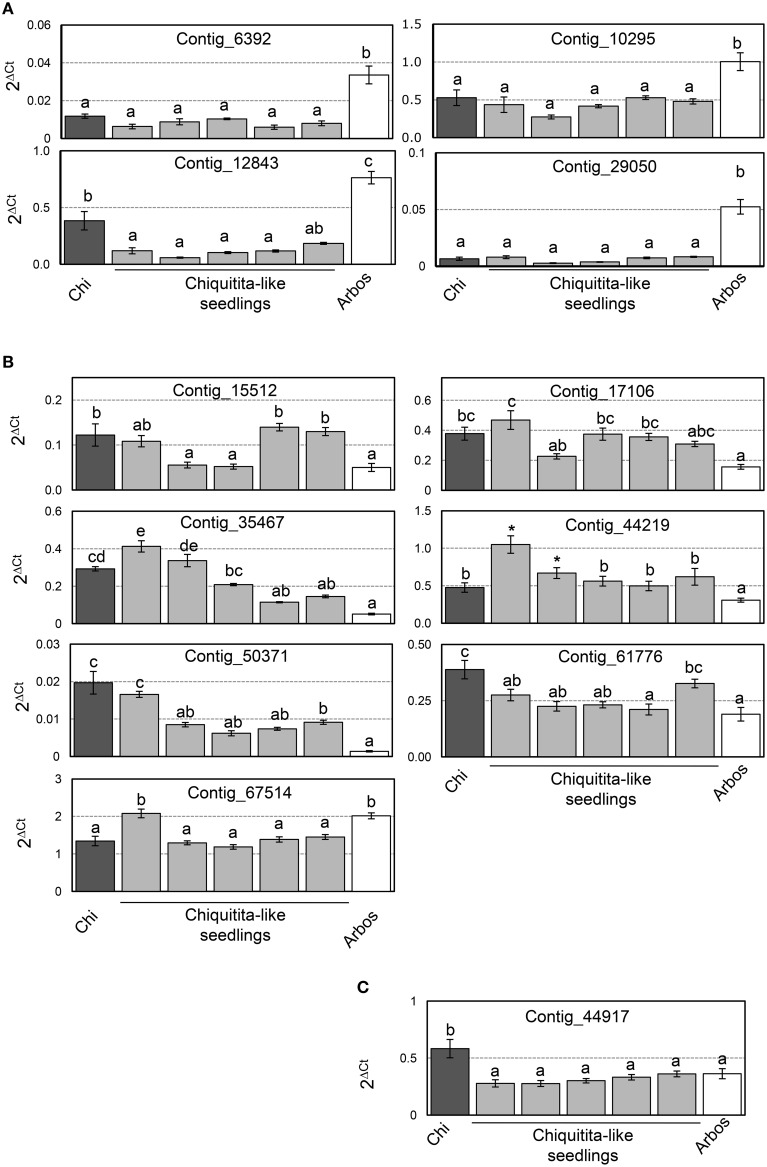
**(A)** Genes whose expression levels were not significantly different from Chiquitita, and significantly lower than Arbosana; **(B)** Genes showing expression levels in most of the ChiCa individuals closer to Chiquitita than to Arbosana; **(C)** Gene, Contig_44917, displaying similar expression in the ChiCa pool, not significantly different from that of Arbosana, and significantly different from that of Chiquitita.

We also analyzed expression of the selected genes in the individual seedlings that form the ChiCa pool, and tested for statistical differences through a Multiple Range test method (95% LSD). Four of the 12 genes analyzed (Contigs 6392, 10295, 12843, and 29050) showed expression levels not significantly different from Chiquitita, and significantly lower than Arbosana (Figure 6A). Seven of the remaining 8 genes (Contigs 15512, 17106, 35467, 44219, 50371, 61776 y 67514) showed expression levels in most of the ChiCa individuals, closer to Chiquitita than to Arbosana (Figure 6B). However, in these cases the differences with Arbosana were not always statistically significant for all the individual seedlings, where expression values for these genes displayed a higher dispersion that for the previous four. Finally, the remaining gene, Contig_44917 displayed unexpected results, since all individuals from the ChiCa pool showed very similar expression, which was not significantly different from that of Arbosana, and significantly different from that of Chiquitita (Figure 6C). This is just the opposite of the results according to the microarray analysis. However, since the expression ratio detected for this gene by RT-*q*PCR assays was very low (Figure 5), significant differences were difficult to establish. Only one other case was found to display a similarly low expression ratio by RT-*q*PCR assays, Contig_67514 (Figure 5). However, for Contig_67514, expression on four out of the five individuals from the ChiCa pool was significantly different to Arbosana as established by a Multiple Range test (95% LSD). Our best explanation for the results obtained for this gene rests on the different level of specificity between the two analyses, and the potential presence of gene duplications for Contig_44917.

### Phenotypic evaluation of *Arabidopsis* mutants in putative orthologs of the candidate genes

Mutants in the putative *Arabidopsis* orthologs of 5 out of the 12 candidate genes used for expression analyses had been previously analyzed for phenotypes related to plant architecture. Mutants in these genes, *IRX1, SYD, SPY, DWF4*, and *SAL1*, display altered phenotypes including stunted or slower growth, as well as other morphological alterations (Table 3). The relative abundance of architecture-related mutant phenotypes for the *Arabidopsis* putative orthologs of the candidate genes selected from a non-targeted analysis, encouraged us to extend our study to investigate the phenotypes of mutants in *Arabidopsis* putative orthologs for additional selected candidate genes, not previously studied in relation to plant architecture.

**Table 3:** **Description of putative orthologs and mutants in *Arabidopsis* of selected olive candidate genes**.

**Olive contig**	**Arabidopsis putative ortholog**	**Gene description**	**Mutant id**.	**Architecture related phenotype**	**References**
6392	AT4G18780	*IRX*1 (*IRREGULAR XYLEM 1*) Cellulose synthase, secondary cell wall biosynthesis	*irx1-1* and *irx1-2*	Slightly smaller, slower growth; collapsed xylem, unable to grow upright, dark green leaves.	Turner and Somerville, 1997
			SALK_026812/*irx1*-5	Dwarf, with characteristic dark green leaves.	Rubio-Díaz et al., 2012
67514	AT2G28290	*SYD* (*SPLAYED*) Encodes a SWI2/SNF2-like protein in the SNF2 subclass. Co-activator of floral homeotic gene expression. Promotes the expression of CUC2 during cotyledon boundary formation. Regulates WUS.	CS693/*syd-2*	Pleiotropic phenotype: slow growth; reduced plant height; leaves and bracts small and upward curling; precocious transition to flower formation under short-day conditions; plants occasionally form a flower immediately from the rosette leaves; defects in floral organ formation; splayed-open floral bud	Kwon et al., 2005
10295	AT3G11540	*SPY* (*SPINDLY*) N-acetyl glucosamine transferase, repressor of GA signaling. Positive regulator of CKs signaling.	CS6266, CS6267 and CS6268	Resemble wild type plants repeatedly treated with GA; long hypocotyls; erect rosette leaves, light green color, early flowering; stem elongation is increased primarily by an increase in internode length; partial male sterility; altered gibberellin signal transduction.	Jacobsen et al., 1996
61776	AT3G50660	*DWF4/SNP2*. 22α-hidroxilase involved in brassinosteroids biosynthesis	*snp2-1/dwf4-101*	Short stem, dark green and round leaves with short petioles. Has more lateral shoots than wild type	Choe et al., 1998; Nakamoto et al., 2006
44917	AT5G63980	*SAL1/FRY1* 3′(2′), 5′-bisphosphate nucleotidase/inositol polyphosphate 1-phosphatase. Bifunctional protein involved in response to ABA response. Regulates the repression of hypocotyl elongation, and flowering. Suppressor of overexpression of PIN1	SALK_151367	Dwarf. Displays alterations associated to sulfur metabolism deficiencies.	Kim and von Arnim, 2009; Zhang et al., 2011; Lee et al., 2012
44219	AT3G25100	*CDC45*, Cell division cycle 45. Required for meiosis, acts in the last round of DNA replication, prior to meiosis.	SALK_128351C	Increased growth/Over-expressed	This work Figure 7
17106	AT3G46600	SCL30 also known as SCL11b. GRAS family transcription factor Scarecrow-like protein 30. Other SCR is involved in the radial shoots and roots pattern.	SALK_139541C	Increased growth	Bolle, 2004; Gao et al., 2004 This work Figure 7
40482	AT5G39360	*EDL2*, EID1-like 2; *EID1* is an F-box protein that functions as a negative regulator in phytochrome A (phyA)-specific light signaling. Forms stable complexes with several ASK proteins and Cullin1.	SALK_097615	Delay in flowering and growth	This work Figure 8
			SALK_018189	Delay in flowering and growth	This work Figure 8
35467	AT2G22540	*SVP*, Short Vegetative Phase. Controls flowering in *Arabidopsis* and *Antirrhinum*.	SALK_072930C	Increased growth	Andrés et al., 2014 This work Figure 8
			*svp*-41	Increased growth	Andrés et al., 2014 This work Figure 8

**Figure 7:**
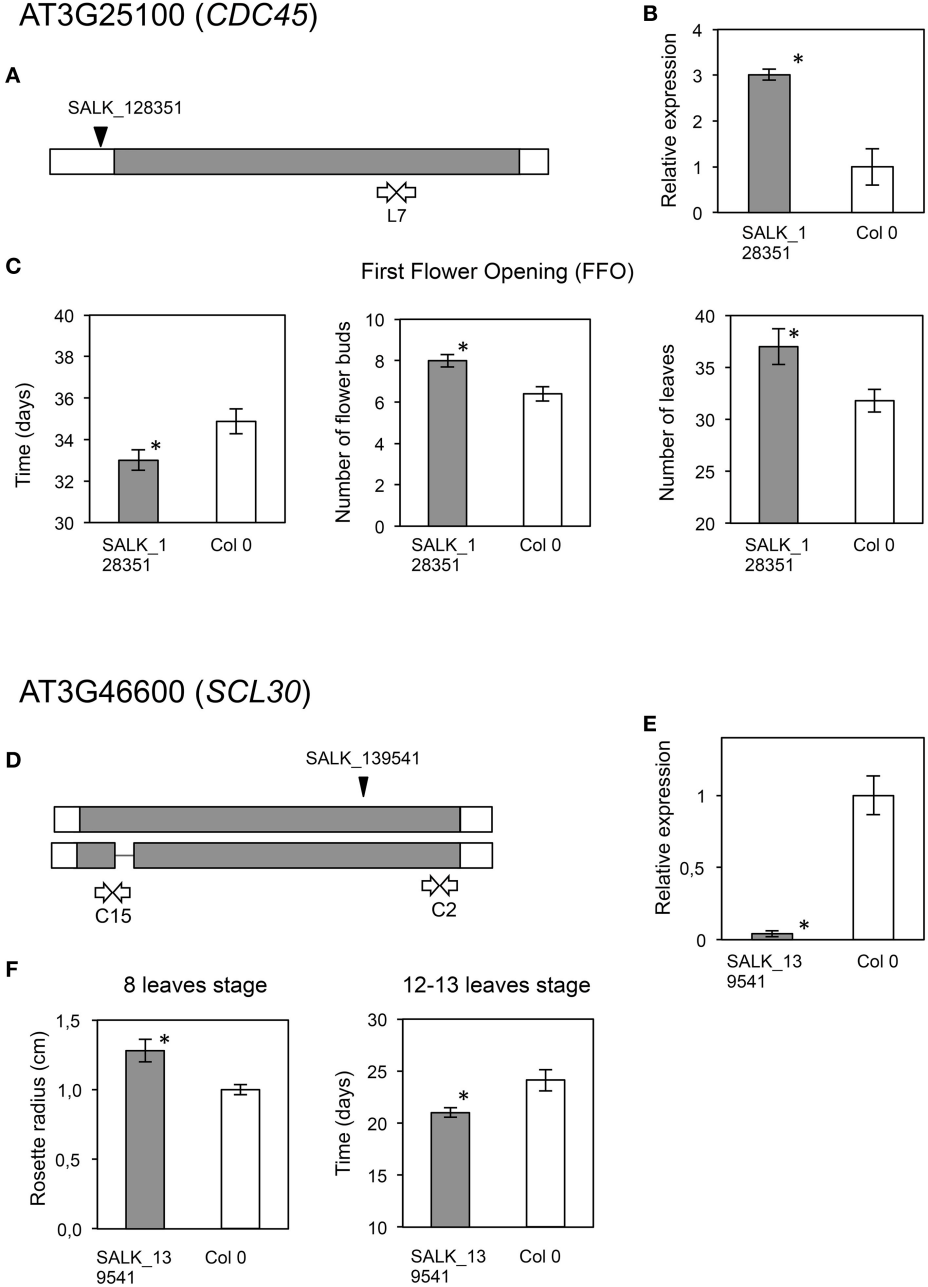
**Phenotypic analyses of Arabidopsis mutants in AT3G25100 (*CDC45*) and AT3G46600 (*SCL30*), putative orthologs of olive candidate genes**. Upper panel displays data regarding AT3G25100 (*CDC45*). **(A)** Location of the T-DNA insertion (black arrowhead), and primers used for expression analysis (white arrows). Coding area is shaded. **(B)** RT-*q*PCR assays of transcript accumulation in mutant (gray) (*P* = 0.0086) and wild type plants (white). **(C)** Graphs displaying those phenotypes for which significant phenotypic differences were found between the mutant and wild type lines. Asterisks indicate statistically significant differences (left, *P* = 0.0276; center, *P* = 0.0082; right, *P* = 0.0123). Lower panel displays data regarding, AT3G46600 (*SCL30*). **(D)** Location of the T-DNA insertion (black arrowhead), and primers used for expression analysis (white arrows). Coding area is shaded. **(E)** RT-*q*PCR assays of transcript accumulation in mutant (gray) (*P* = 0.0021) and wild type plants (white). **(F)** Graphs displaying those phenotypes for which significant phenotypic differences were found between the mutant and wild type lines. Asterisks indicate statistically significant differences (left, *P* = 0.0369; right, *P* = 0.0426).

**Figure 8:**
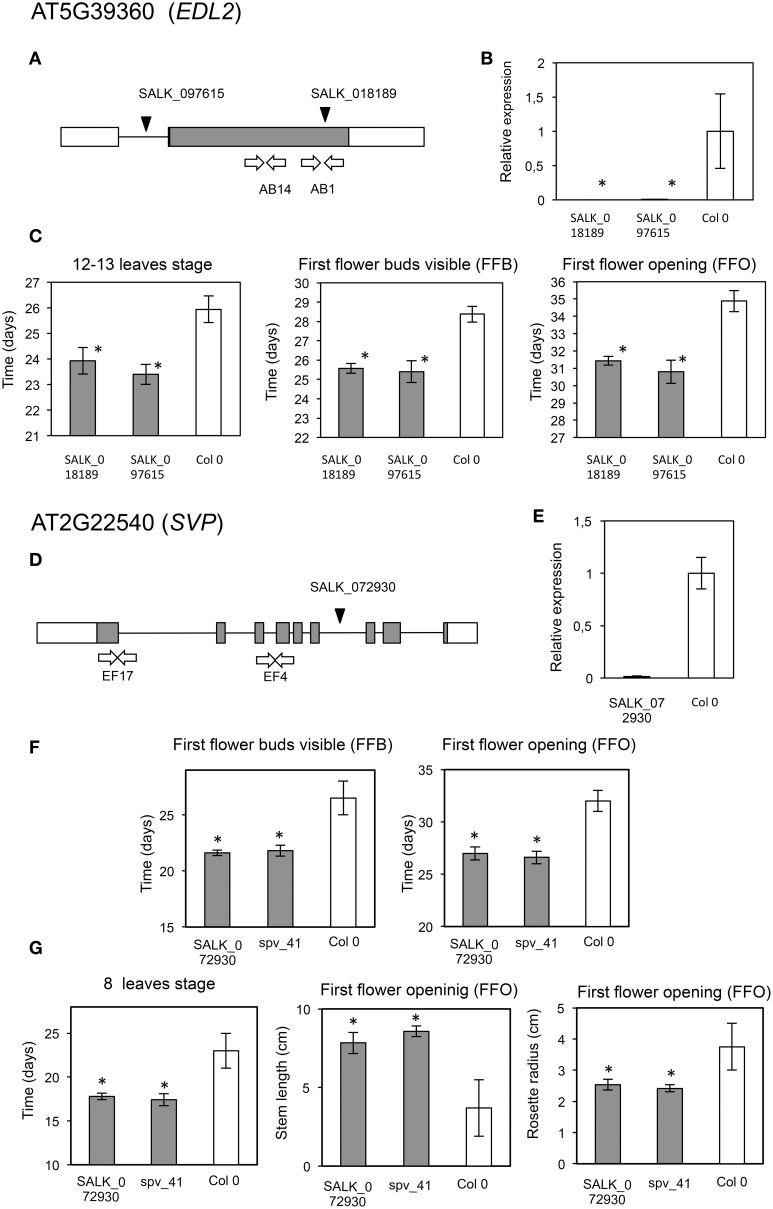
**Phenotypic analyses of mutants in AT5G39360 and AT2G22540**. Description of AT5G39360 (*EDL2*) mutant analyses (upper panel). **(A)** Location of the T-DNA insertions (black arrowhead), and primers used for expression analysis (white arrows). Coding area is shaded. **(B)** RT-*q*PCR assays of transcript accumulation in mutant (gray) (SALK_018189, *P* = 4.32 × 10^−5^, and SALK_097615, *P* = 0.0015) and wild type plants (white). **(C)** Graphs displaying those phenotypes for which significant phenotypic differences were found between the mutants and wild type lines. Asterisks indicate statistically significant differences (One Way ANOVA: left, *P* = 0.0126; center, *P* = 0; right, *P* = 0) (Multiple Range Test 95% LSD after significant *P*-value at One Way ANOVA). Description of AT2G22540 (*SVP*) mutant analyses (lower panel). **(D)** Location of the T-DNA insertion (black arrowhead), and primers used for expression analysis (white arrows). Coding area is shaded. **(E)** RT-*q*PCR assays of transcript accumulation in mutant (gray) (*P* = 8.34 × 10^−9^) and wild type plants (white). **(F)** Graphs displaying those phenotypes associated to flowering for which significant phenotypic differences were confirmed between the mutant and wild type lines. Asterisks indicate statistically significant differences (left, *P* = 0; right, *P* = 0.0001). **(G)** Graphs displaying those phenotypes associated to architecture for which significant phenotypic differences were found between the mutant and wild type lines. Asterisks indicate statistically significant differences (One Way ANOVA: left, *P* = 0.0200; center, *P* = 0; right, *P* = 0.0009) (Multiple Range Test 95% LSD after significant *P*-value at One Way ANOVA).

A mutant in *CDC45* (putative ortholog of *Contig*_44219), a gene reported as involved in DNA replication during meiosis in flower buds (Stevens et al., 2004), showed early flowering and significantly higher numbers of leaves (*P* = 0.012; α = 0.05) and flower buds at first flower opening (*P* = 0.008; α = 0.05) (Table 3, Figures 7A–C). The insertion in this mutant is located in the promoter region, and transcript accumulation was found to be considerably higher than in the wild type genotype. We also found a significant increase in growth (*P* = 0.037; α = 0.05; and *P* = 0.043; α = 0.05) for a mutant in *SCL30*, a previously uncharacterized putative transcriptional regulator of the GRAS family (Table 3, Figures 7D–F), and the putative ortholog of Contig_17106. Mutants in *EDL2* (both homozygous and heterozygous) displayed early flowering and faster growth (Multiple Range test, LSD, method 95%), with significant reductions in the times for developing the first flower bud, and for reaching the 12-13 leaves stage (Table 3, Figures 8A–C). *EDL2* encodes an EID1-like protein, with an F-box domain and is the putative ortholog of *Contig*_40482. We also included in our analysis a mutant in *SVP1* (*SHORT VEGETATIVE PHASE 1*), the putative ortholog of *Contig*_35467, which encodes a well characterized repressor of flowering (Hartmann et al., 2000; Table 3, Figures 8D–G). As previously reported, mutant *svp_41* displayed early flowering (Figure 8F), however, we observed that it also displayed faster growth as it reached the 8 leaves stage significantly sooner (One way ANOVA, *P* = 0.02; α = 0.05; Multiple Range test, LSD, method 95%), and presented significantly longer stems at first flowering (One way ANOVA, *P* = 0; α = 0.05; Multiple Range test, LSD, method 95%), even though it reaches this stage considerable quicker (One way ANOVA, *P* = 0.0001; α = 0.05; Multiple Range test, LSD, method 95%) (Figure 8G; González-Plaza, 2013). Finally, no phenotypic differences were found for the mutant in *TPR1* (data not shown), putative ortholog of *Contig*_*50371*, however the insertion in this mutant is located in the promoter region, and mRNA accumulation was found to be just slightly higher than that of the wild type (Table S5).

In summary, four out of five of the *Arabidopsis* mutants analyzed in this study, and 9 out of the 10 analyzed in total, in this or previous studies, from the putative orthologs of the 12 selected candidate genes, displayed phenotypic changes in relation to the wild type related to architecture.

## Discussion

### Transcriptomic analysis

In this study, aimed to identify genes responsible for phenotypic differences among different genotypes, the challenge was to identify among all DEGs between genotypes, those related to their differences in plant architecture. A similar approach was previously used in *Populus* (Street et al., 2006) and *Eschscholzia californica* (Zahn et al., 2010). It is based on the use of an interwoven loop design and pools of individuals sharing a relevant phenotype selected among the segregating progeny from a given cross. The analysis of pools of individuals from the same cross sharing a relevant phenotype is also the basis of bulked segregant analysis (BSA), a strategy frequently applied to the identification of genetic markers associated to a mutant phenotype that uses DNA-based analysis (Michelmore et al., 1991). Although this study is closer to a transcriptomic analysis than to a BSA, the selection of pools was also an important aspect of the study, particularly since different genotypes and not treatments or experimental conditions were compared. The comparisons carried out rendered lists of candidate genes of varying lengths, ranging from 23, obtained through internode length-based comparisons, to 1957, obtained through growth habit-based comparisons, and included 299 identified through diameter size-based comparisons. These varying numbers could be the consequence of the genetic characteristics of the traits analyzed in each case, or could reflect differences in genetic distances among the genotypes studied. In growth-habit comparisons, descendants were compared to their parents, whereas the other two comparisons were always carried out among siblings. Many of the DEGs selected as candidate genes were obtained from the growth habit-based comparisons, and displayed significant differences in expression between Chiquitita and Arbosana. Arbosana displays moderate vigor and although it is not entirely unsuited for higher-than-traditional planting densities, its vigor is clearly higher than that of Chiquitita, and its growth habit markedly different. The results of the statistical analysis of the candidate gene sets obtained, as well as those obtained from the analysis of their functional annotation, support that the genes identified are indeed involved in functions related to determining tree architecture.

### Putative orthologs in *Arabidopsis* with reported architecture mutant phenotypes

Approximately fifty percent of the 2252 candidate genes have potential orthologs in *Arabidopsis*. Since functional validation of candidate genes in olive is still very difficult, we looked for further support of the biological relevance of our results by analyzing plant architecture-related phenotypes in mutants of the putative *Arabidopsis* orthologs of the selected olive candidate genes. Although an architecture-related phenotype in the *Arabidopsis* mutants in ortholog genes does not constitute a demonstration of function of the candidate genes in olive, it adds support to the validity of the approach taken to identify genes related to determining plant architecture in olive. Mutants in the putative orthologs of five out of the 12 candidates genes selected had already reported plant architecture-related phenotypes. One such example is *IRX1*, which encodes an enzyme of the cellulose biosynthetic pathway, necessary for growth (Rubio-Díaz et al., 2012). We found significantly lower levels (*P* = 7.55 × 10^−4^) of gene transcripts of the putative olive ortholog of *IRX1* (Contig_6392) in Chiquitita. This results is indeed very interesting since suppressed expression of cellulose synthase genes has been previously associated to weeping canopies in poplar (Joshi et al., 2011; Lu et al., 2015). Thus, decreased expression of this enzyme might be contributing to the reduced size and/or the unusual weeping canopy of this olive variety.

Another such example is *Splayed* (*SYD*), which encodes a positive regulator of *WUS* (Wang and Li, 2008), the gene encoding the direct activator of meristematic growth (Turnbull, 2005). It is noteworthy that despite the well-established central role for *WUS* in controlling meristem activity in *Arabidopsis*, little is known about the role of its homologs in trees. The putative olive ortholog of *SYD* (Contig_67514) displayed significantly lower levels in Chiquitita (*P* = 2.54 × 10^−3^). This result is in keeping with the reduced size of this variety and opens the possibility of functional conservation for the WUS-CLV pathway in olive. Supporting this notion, a putative olive ortholog of the gene encoding the WUS repressor *Clavata1* (*CLV1*) (Contig_43041) shows significantly higher levels of gene transcript in Chiquitita than in Arbosana in our microarray analysis (Student's *T*-test, data not shown/ GSE60284) in keeping with a role as inhibitor of meristem growth (Turnbull, 2005).

Other selected genes with functions related to plant architecture functions include *SPY*, which encodes a GA response repressor in *Arabidopsis* (Jacobsen et al., 1996; Swain et al., 2002; Filardo and Swain, 2003; Greenboim-Wainberg et al., 2005), and thus acts as a positive regulator of cytokinins (CKs), promoting growth in axillary meristems in this species (Greenboim-Wainberg et al., 2005). Interestingly, gibberellins has been shown to display opposite roles in the regulation of shoot branching, inhibiting this process in annual plants such as pea or *Arabidopsis*, or perennials such as turfgrass or *Populus* (Scott et al., 1967; Koornneef et al., 1985; Agharkar et al., 2007; Mauriat et al., 2011; Zawaski and Busov, 2014), or stimulating it in snapdragon, or woody species such as citrus, sweet cherry or *Jatropha curcas* (Marth et al., 1956; Elfving et al., 2011; Ni et al., 2015). The elevated levels of gene transcript found for its putative olive ortholog (Contig_10295) in Arbosana, are in keeping with a conserved role in the activation of CKs synthesis in olive, and suggests a role for gibberellins in stimulating shoot growth in this species, similar to what has been described for trees such as citrus or sweet cherry (Marth et al., 1956; Elfving et al., 2011).

Other putative ortholog with reported architecture mutant phenotypes is *SAL1*. The protein encoded by *SAL1* acts through the inositol signaling pathway, modulating auxin transport (Zhang et al., 2011), and has been described as a suppressor of the effects associated to the over-expression of *PIN1* (Vernoux et al., 2000), an auxin transporter in *Arabidopsis* (Zhang et al., 2011). Interestingly, low levels of transcript for *PINL1*, an ortholog of *Arabidopsis* auxin efflux carrier, have been very recently proposed to prevent auxin polar transport in hybrid aspen, inhibiting branching and apical expansion (Rinne et al., 2015). If similar regulatory pathways were conserved in olive, the significantly higher levels of transcript for the olive putative ortholog of *SAL1* (Contig_44917) measured in Chiquitita would be consistent with its reduced growth as it could interfere with auxin polar transport in this variety.

The last of the putative orthologs of selected candidate genes with a reported architecture related mutant phenotype is *DWF4*, which encodes an enzyme that acts as limiting step in the synthesis of BRs in *Arabidopsis* (Choe et al., 2001). BRs stimulate stem elongation at extremely low physiological concentrations (Clouse et al., 1996; Clouse and Sasse, 1998; Wang and Li, 2008), with an excess of hormone repressing growth and development in *Arabidopsis* (Yuan et al., 2007). Transcript levels of its ortholog in olive were found to be significantly higher in Chiquitita. If elevated transcript levels led to elevated concentrations of BRs, it could restrict stem elongation potentially contributing to reduce growth in this variety.

### Putative orthologs in *Arabidopsis* with newly identified architecture mutant phenotypes

When we analyzed the mutant phenotypes of *Arabidopsis* orthologs of additional selected candidate genes, not previously studied in relation to plant architecture, we found architecture-related phenotypes for four out the five genes studied. A mutant carrying a T-DNA insertion in the promoter region of *CDC45*, causing a significant increase in transcript accumulation, showed early flowering and significantly higher numbers of leaves and flower buds at first flower opening (*P* = 0.0276, *P* = 0.0124, and *P* = 0.0082, respectively) (Figure 7C). *CDC45* is upregulated during the G1 to S transition in young meiotic flower buds and a reduction in its expression is associated to sterility (Stevens et al., 2004). A role in regulating flower bud meiotic activity would explain the early flowering phenotype found for this line, however how *CDC45* functions to determine changes in plant growth is still to be established in any species.

We also found a significant increase in growth (*P* = 0.037, and *P* = 0.043) (Figure 7F) for a mutant in *SCL30*, a previously uncharacterized putative transcriptional regulator of the GRAS family, which includes negative regulators of GA responses (Pysh et al., 1999; Gao et al., 2004; Turnbull, 2005) and thus, of stem elongation and growth in *Arabidopsis*.

A role in repressing growth might be carried out also by the putative olive ortholog of *SCL30*, Contig_17106, since its expression is significantly higher in Chiquitita than Arbosana (Figures 5B, 6B). A similar reasoning can be applied to mutants in *EDL2* (both homozygous and heterozygous), which displayed early flowering and faster growth (*P* = 0, and *P* = 0.0126, respectively) (Figure 8C), thus supporting a role in negatively regulating growth in *Arabidopsis*. *EDL2* encodes an EID1-like protein, with an F-box domain, and homology to *EDL1* and *EDL3* genes. However, the expression of the putative putative olive ortholog to *EDL2* (*Contig*_40482) is significantly higher in Arbosana than in Chiquitita, supporting a potential role for this gene in activating, rather than repressing growth. This result could suggest that the role of EDL2 in controlling growth in olive may differ from that in the annual plant *Arabidopsis*.

We also included in our phenotypic assays *svp_41*, a well-characterized mutant of *SVP1*, which encodes a repressor of flowering (Hartmann et al., 2000). Mutants in this gene have been extensively characterized for their early flowering phenotype, but a link to a role in directly determining plant architecture had not been described when this work was carried out. Our assays showed a significant increase in growth, as an increase in stem length at first flower opening, for the mutant (*P* = 0) (Figure 8G; González-Plaza, 2013). A recent report (Andrés et al., 2014) has shown that SVP1 reduces gibberellin biosynthesis at the shoot apex, thus explaining the increased height observed in our study. The significantly higher transcript levels (*P* = 3.6 × 10^−4^) found for the *SVP1* putative ortholog, Contig_35467 in Chiquitita, consistent with the reduced size of this variety, supports the potential conservation of the role of SVP in regulating gibberellin biosynthesis at the shoot apex in olive.

Finally, we found no significant phenotypic differences for the mutant in *TPR1* (topless related) encodes another repressor of *WUS* (Causier et al., 2012), adding its effects to those of *CLV1*. A dwarf phenotype has been previously described for an over-expression line for *TPR1* in *Arabidopsis* (Zhu et al., 2010). However, the insertion in this mutant is located in the promoter region and mRNA accumulation was just slightly higher than wild type. Interestingly, the expression of the *TPR1* putative ortholog in olive (Contig_50371) is significantly higher in Chiquitita (*P* = 2.95 × 10^−4^), which would be in agreement with its having a role in repressing meristematic activity and growth in olive.

### Future research

In summary, the experimental approach taken in this study has allowed us to identify genes associated to plant architecture in olive and of potential interest for potential future application to breeding through marker-assisted selection (MAS). In this last regard, differences in the expression of four of the selected genes (Contigs 6392, 10295, 12843, and 29050) did discriminate every seedling from the Chiquitita-like canopy pool from that of the parental Arbosana. Characterization of architecture phenotypes for previously uncharacterized *Arabidopsis* mutants support the relevance of this type of studies in providing basic developmental knowledge not only relevant for trees but even in for the well-known *Arabidopsis* model. The recent efforts from many research groups in gathering genomic information for tree species (Costes and Gion, 2015), including fruit trees, provides an excellent framework for further work on establishing additional functional connections with gene candidate orthologs in these species and for cross-reference of functional data in relation to genetic determination of tree architecture. They could also help in the functional annotation of the many candidate genes of unknown functions, identified through the microarray analysis in this study. These genes not analyzed further in the present study, are potentially interesting targets for future work, both for functional characterization of their role in plant architecture, and because the large differences in expression displayed by many of them makes them strong candidates for MAS. Finally, further analysis will be necessary to establish how early during plant development can expression differences be confidently established for the different candidate genes to fine-tune their applicability as expression markers, or to associate differences in expression to differences in sequence that could be used as traditional makers for breeding. This work represent the first step on the identification of genes related to tree architecture in this species of agronomical relevance. Furthermore, it can be of utmost interest to increase the efficiency of future breeding programs aimed to produce varieties adapted to high-density growing systems.

## Accession numbers

GK-339E12.01 (N721450); SALK_046685C (N679626); SALK_026812 (N526812); SALK_090582C (N676168); CS6266 (N6266); SALK_022185C (N674070); SALK_090580 (N590580); SALK_039417C (N662173); SALK_064305 (N564305); SALK_023032 (N523032); SALK_139541C (N657835); SALK_141675 (N641675); SALK_072930C (N666411); SALK_120540 (N620540); SALK_128351C (N676874); SALK_065650C (N659435); SALK_024358C (N659341); CS6938 (N6938); SALK_151367 (N651367); SALK_020882 (N520882); SALK_018189(N518189); SALK_097615C(N676314). The data discussed in this publication have been deposited in NCBI's Gene Expression Omnibus (Edgar et al., 2002) and are accessible through GEO Series accession number GSE60284.

## Author contributions

Experimental design and/or overall work strategy: JG, AM, JS, FL, OT, RR, VV, and CB. RR and CB carried out the sample selection, and JG, RR, and IO field sample collection. JG, AM, IO, and CB performed the microarray analysis. Statistical work was carried out by JG, IO, JS, and CB. AM, OT, JG, and CB carried out functional annotation. JG, IO, CG, FL, and CB performed the expression assays for the candidate genes in olive samples and the corresponding analysis. Experimental design for *Arabidopsis* experiments was the responsibility of JG, EB, and CB. Experimental work in *Arabidopsis* was carried out by JG, and analyzed by JG, EB, and CB. JG, EB, and CB wrote the manuscript. All authors critically reviewed the manuscript prior to submission. All authors read and approved the final version of the manuscript. All authors agreed to be accountable for accuracy, integrity and appropriateness of the manuscript. Authors initials in order: JG, IO, AM, CG, JS, FL, OT, EB, RR, VV, CB.

### Conflict of interest statement

The authors declare that the research was conducted in the absence of any commercial or financial relationships that could be construed as a potential conflict of interest.
