# Supplementary material for: Transcriptomic Analysis Using Olive Varieties and Breeding Progenies Identifies Candidate Genes Involved in Plant Architecture
Source: Front Plant Sci. 2016 Mar 2;7:240. doi: 10.3389/fpls.2016.00240 (PMC4773642; doi:10.3389/fpls.2016.00240)
Supplement: Supplementary file 1 [file Table1.docx]

**Table S1.** Primers used in this study. (No. Number; Seq. Sequence).

| **Primers used for genotyping of *Arabidopsis* mutants*thaliana*** | | | | | | | | | | | | | | |
| --- | --- | --- | --- | --- | --- | --- | --- | --- | --- | --- | --- | --- | --- | --- |
| **Line** | | **No.** | | **Sequence** | | | | **No.** | | **Sequence** | | | **Putative ortholog** | |
| SALK_141675.44.50.n | | LP6 | | CGGAAAACTGTTCGAGTTCTG | | | | RP6 | | AAAATGTGTGGTCGTTTCGAG | | | Contig_35467 | |
| SALK_072930.51.80.x | | LP7 | | TCATCCATATCTTGCAATGCC | | | | RP7 | | TCAGCGAACTTCAGAAAAAGG | | | Contig_35467 | |
| SALK_039417.54.15.x | | LP8 | | GATGATGCCATGGAAAAATTG | | | | RP8 | | AAGCTGAGCGATGTCTGATTC | | | Contig_29050 | |
| SALK_064305.52.50.x | | LP9 | | GATGATGCCATGGAAAAATTG | | | | RP9 | | GATCTCACAGCTCTCTGACGC | | | Contig_29050 | |
| SALK_065650.45.45.x | | LP10 | | AAGGCCTCGAGATACTTCTGC | | | | RP10 | | ACTCCGTTATCCGTCACCTTC | | | Contig_50371 | |
| SALK_024358.15.75.x | | LP11 | | TGGCATTCAATCCTTAGTTCG | | | | RP11 | | GCTCAGGTGCTGATTCATCTC | | | Contig_50371 | |
| SALK_026812.38.65.x | | LP12 | | AATCACAACCGAAAGCACATC | | | | RP12 | | TTCCGATTTTTCACAATCCAC | | | Contig_6392 | |
| SALK_026812.38.65.x | | LP13 | | ACAGGATGCGTGATACGGTAG | | | | RP13 | | ATCCATCCAAATCTCAATCCC | | | Contig_6392 | |
| GK-339E12.01 | | LP14 | | ATCGCCGACAAACTACACATC | | | | RP14 | | TAAACCACAAAATGGCGAAAC | | | Contig_6392 | |
| SALK_046685.56.00.x | | LP16 | | GAAATTTGTGTCGAGACCAGC | | | | RP16 | | TACAGTCCACCTTCAAAACCG | | | Contig_6392 | |
| SALK_151367.44.90.x | | LP17 | | CTAACGGCAAGTTTGGACAAG | | | | RP17 | | TCCGCTTCAATCATTTGTAGC | | | Contig_44917 | |
| SALK_020882.55.50.x | | LP18 | | CTTCGAATGACTCGAAGAACG | | | | RP18 | | TTTTCGATTCAATCATGACCC | | | Contig_44917 | |
| SALK_097615.55.50.x | | LP19 | | CACCAACTCTTCAGCTTCGTC | | | | RP19 | | CACTCATCATCTTAGGCGCTC | | | Contig_40482 | |
| SALK_018189.56.00.x | | LP21 | | AATATCGTTGCATTCACTCGG | | | | RP21 | | AACCAGGTCATAGCACAATCG | | | Contig_40482 | |
| SALK_023032.16.55.x | | LP22 | | CAGGTTTCCAGCAAGAAACAG | | | | RP22 | | TGGTTATCACTTTGCTGAGGC | | | Contig_17106 | |
| SALK_139541.33.30.x | | LP23 | | ACAAGGTTACCGTGGGGTAAG | | | | RP23 | | AGCTTCCTCAGTCAGGTTTCC | | | Contig_17106 | |
| SALK_022185.53.30.x | | LP24 | | TCAAATCTCCTAAATGGCTGG | | | | RP24 | | TAGCCTCATAGAGAGCAAGCG | | | Contig_10295 | |
| SALK_090582.49.05.x | | LP25 | | ATCTGAGTCGTTGCCTCTGTC | | | | RP25 | | TGTAATGTTGCACCTCAGCAG | | | Contig_10295 | |
| SALK_090580.27.40.x | | LP26 | | GACCGACCTCGTACCTCTTTC | | | | RP26 | | ATCTGCCATCAATGCTTTCTG | | | Contig_10295 | |
| SALK_120540.53.80.x | | LP31 | | AAACGAATTGTTCAACCCTTTC | | | | RP31 | | AAGCGCACAAAGTGAATCAAC | | | Contig_44219 | |
| SALK_120540.53.80.x | | LP32 | | TTGAAAGAGTTGAATGATTTCATCTC | | | | RP32 | | AAGCGCACAAAGTGAATCAAC | | | Contig_44219 | |
| SALK_128351.53.00.x | | LP33 | | AAACGAATTGTTCAACCCTTTC | | | | RP33 | | AAGCGCACAAAGTGAATCAAC | | | Contig_44219 | |
| SALK_128351.53.00.x | | LP34 | | TTGCTTTTAACTTTGGTTTCTCG | | | | RP34 | | AAGCGCACAAAGTGAATCAAC | | | Contig_44219 | |
| *svp-41* | | svp41f | | ttcggggacatacacactttc | | | | svp41r | | gtaccttagtcggtggctcttg | | | Contig_35467 | |
| **Vector** | | | | | **Mutant line** | | | | **Sequence** | | | | |  |
| LBb1.3 | | | | | Salk | | | | ATTTTGCCGATTTCGGAAC | | | | |  |
| Sul2 | | | | | Gabi-Kat | | | | GTCGAACCTTCAAAAGCTGAAGT | | | | |  |
| Sul4 | | | | | Gabi-Kat | | | | ATTTCACACAGGAAACAGCTATGA | | | | |  |
| **Primers used for RT-qPCRs in olive** | | | | | | | | | | | | | | |
| **Forward** | **Sequence** | | | | | | **Reverse** | | | | | **Sequence** | | |
| Contig_F_44917 | TTCCACGGTCAATGGCATCGAGC | | | | | | Contig_R_44917 | | | | | GAAACCCGAGGAACACTAGTGCGC | | |
| Contig_F_67514 | GGCTGCTCTCCAATGTTCTC | | | | | | Contig_R_67514 | | | | | AAAGGAGGCTAAGGTGTTGGA | | |
| Contig_F_35467 | TGCCAGCTCCAGCATGAAGGACATA | | | | | | Contig_R_35467 | | | | | ACTGTCCTCGACTAGCTGCTTCGG | | |
| Contig_F_29050 | CTTGCTGCTGTAGTTGAGCCTA | | | | | | Contig_R_29050 | | | | | ATTCGCGATATGTGTGATTCTG | | |
| Contig_F_50371 | TGATCTATGTCCAGTGCTTCTAGG | | | | | | Contig_R_50371 | | | | | GGAAAGGTACTTATTGGCATTCAC | | |
| Contig_F_61776 | TCCTCTACAAGCCACCTGCT | | | | | | Contig_R_61776 | | | | | TGTCTGCCTTTGTGAAGGTATG | | |
| Contig_F_17106 | TGGTACCACTTACGGGAAGG | | | | | | Contig_R_17106 | | | | | GGATCATCGGAGAAATTAGCC | | |
| Contig_F_10295 | ATTGATACAGAGGGCCAACG | | | | | | Contig_R_10295 | | | | | CGGCAACACTGAGATTTTGA | | |
| Contig_F_12843 | GGGGCTAGGAATTGGTCTATG | | | | | | Contig_R_12843 | | | | | TACTTTGTGTGCGCCTTCAG | | |
| Contig_F_6392 | AGCAAATCGTGCACTCACTG | | | | | | Contig_R_6392 | | | | | CTATCCGCCGTAGAGACACTG | | |
| Contig_F_44219 | TCTCAAGGATGGGACAAAGG | | | | | | Contig_R_44219 | | | | | GTTGCAATGTAGGACGAGCA | | |
| Contig_F_15512 | CAATTGAAGTGGGTTGTGCTT | | | | | | Contig_R_15512 | | | | | AACAATCAATTCCACGAGCAG | | |
| **Primers used for RT-qPCRs in mutants of *Arabidopsis thaliana*** | | | | | | | | | | | | | | |
| **Mutant** | | | **Primer pair** | | | **Primer Forward sequence** | | | | | **Primer Reverse sequence** | | | |
| SALK_018189 | | | AB1 | | | TTCAGGCGAAAATGATACCAC | | | | | GCCCCTTCAGAATCAGACAA | | | |
| SALK_097615C | | |  |  |  |  |  |  |  |  |  |  |  |  |
| SALK_139541C | | | C2 | | | ATTGCAGGACACTGGTGGA | | | | | CGCTGGTCTAAATCCTGCTC | | | |
| SALK_020882 | | | D3 | | | CGGACGCAAGTCTTCTTCTC | | | | | ATAATCAGCAACGGTCACTGG | | | |
| SALK_072930C | | | EF4 | | | GAAGAGAACGAGCGACTTGG | | | | | GAGCTCTCGGAGTCAACAGG | | | |
| *svp-41* | | |  |  |  |  |  |  |  |  |  |  |  |  |
|  | | |  |  |  |  |  |  |  |  |  |  |  |  |
| SALK_128351C | | | L7 | | | AGGTTCGAGCACCAGACTGT | | | | | CCATACGCGCTAGAAGAAGC | | | |
| SALK_090582C | | | MÑO8 | | | TGAAAGATCCAGAGCGACCT | | | | | ACCGCTGAATAAACCACCAC | | | |
| CS6266 | | |  |  |  |  |  |  |  |  |  |  |  |  |
| SALK_022185C | | |  |  |  |  |  |  |  |  |  |  |  |  |
| *snp2-1*/*dwf4-101* | | | P9 | | | TCGCAAAGCACTCAAAGATG | | | | | TCCCCACGTCGAAAAACTAC | | | |
| SALK_039417C | | | R10 | | | GTTACCACACATCGCGTCAG | | | | | CAGCTCCGAACGGTATTAGC | | | |
| GK-339E12.01 | | | STV11 | | | ATGGATTTACGGGTCGATCA | | | | | GATCTCAACCGAGCCAAGAG | | | |
| SALK_046685C | | |  |  |  |  |  |  |  |  |  |  |  |  |
| SALK_026812 | | |  |  |  |  |  |  |  |  |  |  |  |  |
| SALK_065650C | | | W12 | | | TCCTTCCTCTGTCCCACATC | | | | | AGGTCTGGTTCGCTTTGAGA | | | |
| SALK_018189 | | | AB14 | | | TCTTCCTCCACAATGTCGAAC | | | | | AAAATCCCACGGAAGAATCC | | | |
| SALK_097615C | | |  |  |  |  |  |  |  |  |  |  |  |  |
| SALK_139541C | | | C15 | | | TGGCGAGTTTTGACCAGAG | | | | | TTGTACGCCTTCAAAATGTCC | | | |
| SALK_020882 | | | D16 | | | GAGGCATCGTTCTTCGAGTC | | | | | TCGCGGTATCCTTTATGAGG | | | |
| SALK_072930C | | | EF17 | | | CCACCGGAAAACTGTTCG | | | | | GCTGATCAAGCTTCTCCAAGTT | | | |
| *svp-41* | | |  |  |  |  |  |  |  |  |  |  |  |  |
| SALK_090582C | | | MÑO20 | | | TGGAAAAGGGATATGCTTGC | | | | | TCTGCCATCAATGCTTTCTG | | | |
| CS6266 | | |  |  |  |  |  |  |  |  |  |  |  |  |
| SALK_022185C | | |  |  |  |  |  |  |  |  |  |  |  |  |
| snp2-1/dwf4-101 | | | P21 | | | TCGATGCTTGTTCTTGTTGG | | | | | CTTTTTGGCCTCGTCTTGAG | | | |
| SALK_039417C | | | R22 | | | AGCCACTGTCCTCTTCCTCA | | | | | GGTTCGATGAGGCTTAGTGC | | | |
| GK-339E12.01 | | | STV23 | | | AGAAGGTCGAAGAATTTGCTTG | | | | | ATGAATCCCTGAATCCTGAGAA | | | |
| SALK_046685C | | |  |  |  |  |  |  |  |  |  |  |  |  |
| SALK_026812 | | |  |  |  |  |  |  |  |  |  |  |  |  |

**Table S2**. Measurements taken for the phenotypic characterization of the *Arabidopsis* mutants.

| **Trait** | **Measurements** | | | | |
| --- | --- | --- | --- | --- | --- |
| 4 leaves stage | Days | **-** |  |  |  |
| 8 leaves stage | Days | Rosette radius |  |  |  |
| 12-13 leaves stage | Days |  |  |  |  |
| First Flower Bud (FFB) | Days |  |  |  |  |
| First Flower Opening (FFO) | Days | Rosette radius | Stem length | Number of Flower Buds (FB) | Leaves number |

**Table S3**. Pearson correlation coefficients obtained for each pair of biological replicates in the array (1-2, 1-3, and 2-3).

| **Exp.** | **Biol. rep..** | **r-value*** | **Conf. Interval**** |  | **Exp.** | **Biol. rep.** | **r-value*** | **Conf. Interval**** |
| --- | --- | --- | --- | --- | --- | --- | --- | --- |
| Arbos | 1-2 | 0.9678 | 0.9669 to 0.9686 |  | LILe | 1-2 | 0.9800 | 0.9794 to 0.9805 |
|  | 1-3 | 0.9559 | 0.9547 to 0.9570 |  |  | 1-3 | 0.9481 | 0.9467 to 0.9494 |
|  | 2-3 | 0.9457 | 0.9443 to 0.9471 |  |  | 2-3 | 0.9457 | 0.9443 to 0.9471 |
| xArbe | 1-2 | 0.9763 | 0.9757 to 0.9770 |  | SILe | 1-2 | 0.9537 | 0.9524 to 0.9548 |
|  | 1-3 | 0.9862 | 0.9858 to 0.9866 |  |  | 1-3 | 0.8523 | 0.8486 to 0.8559 |
|  | 2-3 | 0.9764 | 0.9758 to 0.9770 |  |  | 2-3 | 0.8466 | 0.8428 to 0.8503 |
| Pica | 1-2 | 0.9779 | 0.9773 to 0.9785 |  | Chi | 1-2 | 0.9741 | 0.9734 to 0.9748 |
|  | 1-3 | 0.9361 | 0.9344 to 0.9377 |  |  | 1-3 | 0.9163 | 0.9141 to 0.9184 |
|  | 2-3 | 0.9630 | 0.9620 to 0.9639 |  |  | 2-3 | 0.9358 | 0.9342 to 0.9375 |
| SmaD | 1-2 | 0.9912 | 0.9910 to 0.9915 |  | ChiCa | 1-2 | 0.9671 | 0.9663 to 0.9680 |
|  | 1-3 | 0.9464 | 0.9450 to 0.9478 |  |  | 1-3 | 0.9861 | 0.9857 to 0.9865 |
|  | 2-3 | 0.9454 | 0.9439 to 0.9468 |  |  | 2-3 | 0.9835 | 0.9830 to 0.9839 |
|  |  |  |  |  | LarD | 1-2 | 0.9801 | 0.9796 to 0.9806 |
|  |  |  |  |  |  | 1-3 | 0.9780 | 0.9774 to 0.9786 |
|  |  |  |  |  |  | 2-3 | 0.9925 | 0.9923 to 0.9927 |

Abbreviations: Exp. Experiment; Conf. interval, Confidence interval; Biol. rep. Biological replicates. Arbos (Arbosana), LILe (long internodes length), Arbe (Arbequina), SILe (short internodes length), Pica (Picual active), Chi (Chiquitita), ChiCa (Chiquitita-like canopy), LarD (large trunk diameter), SmaD (small trunk diameter). *The r-value, at α=0.005 is 0.0133, and at α=0.05 is 0.0085. **The confidence interval is indicated (at 99%) for each pair of biological replicates. In all cases P-value was < 0.0001, being all correlations significant at alpha=0.05. .

**Table S4**. Accession numbers for the 2,252 candidate genes, and their correspondence with the reference transcriptome assembly ([Muñoz-Mérida et al., 2013](#_ENREF_62)).

| Contig Microarray | Unigene in reference transcriptome | NCBI  Accession Nº |
| --- | --- | --- |
| Contig_1004 | Unigene003465 | GABQ01079018 |
| Contig_10061 | Unigene000272 | GABQ01079297 |
| Contig_10084 | Unigene049038 | GABQ01053611 |
| Contig_10090 | Unigene003911 | GABQ01076351 |
| Contig_10119 | Unigene043340 | GABQ01070433 |
| Contig_10164 | Unigene041394 | GABQ01075100 |
| Contig_10213 | Unigene012540 | GABQ01064320 |
| Contig_10248 | Unigene093596 | GABQ01048456 |
| Contig_10295 | Unigene018664 | GABQ01044250 |
| Contig_10329 | Unigene041305 | GABQ01075279 |
| Contig_10387 | Unigene037910 | GABQ01079657 |
| Contig_10407 | Unigene004176 | GABQ01074891 |
| Contig_10439 | Unigene043657 | GABQ01069750 |
| Contig_10477 | Unigene001898 | GABQ01080514 |
| Contig_10500 | Unigene044431 | GABQ01067965 |
| Contig_10508 | Unigene036439 | GABQ01072773 |
| Contig_10518 | Unigene118589 | GABQ01073107 |
| Contig_10542 | Unigene016118 | GABQ01071677 |
| Contig_10548 | Unigene019102 | GABQ01052445 |
| Contig_10562 | Unigene012744 | GABQ01033428 |
| Contig_10591 | Unigene004047 | GABQ01075622 |
| Contig_10609 | Unigene061891 | GABQ01024801 |
| Contig_10644 | Unigene000281 | GABQ01069703 |
| Contig_10649 | Unigene042436 | GABQ01072575 |
| Contig_1065 | Unigene033303 | GABQ01060561 |
| Contig_1066 | Unigene002419 | GABQ01071744 |
| Contig_10663 | Unigene048953 | GABQ01055626 |
| Contig_10697 | Unigene043354 | GABQ01070397 |
| Contig_10713 | Unigene003492 | GABQ01078860 |
| Contig_10751 | Unigene027991 | GABQ01051632 |
| Contig_10772 | Unigene038728 | GABQ01070178 |
| Contig_10800 | Unigene037213 | GABQ01069632 |
| Contig_10843 | Unigene153531 | GABQ01055760 |
| Contig_10886 | Unigene035999 | GABQ01064964 |
| Contig_10907 | Unigene037862 | GABQ01080392 |
| Contig_10911 | Unigene123039 | GABQ01070583 |
| Contig_10967 | Unigene020926 | GABQ01077026 |
| Contig_10972 | Unigene021410 | GABQ01057635 |
| Contig_11002 | Unigene137848 | GABQ01072763 |
| Contig_11017 | Unigene032321 | GABQ01072847 |
| Contig_11070 | Unigene041314 | GABQ01075280 |
| Contig_11151 | Unigene003237 | GABQ01080289 |
| Contig_11156 | Unigene046476 | GABQ01063987 |
| Contig_11175 | Unigene047537 | GABQ01061085 |
| Contig_11187 | Unigene010490 | GABQ01066010 |
| Contig_11206 | Unigene110365 | GABQ01070824 |
| Contig_11225 | Unigene002852 | GABQ01064387 |
| Contig_11233 | Unigene053541 | GABQ01040369 |
| Contig_11273 | Unigene020444 | GABQ01073679 |
| Contig_11354 | Unigene037003 | GABQ01075190 |
| Contig_11391 | Unigene136881 |  |
| Contig_11446 | Unigene042176 | GABQ01073097 |
| Contig_11449 | Unigene004561 | GABQ01072126 |
| Contig_11484 | Unigene003494 | GABQ01078884 |
| Contig_11496 | Unigene097323 | GABQ01010360 |
| Contig_11629 | Unigene000645 | GABQ01080432 |
| Contig_11677 | Unigene044342 | GABQ01068379 |
| Contig_11699 | Unigene008740 | GABQ01066471 |
| Contig_1171 | Unigene010288 | GABQ01075771 |
| Contig_1185 | Unigene028434 | GABQ01073224 |
| Contig_11915 | Unigene044427 | GABQ01068249 |
| Contig_11919 | Unigene005355 | GABQ01069503 |
| Contig_11934 | Unigene020476 | GABQ01041145 |
| Contig_11955 | Unigene071685 | GABQ01069701 |
| Contig_11961 | Unigene008539 | GABQ01046773 |
| Contig_11988 | Unigene044183 | GABQ01068703 |
| Contig_12017 | Unigene041827 | GABQ01074098 |
| Contig_12039 | Unigene037635 | GABQ01051659 |
| Contig_12063 | Unigene001169 | GABQ01080935 |
| Contig_12125 | Unigene038746 | GABQ01070859 |
| Contig_12130 | Unigene078456 | GABQ01069872 |
| Contig_12131 | Unigene151296 |  |
| Contig_12134 | Unigene002479 | GABQ01070925 |
| Contig_12183 | Unigene000439 | GABQ01072562 |
| Contig_12197 | Unigene125541 | GABQ01001449 |
| Contig_12210 | Unigene000149 | GABQ01076523 |
| Contig_12255 | Unigene027214 | GABQ01063936 |
| Contig_12259 | Unigene002496 | GABQ01072202 |
| Contig_12290 | Unigene040767 | GABQ01076628 |
| Contig_12303 | Unigene003991 | GABQ01075998 |
| Contig_12323 | Unigene033512 | GABQ01068472 |
| Contig_12353 | Unigene033733 | GABQ01075795 |
| Contig_12371 | Unigene038814 | GABQ01053608 |
| Contig_12392 | Unigene099570 | GABQ01072428 |
| Contig_1245 | Unigene010947 | GABQ01031317 |
| Contig_12475 | Unigene026509 | GABQ01004244 |
| Contig_12481 | Unigene010109 | GABQ01055771 |
| Contig_1251 | Unigene006454 | GABQ01077025 |
| Contig_12518 | Unigene004606 | GABQ01071877 |
| Contig_12528 | Unigene122897 | GABQ01071475 |
| Contig_12541 | Unigene019899 | GABQ01074786 |
| Contig_12590 | Unigene009741 | GABQ01071239 |
| Contig_12626 | Unigene041339 | GABQ01075251 |
| Contig_12639 | Unigene032716 | GABQ01061388 |
| Contig_12643 | Unigene015835 | GABQ01058882 |
| Contig_12690 | Unigene041868 | GABQ01074010 |
| Contig_12692 | Unigene045516 | GABQ01066065 |
| Contig_12698 | Unigene031740 | GABQ01062137 |
| Contig_12728 | Unigene078476 | GABQ01036187 |
| Contig_12752 | Unigene013210 | GABQ01053567 |
| Contig_12766 | Unigene004220 | GABQ01074519 |
| Contig_12800 | Unigene000753 | GABQ01079214 |
| Contig_12813 | Unigene041290 | GABQ01075372 |
| Contig_12817 | Unigene001177 | GABQ01080769 |
| Contig_12840 | Unigene070816 | GABQ01067331 |
| Contig_12843 | Unigene034739 | GABQ01060107 |
| Contig_12870 | Unigene008861 | GABQ01075074 |
| Contig_12872 | Unigene005037 | GABQ01068268 |
| Contig_12902 | Unigene061430 | GABQ01022902 |
| Contig_12913 | Unigene143934 |  |
| Contig_12916 | Unigene145869 | GABQ01011287 |
| Contig_12979 | Unigene000013 |  |
| Contig_13053 | Unigene047745 | GABQ01060515 |
| Contig_13055 | Unigene029633 | GABQ01045111 |
| Contig_13070 | Unigene019579 | GABQ01064000 |
| Contig_13105 | Unigene034380 | GABQ01074818 |
| Contig_13157 | Unigene048029 | GABQ01059474 |
| Contig_13170 | Unigene026277 |  |
| Contig_13190 | Unigene004009 | GABQ01075837 |
| Contig_13289 | Unigene146426 | GABQ01008258 |
| Contig_13329 | Unigene002137 | GABQ01077438 |
| Contig_13547 | Unigene119430 | GABQ01063923 |
| Contig_13648 | Unigene029124 | GABQ01060611 |
| Contig_13749 | Unigene133810 | GABQ01015206 |
| Contig_13796 | Unigene032132 | GABQ01049991 |
| Contig_13831 | Unigene019724 | GABQ01053367 |
| Contig_13859 | Unigene002411 | GABQ01072880 |
| Contig_13907 | Unigene028356 | GABQ01041013 |
| Contig_1393 | Unigene002569 | GABQ01074221 |
| Contig_13940 | Unigene071702 | GABQ01056024 |
| Contig_13965 | Unigene003957 | GABQ01076165 |
| Contig_13976 | Unigene098045 | GABQ01032441 |
| Contig_14049 | Unigene117326 | GABQ01013025 |
| Contig_14082 | Unigene001459 | GABQ01080196 |
| Contig_14090 | Unigene148244 | GABQ01081014 |
| Contig_14109 | Unigene002271 | GABQ01076061 |
| Contig_1414 | Unigene042080 | GABQ01073542 |
| Contig_14174 | Unigene037116 | GABQ01080865 |
| Contig_14176 | Unigene021550 | GABQ01070914 |
| Contig_14205 | Unigene043292 | GABQ01070516 |
| Contig_14242 | Unigene027143 | GABQ01058540 |
| Contig_14306 | Unigene045922 | GABQ01065138 |
| Contig_14361 | Unigene005796 | GABQ01052535 |
| Contig_14364 | Unigene045862 | GABQ01065311 |
| Contig_14431 | Unigene159944 | GABQ01059293 |
| Contig_14533 | Unigene043418 | GABQ01070281 |
| Contig_14542 | Unigene049731 | GABQ01051052 |
| Contig_14602 | Unigene117280 | GABQ01072300 |
| Contig_14605 | Unigene000820 | GABQ01073109 |
| Contig_14649 | Unigene030550 | GABQ01067772 |
| Contig_14664 | Unigene039742 | GABQ01079839 |
| Contig_14665 | Unigene102175 | GABQ01080998 |
| Contig_14755 | Unigene072080 | GABQ01070523 |
| Contig_14800 | Unigene040383 | GABQ01077609 |
| Contig_14817 | Unigene041203 | GABQ01075556 |
| Contig_14819 | Unigene045117 | GABQ01066896 |
| Contig_14839 | Unigene040872 | GABQ01076367 |
| Contig_1484 | Unigene002290 | GABQ01072860 |
| Contig_1489 | Unigene001262 | GABQ01077348 |
| Contig_14907 | Unigene001041 | GABQ01077185 |
| Contig_14918 | Unigene002666 | GABQ01075396 |
| Contig_14935 | Unigene048137 | GABQ01059212 |
| Contig_15014 | Unigene001666 | GABQ01078624 |
| Contig_15042 | Unigene140512 | GABQ01071196 |
| Contig_15056 | Unigene004105 | GABQ01075293 |
| Contig_15096 | Unigene045571 | GABQ01065725 |
| Contig_1516 | Unigene046273 | GABQ01064443 |
| Contig_15239 | Unigene003952 | GABQ01076218 |
| Contig_1528 | Unigene042387 | GABQ01072627 |
| Contig_15293 | Unigene002496 | GABQ01072202 |
| Contig_15318 | Unigene000427 | GABQ01076508 |
| Contig_15333 | Unigene145510 |  |
| Contig_15337 | Unigene001476 | GABQ01080073 |
| Contig_15342 | Unigene055842 | GABQ01068359 |
| Contig_15380 | Unigene087163 | GABQ01080994 |
| Contig_15381 | Unigene005297 | GABQ01066506 |
| Contig_15472 | Unigene044654 | GABQ01067697 |
| Contig_15512 | Unigene047712 | GABQ01060572 |
| Contig_15531 | Unigene047376 | GABQ01061682 |
| Contig_15544 | Unigene047423 | GABQ01061518 |
| Contig_15551 | Unigene000803 | GABQ01074366 |
| Contig_15657 | Unigene004300 | GABQ01074116 |
| Contig_15739 | Unigene002670 | GABQ01071288 |
| Contig_15800 | Unigene058450 | GABQ01061806 |
| Contig_15816 | Unigene050653 | GABQ01043317 |
| Contig_15885 | Unigene130491 | GABQ01039938 |
| Contig_15961 | Unigene037595 | GABQ01072821 |
| Contig_15965 | Unigene038094 | GABQ01075476 |
| Contig_16088 | Unigene004503 | GABQ01072644 |
| Contig_16197 | Unigene020926 | GABQ01077026 |
| Contig_16341 | Unigene014084 | GABQ01054122 |
| Contig_16385 | Unigene001564 | GABQ01079404 |
| Contig_16431 | Unigene000083 | GABQ01080893 |
| Contig_165 | Unigene044542 | GABQ01067836 |
| Contig_16552 | Unigene133420 | GABQ01073379 |
| Contig_16707 | Unigene037717 | GABQ01059735 |
| Contig_16709 | Unigene037232 | GABQ01064965 |
| Contig_16738 | Unigene003023 | GABQ01052891 |
| Contig_16794 | Unigene135491 | GABQ01021937 |
| Contig_16820 | Unigene000305 | GABQ01077138 |
| Contig_16839 | Unigene042110 | GABQ01073444 |
| Contig_16884 | Unigene011112 | GABQ01058518 |
| Contig_16920 | Unigene009427 | GABQ01069304 |
| Contig_17017 | Unigene136338 |  |
| Contig_17028 | Unigene041649 | GABQ01080975 |
| Contig_17040 | Unigene032417 | GABQ01047092 |
| Contig_17051 | Unigene037237 | GABQ01078635 |
| Contig_17086 | Unigene022769 | GABQ01052930 |
| Contig_17106 | Unigene040446 | GABQ01077296 |
| Contig_17132 | Unigene027105 | GABQ01073585 |
| Contig_17145 | Unigene007167 | GABQ01047834 |
| Contig_17228 | Unigene024630 | GABQ01055197 |
| Contig_17232 | Unigene003911 | GABQ01076351 |
| Contig_17237 | Unigene038093 | GABQ01078061 |
| Contig_17243 | Unigene051223 | GABQ01036905 |
| Contig_1725 | Unigene037682 | GABQ01069692 |
| Contig_17252 | Unigene153585 | GABQ01027531 |
| Contig_17277 | Unigene026667 | GABQ01054255 |
| Contig_17290 | Unigene042074 | GABQ01073387 |
| Contig_1734 | Unigene020448 | GABQ01049427 |
| Contig_1737 | Unigene004262 | GABQ01074355 |
| Contig_17379 | Unigene021410 | GABQ01057635 |
| Contig_17404 | Unigene012150 | GABQ01022704 |
| Contig_17424 | Unigene160950 | GABQ01017109 |
| Contig_1750 | Unigene022645 | GABQ01048669 |
| Contig_17505 | Unigene036411 | GABQ01062524 |
| Contig_17516 | Unigene056779 | GABQ01065673 |
| Contig_17569 | Unigene020568 | GABQ01005158 |
| Contig_17582 | Unigene079408 | GABQ01047538 |
| Contig_17619 | Unigene044962 | GABQ01067166 |
| Contig_17648 | Unigene059234 | GABQ01052626 |
| Contig_17688 | Unigene038287 | GABQ01074487 |
| Contig_17734 | Unigene033276 | GABQ01055409 |
| Contig_17750 | Unigene117475 | GABQ01055658 |
| Contig_1779 | Unigene038931 | GABQ01064462 |
| Contig_17797 | Unigene041649 | GABQ01080975 |
| Contig_17843 | Unigene039761 | GABQ01079778 |
| Contig_17967 | Unigene077940 | GABQ01071640 |
| Contig_17974 | Unigene007874 | GABQ01062942 |
| Contig_18022 | Unigene026274 | GABQ01059460 |
| Contig_18032 | Unigene029557 | GABQ01071729 |
| Contig_18040 | Unigene099922 |  |
| Contig_18118 | Unigene130922 | GABQ01058798 |
| Contig_18196 | Unigene020069 | GABQ01043104 |
| Contig_18203 | Unigene089294 | GABQ01031558 |
| Contig_18296 | Unigene105184 | GABQ01069286 |
| Contig_1830 | Unigene042971 | GABQ01071183 |
| Contig_18323 | Unigene014931 | GABQ01055280 |
| Contig_18332 | Unigene002794 | GABQ01065918 |
| Contig_18359 | Unigene034588 | GABQ01065786 |
| Contig_18576 | Unigene027445 | GABQ01070636 |
| Contig_18582 | Unigene002918 | GABQ01060811 |
| Contig_18589 | Unigene028222 | GABQ01050253 |
| Contig_18677 | Unigene044627 | GABQ01067817 |
| Contig_18714 | Unigene022723 | GABQ01068490 |
| Contig_18723 | Unigene015071 | GABQ01067422 |
| Contig_1875 | Unigene001348 | GABQ01070214 |
| Contig_18785 | Unigene041969 | GABQ01073715 |
| Contig_18803 | Unigene004061 | GABQ01075508 |
| Contig_18821 | Unigene020160 | GABQ01050236 |
| Contig_18830 | Unigene037840 | GABQ01079276 |
| Contig_18849 | Unigene032923 | GABQ01064360 |
| Contig_18888 | Unigene157438 | GABQ01045597 |
| Contig_18911 | Unigene002759 | GABQ01072168 |
| Contig_18936 | Unigene151047 | GABQ01073157 |
| Contig_18937 | Unigene007010 | GABQ01034494 |
| Contig_18988 | Unigene139691 | GABQ01081012 |
| Contig_19031 | Unigene076092 | GABQ01032398 |
| Contig_19100 | Unigene044288 | GABQ01068373 |
| Contig_19149 | Unigene009733 | GABQ01018918 |
| Contig_19156 | Unigene052036 | GABQ01019076 |
| Contig_19159 | Unigene030894 | GABQ01042820 |
| Contig_19168 | Unigene043328 | GABQ01070476 |
| Contig_19172 | Unigene039479 | GABQ01047250 |
| Contig_19177 | Unigene040156 | GABQ01078287 |
| Contig_1918 | Unigene000373 | GABQ01079556 |
| Contig_19187 | Unigene001767 | GABQ01077979 |
| Contig_19263 | Unigene047752 | GABQ01060401 |
| Contig_19275 | Unigene014283 | GABQ01002312 |
| Contig_19281 | Unigene043751 | GABQ01069528 |
| Contig_19294 | Unigene131130 | GABQ01026856 |
| Contig_19405 | Unigene005229 | GABQ01070988 |
| Contig_19415 | Unigene003274 | GABQ01080096 |
| Contig_19446 | Unigene025942 | GABQ01038852 |
| Contig_19499 | Unigene012976 | GABQ01031780 |
| Contig_19500 | Unigene033426 | GABQ01027727 |
| Contig_19541 | Unigene044455 | GABQ01068156 |
| Contig_19562 | Unigene051219 | GABQ01036902 |
| Contig_19565 | Unigene042977 | GABQ01071274 |
| Contig_19569 | Unigene019050 |  |
| Contig_19628 | Unigene013076 |  |
| Contig_19630 | Unigene003427 | GABQ01079324 |
| Contig_19636 | Unigene003745 | GABQ01077471 |
| Contig_19694 | Unigene041253 | GABQ01075451 |
| Contig_19725 | Unigene012547 | GABQ01006691 |
| Contig_1973 | Unigene160344 | GABQ01039954 |
| Contig_19734 | Unigene012332 |  |
| Contig_19758 | Unigene051579 | GABQ01029935 |
| Contig_19774 | Unigene077596 | GABQ01010710 |
| Contig_19789 | Unigene005154 | GABQ01072107 |
| Contig_19797 | Unigene049646 | GABQ01051673 |
| Contig_19813 | Unigene041189 | GABQ01075610 |
| Contig_19836 | Unigene039544 | GABQ01029916 |
| Contig_19878 | Unigene005969 | GABQ01031288 |
| Contig_19904 | Unigene040571 | GABQ01076973 |
| Contig_19965 | Unigene036765 | GABQ01080151 |
| Contig_19987 | Unigene027205 | GABQ01036095 |
| Contig_19995 | Unigene004619 | GABQ01071754 |
| Contig_20007 | Unigene048048 | GABQ01059411 |
| Contig_20015 | Unigene048001 | GABQ01059060 |
| Contig_20050 | Unigene008377 | GABQ01017714 |
| Contig_20059 | Unigene046821 | GABQ01063147 |
| Contig_20075 | Unigene125768 | GABQ01035957 |
| Contig_20124 | Unigene044207 | GABQ01068687 |
| Contig_20125 | Unigene043942 | GABQ01069097 |
| Contig_20134 | Unigene003589 | GABQ01078447 |
| Contig_20178 | Unigene005890 |  |
| Contig_20188 | Unigene021705 | GABQ01034242 |
| Contig_20193 | Unigene023652 | GABQ01044550 |
| Contig_20196 | Unigene045117 | GABQ01066896 |
| Contig_20204 | Unigene041317 | GABQ01075298 |
| Contig_20259 | Unigene012091 | GABQ01035465 |
| Contig_20272 | Unigene050187 | GABQ01047386 |
| Contig_20278 | Unigene003427 | GABQ01079324 |
| Contig_20329 | Unigene047507 | GABQ01061259 |
| Contig_20393 | Unigene051315 | GABQ01034950 |
| Contig_20410 | Unigene044819 | GABQ01067384 |
| Contig_20437 | Unigene047603 | GABQ01060985 |
| Contig_20459 | Unigene037246 | GABQ01080932 |
| Contig_20521 | Unigene000280 | GABQ01070344 |
| Contig_20526 | Unigene118754 | GABQ01029645 |
| Contig_20607 | Unigene003427 | GABQ01079324 |
| Contig_20640 | Unigene001969 | GABQ01076116 |
| Contig_20671 | Unigene049333 | GABQ01053619 |
| Contig_20690 | Unigene002288 | GABQ01074276 |
| Contig_20702 | Unigene027219 | GABQ01032716 |
| Contig_20728 | Unigene010421 | GABQ01059707 |
| Contig_20803 | Unigene043418 | GABQ01070281 |
| Contig_20818 | Unigene049786 | GABQ01050559 |
| Contig_20822 | Unigene019411 | GABQ01038834 |
| Contig_20866 | Unigene023388 | GABQ01047472 |
| Contig_20882 | Unigene001988 | GABQ01075660 |
| Contig_20889 | Unigene090768 | GABQ01009566 |
| Contig_20898 | Unigene049373 | GABQ01053515 |
| Contig_20927 | Unigene000483 | GABQ01080055 |
| Contig_20928 | Unigene039434 | GABQ01042989 |
| Contig_2099 | Unigene043677 | GABQ01069754 |
| Contig_20991 | Unigene047362 | GABQ01061507 |
| Contig_20998 | Unigene000112 | GABQ01068907 |
| Contig_2106 | Unigene012118 | GABQ01062812 |
| Contig_21090 | Unigene044055 | GABQ01068949 |
| Contig_21096 | Unigene001160 | GABQ01068046 |
| Contig_21159 | Unigene003133 | GABQ01080918 |
| Contig_21181 | Unigene050373 | GABQ01045958 |
| Contig_21233 | Unigene003967 | GABQ01076131 |
| Contig_21267 | Unigene006631 | GABQ01028130 |
| Contig_21386 | Unigene130516 | GABQ01008219 |
| Contig_21444 | Unigene005856 | GABQ01010935 |
| Contig_21465 | Unigene066764 | GABQ01022293 |
| Contig_21485 | Unigene138005 | GABQ01018213 |
| Contig_21494 | Unigene015508 | GABQ01011829 |
| Contig_21495 | Unigene052095 | GABQ01017304 |
| Contig_21559 | Unigene047666 | GABQ01060805 |
| Contig_21568 | Unigene154933 |  |
| Contig_21581 | Unigene001754 | GABQ01077987 |
| Contig_21589 | Unigene004035 | GABQ01075692 |
| Contig_21594 | Unigene016012 | GABQ01038003 |
| Contig_21603 | Unigene117936 | GABQ01016337 |
| Contig_21607 | Unigene149748 | GABQ01019448 |
| Contig_21609 | Unigene004024 | GABQ01075804 |
| Contig_21680 | Unigene043464 | GABQ01070187 |
| Contig_21791 | Unigene025932 | GABQ01058538 |
| Contig_21825 | Unigene051934 | GABQ01021569 |
| Contig_21837 | Unigene005501 | GABQ01060085 |
| Contig_21841 | Unigene001960 | GABQ01076802 |
| Contig_21861 | Unigene044593 | GABQ01067754 |
| Contig_21871 | Unigene040379 |  |
| Contig_21909 | Unigene028484 | GABQ01049314 |
| Contig_21923 | Unigene000010 |  |
| Contig_21949 | Unigene005342 | GABQ01064223 |
| Contig_21960 | Unigene051641 | GABQ01027171 |
| Contig_22059 | Unigene006051 | GABQ01063651 |
| Contig_22061 | Unigene046291 | GABQ01064071 |
| Contig_22069 | Unigene001643 | GABQ01078941 |
| Contig_22078 | Unigene020923 | GABQ01018384 |
| Contig_22108 | Unigene004861 | GABQ01069840 |
| Contig_22118 | Unigene009183 | GABQ01014800 |
| Contig_22139 | Unigene001003 | GABQ01078282 |
| Contig_22170 | Unigene008364 | GABQ01035445 |
| Contig_22179 | Unigene043377 | GABQ01070376 |
| Contig_22185 | Unigene003446 | GABQ01079160 |
| Contig_22190 | Unigene040211 | GABQ01078133 |
| Contig_22191 | Unigene038266 | GABQ01075171 |
| Contig_22193 | Unigene000124 | GABQ01080859 |
| Contig_22237 | Unigene008187 | GABQ01014261 |
| Contig_22292 | Unigene015810 |  |
| Contig_22364 | Unigene013548 | GABQ01051733 |
| Contig_22365 | Unigene043025 | GABQ01071040 |
| Contig_22366 | Unigene000645 | GABQ01080432 |
| Contig_22371 | Unigene052550 | GABQ01000074 |
| Contig_22382 | Unigene047189 | GABQ01062150 |
| Contig_224 | Unigene044660 | GABQ01067731 |
| Contig_22402 | Unigene030716 | GABQ01058325 |
| Contig_2242 | Unigene037788 | GABQ01075578 |
| Contig_22427 | Unigene005386 | GABQ01063290 |
| Contig_22439 | Unigene015899 | GABQ01026475 |
| Contig_22474 | Unigene010706 | GABQ01014275 |
| Contig_22477 | Unigene043243 | GABQ01070677 |
| Contig_22504 | Unigene002866 | GABQ01062995 |
| Contig_22555 | Unigene033203 | GABQ01061993 |
| Contig_22637 | Unigene050717 | GABQ01042853 |
| Contig_22684 | Unigene038467 | GABQ01076747 |
| Contig_22697 | Unigene022376 | GABQ01063964 |
| Contig_22700 | Unigene036124 | GABQ01052604 |
| Contig_22701 | Unigene005318 | GABQ01064598 |
| Contig_22707 | Unigene013307 | GABQ01058656 |
| Contig_22746 | Unigene048230 | GABQ01058856 |
| Contig_22749 | Unigene000869 | GABQ01059962 |
| Contig_22760 | Unigene044515 | GABQ01067937 |
| Contig_22768 | Unigene040994 | GABQ01076046 |
| Contig_22771 | Unigene021358 | GABQ01027069 |
| Contig_22776 | Unigene043072 | GABQ01071084 |
| Contig_22784 | Unigene038387 | GABQ01072692 |
| Contig_22803 | Unigene040112 | GABQ01078474 |
| Contig_22819 | Unigene017260 | GABQ01005149 |
| Contig_22821 | Unigene051978 | GABQ01020320 |
| Contig_22856 | Unigene000912 | GABQ01080314 |
| Contig_22925 | Unigene010157 | GABQ01028151 |
| Contig_22971 | Unigene022376 | GABQ01063964 |
| Contig_22978 | Unigene039738 | GABQ01079857 |
| Contig_2301 | Unigene026159 | GABQ01069567 |
| Contig_23066 | Unigene047800 | GABQ01060342 |
| Contig_23085 | Unigene002800 | GABQ01067298 |
| Contig_23111 | Unigene001924 | GABQ01076533 |
| Contig_23128 | Unigene002270 | GABQ01073021 |
| Contig_23130 | Unigene051427 | GABQ01032758 |
| Contig_23134 | Unigene037630 | GABQ01058769 |
| Contig_23136 | Unigene042373 | GABQ01072665 |
| Contig_23157 | Unigene006685 | GABQ01057464 |
| Contig_23206 | Unigene039411 | GABQ01065754 |
| Contig_23226 | Unigene048742 | GABQ01056793 |
| Contig_23256 | Unigene004043 | GABQ01075604 |
| Contig_23284 | Unigene003586 | GABQ01078451 |
| Contig_23293 | Unigene046476 | GABQ01063987 |
| Contig_23332 | Unigene069108 | GABQ01016776 |
| Contig_23336 | Unigene049587 | GABQ01052037 |
| Contig_23381 | Unigene012576 | GABQ01042642 |
| Contig_2343 | Unigene004301 | GABQ01074091 |
| Contig_23527 | Unigene060596 | GABQ01006169 |
| Contig_23584 | Unigene007431 | GABQ01004846 |
| Contig_23601 | Unigene084871 | GABQ01012499 |
| Contig_23603 | Unigene041383 | GABQ01075123 |
| Contig_23604 | Unigene041917 | GABQ01073869 |
| Contig_23606 | Unigene041451 | GABQ01074975 |
| Contig_2361 | Unigene012540 | GABQ01064320 |
| Contig_2369 | Unigene018670 | GABQ01071535 |
| Contig_23714 | Unigene012360 | GABQ01003361 |
| Contig_23726 | Unigene002039 | GABQ01076153 |
| Contig_23735 | Unigene051621 | GABQ01028846 |
| Contig_23745 | Unigene005736 | GABQ01074705 |
| Contig_23768 | Unigene048964 | GABQ01055324 |
| Contig_23773 | Unigene046419 | GABQ01064165 |
| Contig_23845 | Unigene039894 | GABQ01079262 |
| Contig_23851 | Unigene037211 | GABQ01074749 |
| Contig_23872 | Unigene003102 | GABQ01035733 |
| Contig_23943 | Unigene033774 | GABQ01068715 |
| Contig_23961 | Unigene013103 | GABQ01034834 |
| Contig_23977 | Unigene022156 | GABQ01009413 |
| Contig_23998 | Unigene009755 | GABQ01023306 |
| Contig_2405 | Unigene004865 | GABQ01069764 |
| Contig_24063 | Unigene006682 | GABQ01038178 |
| Contig_2408 | Unigene037017 | GABQ01065355 |
| Contig_24084 | Unigene025380 | GABQ01023406 |
| Contig_24106 | Unigene044805 | GABQ01067457 |
| Contig_24110 | Unigene036713 | GABQ01070777 |
| Contig_24116 | Unigene008395 | GABQ01046158 |
| Contig_24152 | Unigene042255 | GABQ01073136 |
| Contig_24193 | Unigene015680 |  |
| Contig_24243 | Unigene013983 | GABQ01052325 |
| Contig_24265 | Unigene004960 | GABQ01068955 |
| Contig_24267 | Unigene015544 | GABQ01029280 |
| Contig_24318 | Unigene021588 |  |
| Contig_24322 | Unigene036838 | GABQ01075036 |
| Contig_24326 | Unigene093086 |  |
| Contig_24331 | Unigene037470 | GABQ01075784 |
| Contig_24374 | Unigene036308 | GABQ01038692 |
| Contig_24396 | Unigene047158 | GABQ01062315 |
| Contig_24416 | Unigene006078 | GABQ01052426 |
| Contig_24460 | Unigene050176 | GABQ01046848 |
| Contig_24477 | Unigene002615 | GABQ01068185 |
| Contig_24489 | Unigene016644 | GABQ01001026 |
| Contig_24503 | Unigene038386 | GABQ01074858 |
| Contig_24513 | Unigene039343 | GABQ01064299 |
| Contig_24534 | Unigene049269 | GABQ01053819 |
| Contig_24554 | Unigene051951 | GABQ01020310 |
| Contig_24585 | Unigene003735 | GABQ01077510 |
| Contig_24656 | Unigene048612 | GABQ01057279 |
| Contig_24753 | Unigene002567 | GABQ01072305 |
| Contig_24755 | Unigene037692 | GABQ01066541 |
| Contig_24805 | Unigene005702 | GABQ01044502 |
| Contig_24814 | Unigene039171 | GABQ01062571 |
| Contig_24842 | Unigene034216 | GABQ01055517 |
| Contig_24857 | Unigene047384 | GABQ01061511 |
| Contig_2493 | Unigene045626 | GABQ01065762 |
| Contig_24948 | Unigene013228 |  |
| Contig_24993 | Unigene041710 | GABQ01074393 |
| Contig_25 | Unigene035094 | GABQ01055318 |
| Contig_25003 | Unigene002371 | GABQ01072183 |
| Contig_25080 | Unigene018373 | GABQ01061431 |
| Contig_25082 | Unigene001398 |  |
| Contig_25111 | Unigene111917 | GABQ01021824 |
| Contig_25123 | Unigene000427 | GABQ01076508 |
| Contig_25197 | Unigene034656 | GABQ01052600 |
| Contig_25212 | Unigene025375 | GABQ01024663 |
| Contig_25231 | Unigene047887 | GABQ01060069 |
| Contig_25236 | Unigene004312 | GABQ01073992 |
| Contig_25291 | Unigene050064 | GABQ01048436 |
| Contig_25347 | Unigene004579 | GABQ01071778 |
| Contig_25355 | Unigene002270 | GABQ01073021 |
| Contig_25373 | Unigene015956 | GABQ01020190 |
| Contig_25375 | Unigene016367 | GABQ01036042 |
| Contig_25383 | Unigene005287 | GABQ01065115 |
| Contig_25431 | Unigene039580 | GABQ01080845 |
| Contig_25513 | Unigene051710 | GABQ01025989 |
| Contig_25542 | Unigene040927 | GABQ01076180 |
| Contig_25584 | Unigene043447 | GABQ01070222 |
| Contig_25616 | Unigene042134 | GABQ01073418 |
| Contig_25633 | Unigene050457 | GABQ01045145 |
| Contig_25682 | Unigene005800 | GABQ01026965 |
| Contig_25700 | Unigene015589 | GABQ01022720 |
| Contig_2575 | Unigene043116 | GABQ01070836 |
| Contig_25771 | Unigene005077 |  |
| Contig_25797 | Unigene037247 | GABQ01079704 |
| Contig_25818 | Unigene047984 | GABQ01059756 |
| Contig_25903 | Unigene000402 | GABQ01080378 |
| Contig_25910 | Unigene019899 | GABQ01074786 |
| Contig_25920 | Unigene126282 | GABQ01033725 |
| Contig_25953 | Unigene044983 | GABQ01067008 |
| Contig_25967 | Unigene051789 | GABQ01024735 |
| Contig_25972 | Unigene037310 | GABQ01077464 |
| Contig_25995 | Unigene045992 | GABQ01065037 |
| Contig_25998 | Unigene003960 | GABQ01076103 |
| Contig_26 | Unigene030889 | GABQ01046673 |
| Contig_2601 | Unigene000439 | GABQ01072562 |
| Contig_26015 | Unigene002726 | GABQ01066527 |
| Contig_26024 | Unigene047167 | GABQ01062106 |
| Contig_26027 | Unigene027431 | GABQ01063508 |
| Contig_26083 | Unigene005120 | GABQ01067332 |
| Contig_26188 | Unigene006258 | GABQ01056735 |
| Contig_26193 | Unigene025316 | GABQ01021478 |
| Contig_26231 | Unigene048496 | GABQ01057432 |
| Contig_26232 | Unigene023274 | GABQ01059981 |
| Contig_26233 | Unigene032296 | GABQ01025353 |
| Contig_26234 | Unigene036062 | GABQ01040030 |
| Contig_26237 | Unigene023850 | GABQ01048141 |
| Contig_26248 | Unigene013811 | GABQ01044099 |
| Contig_26349 | Unigene001834 | GABQ01078767 |
| Contig_26404 | Unigene039846 | GABQ01079410 |
| Contig_26490 | Unigene041455 | GABQ01074976 |
| Contig_26506 | Unigene011986 | GABQ01034196 |
| Contig_2652 | Unigene041653 | GABQ01074491 |
| Contig_26559 | Unigene032213 | GABQ01034592 |
| Contig_26599 | Unigene000156 | GABQ01080460 |
| Contig_26605 | Unigene052071 | GABQ01016689 |
| Contig_26610 | Unigene072757 |  |
| Contig_26702 | Unigene005773 | GABQ01057698 |
| Contig_26729 | Unigene043534 | GABQ01069849 |
| Contig_26746 | Unigene000411 | GABQ01077948 |
| Contig_26753 | Unigene041439 | GABQ01075008 |
| Contig_26795 | Unigene051964 | GABQ01020930 |
| Contig_26852 | Unigene047737 | GABQ01060512 |
| Contig_26853 | Unigene047737 | GABQ01060512 |
| Contig_26864 | Unigene017867 | GABQ01080517 |
| Contig_2688 | Unigene028750 | GABQ01049069 |
| Contig_26881 | Unigene000028 | GABQ01080327 |
| Contig_26895 | Unigene027126 | GABQ01040330 |
| Contig_26897 | Unigene030991 | GABQ01036112 |
| Contig_26976 | Unigene016310 | GABQ01022121 |
| Contig_27034 | Unigene016278 | GABQ01031801 |
| Contig_27035 | Unigene041077 | GABQ01075866 |
| Contig_27039 | Unigene003599 | GABQ01078381 |
| Contig_27076 | Unigene039017 | GABQ01066135 |
| Contig_27083 | Unigene040353 | GABQ01077714 |
| Contig_27105 | Unigene002607 | GABQ01068819 |
| Contig_27266 | Unigene045873 | GABQ01065318 |
| Contig_27286 | Unigene052187 | GABQ01015467 |
| Contig_27318 | Unigene003356 | GABQ01079690 |
| Contig_27326 | Unigene027714 | GABQ01060230 |
| Contig_27362 | Unigene045396 | GABQ01066218 |
| Contig_27435 | Unigene014735 | GABQ01079981 |
| Contig_27441 | Unigene041927 | GABQ01073923 |
| Contig_27486 | Unigene044614 | GABQ01067867 |
| Contig_27527 | Unigene040447 | GABQ01077452 |
| Contig_27552 | Unigene016106 | GABQ01009392 |
| Contig_27559 | Unigene045985 | GABQ01064971 |
| Contig_27580 | Unigene048861 | GABQ01056098 |
| Contig_27587 | Unigene029759 | GABQ01025953 |
| Contig_27604 | Unigene051971 | GABQ01020313 |
| Contig_27660 | Unigene001926 | GABQ01076193 |
| Contig_27673 | Unigene084369 | GABQ01075799 |
| Contig_27701 | Unigene050875 | GABQ01041190 |
| Contig_27720 | Unigene020017 | GABQ01039439 |
| Contig_27726 | Unigene017474 | GABQ01052785 |
| Contig_27737 | Unigene042913 | GABQ01071386 |
| Contig_27808 | Unigene047013 | GABQ01062435 |
| Contig_27810 | Unigene017438 | GABQ01070609 |
| Contig_27841 | Unigene001861 |  |
| Contig_27856 | Unigene039747 | GABQ01079822 |
| Contig_27865 | Unigene039968 | GABQ01078898 |
| Contig_27889 | Unigene052297 | GABQ01011471 |
| Contig_27914 | Unigene031429 | GABQ01047892 |
| Contig_27940 | Unigene008748 |  |
| Contig_27967 | Unigene001828 | GABQ01076992 |
| Contig_28000 | Unigene001143 | GABQ01078300 |
| Contig_28047 | Unigene040489 | GABQ01077239 |
| Contig_28075 | Unigene012938 | GABQ01031777 |
| Contig_28109 | Unigene016743 | GABQ01051001 |
| Contig_28137 | Unigene039894 | GABQ01079262 |
| Contig_2814 | Unigene002369 | GABQ01071355 |
| Contig_28152 | Unigene017992 | GABQ01036049 |
| Contig_28177 | Unigene042059 | GABQ01073604 |
| Contig_28220 | Unigene044240 | GABQ01068599 |
| Contig_2824 | Unigene010917 | GABQ01071944 |
| Contig_28256 | Unigene024519 | GABQ01047745 |
| Contig_28277 | Unigene001774 | GABQ01080178 |
| Contig_28281 | Unigene010182 | GABQ01023955 |
| Contig_28339 | Unigene050153 | GABQ01047645 |
| Contig_28345 | Unigene047163 | GABQ01062201 |
| Contig_28367 | Unigene042935 | GABQ01071230 |
| Contig_28566 | Unigene031168 | GABQ01057808 |
| Contig_28589 | Unigene025649 | GABQ01065416 |
| Contig_2859 | Unigene135199 | GABQ01036263 |
| Contig_28679 | Unigene040261 | GABQ01077978 |
| Contig_28684 | Unigene099089 | GABQ01012967 |
| Contig_28742 | Unigene031200 | GABQ01029895 |
| Contig_28772 | Unigene001473 | GABQ01080425 |
| Contig_28789 | Unigene000199 | GABQ01079234 |
| Contig_28812 | Unigene003368 | GABQ01079621 |
| Contig_28871 | Unigene040243 | GABQ01078007 |
| Contig_28881 | Unigene006482 | GABQ01047832 |
| Contig_28896 | Unigene049897 | GABQ01049888 |
| Contig_28935 | Unigene005684 | GABQ01061980 |
| Contig_28960 | Unigene040538 | GABQ01077217 |
| Contig_28979 | Unigene035746 | GABQ01012388 |
| Contig_2898 | Unigene039905 | GABQ01079224 |
| Contig_28993 | Unigene001853 | GABQ01077962 |
| Contig_29011 | Unigene009152 | GABQ01013733 |
| Contig_29023 | Unigene044945 | GABQ01067162 |
| Contig_29036 | Unigene039909 | GABQ01079170 |
| Contig_29050 | Unigene160793 | GABQ01011781 |
| Contig_29083 | Unigene117988 | GABQ01013564 |
| Contig_29084 | Unigene000201 | GABQ01066995 |
| Contig_29101 | Unigene049510 | GABQ01052622 |
| Contig_29122 | Unigene004193 | GABQ01074739 |
| Contig_29266 | Unigene005678 | GABQ01047444 |
| Contig_29276 | Unigene046968 | GABQ01062791 |
| Contig_29292 | Unigene021490 | GABQ01046628 |
| Contig_29295 | Unigene000044 | GABQ01076881 |
| Contig_29300 | Unigene041858 | GABQ01074074 |
| Contig_29372 | Unigene050833 | GABQ01041646 |
| Contig_29414 | Unigene047248 | GABQ01062006 |
| Contig_29460 | Unigene093499 | GABQ01004994 |
| Contig_29497 | Unigene012008 | GABQ01050751 |
| Contig_29502 | Unigene006510 | GABQ01020114 |
| Contig_29627 | Unigene044829 | GABQ01067387 |
| Contig_29636 | Unigene012306 | GABQ01006088 |
| Contig_29684 | Unigene003688 | GABQ01078558 |
| Contig_29748 | Unigene045802 | GABQ01065169 |
| Contig_29788 | Unigene049569 | GABQ01051913 |
| Contig_29790 | Unigene015700 | GABQ01032652 |
| Contig_29887 | Unigene044087 | GABQ01068903 |
| Contig_29915 | Unigene040635 | GABQ01076982 |
| Contig_29942 | Unigene052442 | GABQ01005821 |
| Contig_29970 | Unigene009831 | GABQ01044662 |
| Contig_30007 | Unigene029512 | GABQ01057888 |
| Contig_30045 | Unigene034168 | GABQ01006136 |
| Contig_30060 | Unigene046811 | GABQ01062911 |
| Contig_30089 | Unigene038462 | GABQ01073242 |
| Contig_30117 | Unigene039521 | GABQ01038271 |
| Contig_3012 | Unigene075735 |  |
| Contig_30127 | Unigene044911 | GABQ01066906 |
| Contig_30141 | Unigene006690 | GABQ01036313 |
| Contig_30145 | Unigene023366 | GABQ01030860 |
| Contig_30151 | Unigene001952 | GABQ01078177 |
| Contig_30152 | Unigene029781 | GABQ01044280 |
| Contig_30163 | Unigene033759 | GABQ01067773 |
| Contig_30175 | Unigene005561 | GABQ01059100 |
| Contig_30194 | Unigene001570 | GABQ01079244 |
| Contig_30252 | Unigene071724 | GABQ01028927 |
| Contig_30254 | Unigene049149 | GABQ01054950 |
| Contig_30282 | Unigene039264 | GABQ01054922 |
| Contig_30297 | Unigene012455 | GABQ01047323 |
| Contig_30306 | Unigene038287 | GABQ01074487 |
| Contig_30331 | Unigene022266 |  |
| Contig_30367 | Unigene030751 | GABQ01042555 |
| Contig_30374 | Unigene114465 | GABQ01075055 |
| Contig_30468 | Unigene014580 |  |
| Contig_30489 | Unigene041673 | GABQ01074319 |
| Contig_30493 | Unigene046264 | GABQ01064509 |
| Contig_30503 | Unigene011629 | GABQ01032193 |
| Contig_30529 | Unigene002134 | GABQ01074887 |
| Contig_30531 | Unigene051606 | GABQ01029414 |
| Contig_30540 | Unigene044680 | GABQ01067700 |
| Contig_30582 | Unigene049398 | GABQ01053314 |
| Contig_30587 | Unigene021629 | GABQ01057939 |
| Contig_30610 | Unigene157258 | GABQ01006052 |
| Contig_30623 | Unigene011239 | GABQ01018926 |
| Contig_30624 | Unigene050777 | GABQ01042295 |
| Contig_30627 | Unigene017476 | GABQ01040147 |
| Contig_30669 | Unigene051338 | GABQ01034964 |
| Contig_30696 | Unigene001968 | GABQ01076246 |
| Contig_30713 | Unigene029846 | GABQ01028269 |
| Contig_30737 | Unigene011620 | GABQ01054007 |
| Contig_30766 | Unigene043659 | GABQ01069812 |
| Contig_30788 | Unigene001110 | GABQ01062890 |
| Contig_30789 | Unigene044179 | GABQ01068565 |
| Contig_30799 | Unigene011623 | GABQ01068308 |
| Contig_30824 | Unigene051699 | GABQ01027196 |
| Contig_30897 | Unigene041518 | GABQ01074777 |
| Contig_30951 | Unigene003578 | GABQ01078459 |
| Contig_30976 | Unigene022184 | GABQ01072147 |
| Contig_30991 | Unigene100895 | GABQ01023037 |
| Contig_31035 | Unigene041392 | GABQ01075098 |
| Contig_31049 | Unigene021905 | GABQ01074254 |
| Contig_31096 | Unigene001277 | GABQ01076524 |
| Contig_31119 | Unigene041394 | GABQ01075100 |
| Contig_31167 | Unigene047081 | GABQ01062442 |
| Contig_31176 | Unigene042537 | GABQ01072369 |
| Contig_31196 | Unigene020326 | GABQ01051006 |
| Contig_31221 | Unigene026152 | GABQ01033493 |
| Contig_31239 | Unigene003625 | GABQ01078240 |
| Contig_31336 | Unigene052381 | GABQ01008740 |
| Contig_31367 | Unigene033689 |  |
| Contig_3139 | Unigene018045 | GABQ01072531 |
| Contig_31443 | Unigene048761 | GABQ01056532 |
| Contig_31446 | Unigene004387 | GABQ01073455 |
| Contig_31457 | Unigene052007 | GABQ01019678 |
| Contig_31462 | Unigene024133 | GABQ01032699 |
| Contig_3153 | Unigene000281 | GABQ01069703 |
| Contig_31549 | Unigene051222 | GABQ01036904 |
| Contig_31608 | Unigene003994 | GABQ01075999 |
| Contig_31719 | Unigene043960 | GABQ01069136 |
| Contig_31740 | Unigene050491 | GABQ01044871 |
| Contig_31759 | Unigene032347 | GABQ01060390 |
| Contig_31806 | Unigene002332 | GABQ01071887 |
| Contig_31807 | Unigene045243 | GABQ01066575 |
| Contig_3187 | Unigene039877 | GABQ01079322 |
| Contig_31885 | Unigene002212 | GABQ01079226 |
| Contig_31991 | Unigene042208 | GABQ01073211 |
| Contig_32040 | Unigene050236 | GABQ01047001 |
| Contig_32060 | Unigene001882 | GABQ01078823 |
| Contig_32087 | Unigene025047 | GABQ01064262 |
| Contig_32157 | Unigene022671 | GABQ01057107 |
| Contig_32195 | Unigene046630 | GABQ01063642 |
| Contig_32205 | Unigene002251 | GABQ01073932 |
| Contig_32208 | Unigene039462 | GABQ01038695 |
| Contig_32309 | Unigene046059 | GABQ01064819 |
| Contig_32357 | Unigene041387 | GABQ01075097 |
| Contig_32373 | Unigene050744 | GABQ01042586 |
| Contig_32428 | Unigene042748 | GABQ01071762 |
| Contig_32433 | Unigene002169 | GABQ01075423 |
| Contig_32445 | Unigene028484 | GABQ01049314 |
| Contig_32458 | Unigene051351 | GABQ01034632 |
| Contig_32466 | Unigene033000 | GABQ01029378 |
| Contig_32476 | Unigene005624 | GABQ01053112 |
| Contig_32498 | Unigene038281 | GABQ01074509 |
| Contig_32499 | Unigene018509 | GABQ01036597 |
| Contig_32526 | Unigene039318 | GABQ01060564 |
| Contig_32550 | Unigene004178 | GABQ01074868 |
| Contig_32559 | Unigene026270 | GABQ01048810 |
| Contig_32562 | Unigene152264 | GABQ01044495 |
| Contig_32608 | Unigene008143 | GABQ01029785 |
| Contig_32639 | Unigene042074 | GABQ01073387 |
| Contig_32641 | Unigene022237 | GABQ01073897 |
| Contig_3265 | Unigene038093 | GABQ01078061 |
| Contig_32666 | Unigene045778 | GABQ01065451 |
| Contig_32671 | Unigene039690 | GABQ01080111 |
| Contig_32700 | Unigene007918 | GABQ01035439 |
| Contig_32712 | Unigene043572 | GABQ01069923 |
| Contig_32761 | Unigene037157 | GABQ01077807 |
| Contig_32770 | Unigene003126 |  |
| Contig_32787 | Unigene005350 | GABQ01064056 |
| Contig_32820 | Unigene050736 | GABQ01042582 |
| Contig_32866 | Unigene115355 | GABQ01013556 |
| Contig_32900 | Unigene043675 | GABQ01069753 |
| Contig_32917 | Unigene002895 | GABQ01067597 |
| Contig_32935 | Unigene039632 | GABQ01080457 |
| Contig_32994 | Unigene007035 | GABQ01013188 |
| Contig_33003 | Unigene001398 |  |
| Contig_33008 | Unigene004706 | GABQ01071091 |
| Contig_33056 | Unigene021051 | GABQ01065124 |
| Contig_33068 | Unigene001057 | GABQ01076800 |
| Contig_33088 | Unigene043624 | GABQ01069805 |
| Contig_33114 | Unigene016753 | GABQ01073415 |
| Contig_33158 | Unigene017770 | GABQ01043097 |
| Contig_33168 | Unigene043868 | GABQ01069277 |
| Contig_33182 | Unigene051178 | GABQ01037411 |
| Contig_33211 | Unigene029993 | GABQ01029362 |
| Contig_33217 | Unigene035090 | GABQ01052715 |
| Contig_33267 | Unigene000304 | GABQ01080172 |
| Contig_33322 | Unigene003776 | GABQ01077294 |
| Contig_33347 | Unigene026386 | GABQ01064025 |
| Contig_33379 | Unigene050514 | GABQ01044875 |
| Contig_33382 | Unigene051794 | GABQ01024740 |
| Contig_33384 | Unigene016244 | GABQ01052207 |
| Contig_33409 | Unigene001751 | GABQ01077923 |
| Contig_33420 | Unigene042222 | GABQ01073177 |
| Contig_33499 | Unigene034826 | GABQ01043441 |
| Contig_33512 | Unigene003132 | GABQ01080920 |
| Contig_33536 | Unigene010325 | GABQ01060363 |
| Contig_3355 | Unigene042408 | GABQ01072715 |
| Contig_33585 | Unigene043823 | GABQ01069148 |
| Contig_33640 | Unigene039347 | GABQ01062295 |
| Contig_33641 | Unigene146870 | GABQ01021303 |
| Contig_33662 | Unigene024574 | GABQ01047606 |
| Contig_33709 | Unigene039603 | GABQ01080644 |
| Contig_33711 | Unigene017094 | GABQ01042070 |
| Contig_33720 | Unigene049999 | GABQ01047907 |
| Contig_33749 | Unigene041563 | GABQ01074626 |
| Contig_33753 | Unigene013384 | GABQ01046446 |
| Contig_33760 | Unigene001512 | GABQ01080737 |
| Contig_33839 | Unigene063550 | GABQ01004635 |
| Contig_33877 | Unigene007815 | GABQ01037975 |
| Contig_33911 | Unigene038249 | GABQ01075181 |
| Contig_3392 | Unigene013787 | GABQ01078967 |
| Contig_33925 | Unigene042658 | GABQ01072099 |
| Contig_33932 | Unigene004303 | GABQ01074093 |
| Contig_33940 | Unigene092274 | GABQ01021098 |
| Contig_33958 | Unigene030567 | GABQ01011878 |
| Contig_33969 | Unigene049692 | GABQ01051415 |
| Contig_33983 | Unigene041890 | GABQ01073941 |
| Contig_33993 | Unigene037069 | GABQ01071144 |
| Contig_34013 | Unigene043821 | GABQ01069452 |
| Contig_34038 | Unigene039777 | GABQ01079697 |
| Contig_34044 | Unigene003241 | GABQ01080279 |
| Contig_34050 | Unigene014323 | GABQ01062458 |
| Contig_34080 | Unigene046523 | GABQ01063879 |
| Contig_34089 | Unigene039693 | GABQ01080046 |
| Contig_34090 | Unigene082313 | GABQ01065342 |
| Contig_34138 | Unigene004109 | GABQ01075254 |
| Contig_34153 | Unigene098138 | GABQ01029571 |
| Contig_34161 | Unigene027501 | GABQ01039063 |
| Contig_34225 | Unigene050671 | GABQ01042844 |
| Contig_34263 | Unigene046312 | GABQ01064382 |
| Contig_34290 | Unigene041605 | GABQ01074596 |
| Contig_34333 | Unigene039846 | GABQ01079410 |
| Contig_34344 | Unigene049200 | GABQ01053930 |
| Contig_34363 | Unigene048112 | GABQ01059276 |
| Contig_34376 | Unigene042256 | GABQ01073137 |
| Contig_34377 | Unigene012871 | GABQ01040976 |
| Contig_3443 | Unigene045232 | GABQ01066545 |
| Contig_34471 | Unigene002970 | GABQ01056730 |
| Contig_34490 | Unigene011816 | GABQ01056671 |
| Contig_345 | Unigene005841 | GABQ01014773 |
| Contig_34521 | Unigene043774 | GABQ01069530 |
| Contig_34522 | Unigene041938 | GABQ01073840 |
| Contig_34551 | Unigene045217 | GABQ01066681 |
| Contig_34571 | Unigene004665 | GABQ01071412 |
| Contig_34600 | Unigene024755 | GABQ01035225 |
| Contig_34603 | Unigene026461 | GABQ01025940 |
| Contig_34617 | Unigene018247 | GABQ01018970 |
| Contig_34620 | Unigene005723 | GABQ01068254 |
| Contig_34643 | Unigene031680 | GABQ01031880 |
| Contig_34684 | Unigene039767 | GABQ01079748 |
| Contig_34692 | Unigene000312 | GABQ01079609 |
| Contig_34713 | Unigene045599 | GABQ01065548 |
| Contig_34789 | Unigene005472 | GABQ01070481 |
| Contig_34824 | Unigene031504 |  |
| Contig_34837 | Unigene040698 | GABQ01076773 |
| Contig_34847 | Unigene045050 | GABQ01066985 |
| Contig_34857 | Unigene011158 | GABQ01045071 |
| Contig_34859 | Unigene041649 | GABQ01080975 |
| Contig_34864 | Unigene042630 | GABQ01072162 |
| Contig_34879 | Unigene001128 | GABQ01066374 |
| Contig_3489 | Unigene029301 | GABQ01057950 |
| Contig_34894 | Unigene045821 | GABQ01065367 |
| Contig_34907 | Unigene050355 | GABQ01045792 |
| Contig_34908 | Unigene042658 | GABQ01072099 |
| Contig_34930 | Unigene043697 | GABQ01069720 |
| Contig_34940 | Unigene021850 | GABQ01052792 |
| Contig_34971 | Unigene010682 |  |
| Contig_34989 | Unigene020439 | GABQ01048263 |
| Contig_35001 | Unigene058889 | GABQ01043743 |
| Contig_35002 | Unigene028114 | GABQ01063165 |
| Contig_35006 | Unigene050841 | GABQ01041037 |
| Contig_3507 | Unigene003143 | GABQ01080855 |
| Contig_35086 | Unigene051740 | GABQ01026005 |
| Contig_35102 | Unigene023529 |  |
| Contig_35127 | Unigene028333 | GABQ01036864 |
| Contig_35130 | Unigene003962 | GABQ01076136 |
| Contig_35150 | Unigene050856 | GABQ01041489 |
| Contig_35254 | Unigene024368 | GABQ01045896 |
| Contig_35301 | Unigene004136 | GABQ01075133 |
| Contig_35308 | Unigene003494 | GABQ01078884 |
| Contig_35351 | Unigene013355 | GABQ01035181 |
| Contig_35358 | Unigene001710 | GABQ01078298 |
| Contig_35382 | Unigene001859 | GABQ01077149 |
| Contig_35412 | Unigene040057 | GABQ01078627 |
| Contig_35414 | Unigene012393 | GABQ01016554 |
| Contig_35431 | Unigene040025 | GABQ01078742 |
| Contig_35450 | Unigene026313 | GABQ01055501 |
| Contig_35458 | Unigene018997 | GABQ01025901 |
| Contig_35467 | Unigene049443 | GABQ01053068 |
| Contig_35501 | Unigene037112 | GABQ01069411 |
| Contig_35503 | Unigene050612 | GABQ01044171 |
| Contig_35529 | Unigene033550 | GABQ01056775 |
| Contig_35540 | Unigene020360 | GABQ01040152 |
| Contig_35565 | Unigene016384 | GABQ01068232 |
| Contig_35575 | Unigene004332 | GABQ01079581 |
| Contig_35679 | Unigene048830 | GABQ01056181 |
| Contig_35747 | Unigene027613 | GABQ01035804 |
| Contig_35759 | Unigene106366 | GABQ01019277 |
| Contig_35774 | Unigene043504 | GABQ01070069 |
| Contig_3584 | Unigene038012 | GABQ01076824 |
| Contig_35850 | Unigene028438 | GABQ01047081 |
| Contig_35909 | Unigene000272 | GABQ01079297 |
| Contig_35996 | Unigene051056 | GABQ01037398 |
| Contig_36021 | Unigene010092 | GABQ01010542 |
| Contig_36050 | Unigene051280 | GABQ01036158 |
| Contig_36249 | Unigene014779 | GABQ01062947 |
| Contig_36258 | Unigene004983 | GABQ01068821 |
| Contig_36317 | Unigene012061 | GABQ01053877 |
| Contig_36378 | Unigene003479 | GABQ01078983 |
| Contig_36389 | Unigene020373 | GABQ01075410 |
| Contig_36395 | Unigene052172 | GABQ01015455 |
| Contig_36408 | Unigene040907 | GABQ01076268 |
| Contig_36409 | Unigene012429 | GABQ01018937 |
| Contig_36432 | Unigene008648 | GABQ01026994 |
| Contig_36438 | Unigene039616 | GABQ01080549 |
| Contig_36456 | Unigene002880 | GABQ01063361 |
| Contig_36469 | Unigene041458 | GABQ01074978 |
| Contig_36511 | Unigene051852 | GABQ01023496 |
| Contig_36541 | Unigene002681 | GABQ01067740 |
| Contig_36557 | Unigene004164 | GABQ01074905 |
| Contig_36584 | Unigene111305 | GABQ01038956 |
| Contig_366 | Unigene041270 | GABQ01075314 |
| Contig_36622 | Unigene033085 |  |
| Contig_36645 | Unigene048841 | GABQ01056183 |
| Contig_36709 | Unigene102284 | GABQ01023042 |
| Contig_36759 | Unigene001592 | GABQ01079342 |
| Contig_36780 | Unigene043442 | GABQ01070251 |
| Contig_36802 | Unigene045558 | GABQ01065910 |
| Contig_36877 | Unigene042812 | GABQ01071634 |
| Contig_36914 | Unigene012980 | GABQ01032632 |
| Contig_36930 | Unigene043508 | GABQ01070001 |
| Contig_36961 | Unigene015361 | GABQ01042061 |
| Contig_36985 | Unigene011784 | GABQ01015915 |
| Contig_37066 | Unigene015054 | GABQ01065120 |
| Contig_37070 | Unigene015029 | GABQ01031791 |
| Contig_37173 | Unigene004332 | GABQ01079581 |
| Contig_37176 | Unigene044490 | GABQ01067989 |
| Contig_37177 | Unigene047371 | GABQ01060969 |
| Contig_37203 | Unigene042591 | GABQ01072214 |
| Contig_37235 | Unigene045175 | GABQ01066774 |
| Contig_3730 | Unigene000224 | GABQ01070152 |
| Contig_37300 | Unigene014436 | GABQ01029820 |
| Contig_37310 | Unigene046112 | GABQ01064828 |
| Contig_37320 | Unigene039599 | GABQ01080659 |
| Contig_37327 | Unigene027670 | GABQ01040514 |
| Contig_37370 | Unigene013900 | GABQ01018353 |
| Contig_37400 | Unigene007046 | GABQ01026424 |
| Contig_37412 | Unigene001006 | GABQ01076192 |
| Contig_37432 | Unigene051044 | GABQ01039103 |
| Contig_37437 | Unigene002997 | GABQ01060587 |
| Contig_37480 | Unigene048118 | GABQ01059280 |
| Contig_37498 | Unigene017684 | GABQ01034529 |
| Contig_37529 | Unigene008571 | GABQ01036012 |
| Contig_3754 | Unigene033004 | GABQ01069896 |
| Contig_37562 | Unigene012761 | GABQ01071293 |
| Contig_37635 | Unigene005751 | GABQ01038606 |
| Contig_37651 | Unigene011894 | GABQ01080879 |
| Contig_37677 | Unigene020794 | GABQ01039987 |
| Contig_37699 | Unigene042491 | GABQ01072507 |
| Contig_37725 | Unigene004767 | GABQ01073292 |
| Contig_37791 | Unigene001117 |  |
| Contig_37808 | Unigene051660 | GABQ01027751 |
| Contig_37811 | Unigene049425 | GABQ01053060 |
| Contig_37850 | Unigene009299 | GABQ01058807 |
| Contig_37867 | Unigene003228 | GABQ01080357 |
| Contig_37871 | Unigene022057 | GABQ01009411 |
| Contig_37880 | Unigene037001 | GABQ01078107 |
| Contig_3789 | Unigene005253 | GABQ01065078 |
| Contig_37893 | Unigene002073 | GABQ01077116 |
| Contig_37952 | Unigene001398 |  |
| Contig_37981 | Unigene005085 | GABQ01069142 |
| Contig_37985 | Unigene021508 | GABQ01047212 |
| Contig_38006 | Unigene045060 | GABQ01066991 |
| Contig_38007 | Unigene048202 | GABQ01058997 |
| Contig_38018 | Unigene001398 |  |
| Contig_38020 | Unigene020949 | GABQ01054507 |
| Contig_3803 | Unigene018792 | GABQ01077600 |
| Contig_38055 | Unigene000137 | GABQ01080375 |
| Contig_38059 | Unigene009494 | GABQ01006685 |
| Contig_38079 | Unigene118729 | GABQ01005669 |
| Contig_38159 | Unigene000791 | GABQ01075617 |
| Contig_38192 | Unigene008137 | GABQ01020756 |
| Contig_38194 | Unigene017602 | GABQ01016589 |
| Contig_38200 | Unigene051224 | GABQ01036906 |
| Contig_38270 | Unigene028558 | GABQ01047227 |
| Contig_38295 | Unigene049170 | GABQ01054625 |
| Contig_38296 | Unigene003484 | GABQ01078942 |
| Contig_38311 | Unigene050689 | GABQ01042703 |
| Contig_38320 | Unigene040927 | GABQ01076180 |
| Contig_38350 | Unigene004157 | GABQ01075029 |
| Contig_38402 | Unigene039880 | GABQ01079318 |
| Contig_38430 | Unigene016643 | GABQ01033072 |
| Contig_38440 | Unigene042872 | GABQ01071461 |
| Contig_38499 | Unigene009043 | GABQ01032620 |
| Contig_38515 | Unigene042302 | GABQ01072982 |
| Contig_38518 | Unigene048329 | GABQ01058498 |
| Contig_38574 | Unigene017850 | GABQ01059026 |
| Contig_38637 | Unigene045578 | GABQ01065599 |
| Contig_38643 | Unigene046385 | GABQ01064211 |
| Contig_38662 | Unigene050754 | GABQ01042421 |
| Contig_38686 | Unigene048637 | GABQ01057291 |
| Contig_38713 | Unigene030346 | GABQ01062477 |
| Contig_38733 | Unigene039116 | GABQ01065656 |
| Contig_38806 | Unigene035420 | GABQ01024114 |
| Contig_38816 | Unigene040417 | GABQ01077497 |
| Contig_38840 | Unigene041973 | GABQ01073821 |
| Contig_38866 | Unigene025404 | GABQ01051378 |
| Contig_38904 | Unigene043292 | GABQ01070516 |
| Contig_39004 | Unigene004666 | GABQ01071413 |
| Contig_39016 | Unigene043177 | GABQ01070800 |
| Contig_39068 | Unigene020877 | GABQ01039988 |
| Contig_39075 | Unigene010953 | GABQ01051108 |
| Contig_39081 | Unigene043560 | GABQ01069826 |
| Contig_39083 | Unigene065327 | GABQ01016758 |
| Contig_39333 | Unigene007111 | GABQ01018900 |
| Contig_39357 | Unigene003752 | GABQ01077447 |
| Contig_39375 | Unigene052100 | GABQ01016692 |
| Contig_39396 | Unigene037658 | GABQ01072799 |
| Contig_39426 | Unigene051376 | GABQ01033950 |
| Contig_39437 | Unigene050181 | GABQ01047114 |
| Contig_39443 | Unigene019899 | GABQ01074786 |
| Contig_39463 | Unigene007714 | GABQ01064176 |
| Contig_39464 | Unigene036780 | GABQ01080799 |
| Contig_39483 | Unigene025661 | GABQ01027686 |
| Contig_39499 | Unigene021856 | GABQ01024037 |
| Contig_39524 | Unigene040372 | GABQ01077638 |
| Contig_39567 | Unigene041892 | GABQ01069071 |
| Contig_39589 | Unigene034159 | GABQ01039086 |
| Contig_39596 | Unigene005163 | GABQ01066813 |
| Contig_3962 | Unigene043282 | GABQ01070470 |
| Contig_39675 | Unigene016391 | GABQ01045480 |
| Contig_39691 | Unigene003126 |  |
| Contig_39717 | Unigene012438 | GABQ01020790 |
| Contig_39724 | Unigene045927 | GABQ01064876 |
| Contig_39751 | Unigene008056 | GABQ01059640 |
| Contig_39763 | Unigene000361 | GABQ01079618 |
| Contig_39767 | Unigene113486 | GABQ01008176 |
| Contig_39770 | Unigene009351 | GABQ01021388 |
| Contig_39792 | Unigene002067 | GABQ01078557 |
| Contig_39794 | Unigene043102 | GABQ01070968 |
| Contig_39796 | Unigene000402 | GABQ01080378 |
| Contig_39837 | Unigene099518 | GABQ01023025 |
| Contig_39879 | Unigene010788 | GABQ01069742 |
| Contig_39882 | Unigene038681 | GABQ01070514 |
| Contig_39909 | Unigene028547 | GABQ01044973 |
| Contig_39927 | Unigene044962 | GABQ01067166 |
| Contig_39989 | Unigene040073 | GABQ01078586 |
| Contig_40054 | Unigene004942 | GABQ01069007 |
| Contig_40059 | Unigene021486 | GABQ01038841 |
| Contig_40060 | Unigene005142 | GABQ01067023 |
| Contig_40074 | Unigene003244 | GABQ01080244 |
| Contig_40090 | Unigene004625 | GABQ01071728 |
| Contig_40102 | Unigene036033 | GABQ01052484 |
| Contig_4012 | Unigene001072 | GABQ01069558 |
| Contig_40209 | Unigene023875 | GABQ01047475 |
| Contig_40291 | Unigene046354 | GABQ01064282 |
| Contig_40325 | Unigene034930 | GABQ01041797 |
| Contig_40339 | Unigene041199 | GABQ01075477 |
| Contig_40359 | Unigene000921 | GABQ01079371 |
| Contig_40434 | Unigene148845 |  |
| Contig_40448 | Unigene050002 | GABQ01048840 |
| Contig_40450 | Unigene042293 | GABQ01073030 |
| Contig_4047 | Unigene002411 | GABQ01072880 |
| Contig_4048 | Unigene041298 | GABQ01075344 |
| Contig_40482 | Unigene033656 | GABQ01059196 |
| Contig_40505 | Unigene015826 | GABQ01022114 |
| Contig_40553 | Unigene000200 | GABQ01078249 |
| Contig_40559 | Unigene040149 | GABQ01078311 |
| Contig_40605 | Unigene023944 | GABQ01068934 |
| Contig_40643 | Unigene001399 | GABQ01080914 |
| Contig_40683 | Unigene046644 | GABQ01063646 |
| Contig_40690 | Unigene042619 | GABQ01072158 |
| Contig_40704 | Unigene039880 | GABQ01079318 |
| Contig_40721 | Unigene049874 | GABQ01050017 |
| Contig_4077 | Unigene064314 | GABQ01019745 |
| Contig_40789 | Unigene049891 | GABQ01049883 |
| Contig_40859 | Unigene002137 | GABQ01077438 |
| Contig_40883 | Unigene047710 | GABQ01060637 |
| Contig_40965 | Unigene048674 | GABQ01056978 |
| Contig_40968 | Unigene046131 | GABQ01064656 |
| Contig_40999 | Unigene062989 |  |
| Contig_41041 | Unigene005540 | GABQ01057996 |
| Contig_41052 | Unigene015259 | GABQ01028183 |
| Contig_41058 | Unigene002364 | GABQ01071284 |
| Contig_41112 | Unigene001398 |  |
| Contig_41149 | Unigene017337 |  |
| Contig_41159 | Unigene041897 | GABQ01073929 |
| Contig_41220 | Unigene048973 | GABQ01055830 |
| Contig_41233 | Unigene047245 | GABQ01062064 |
| Contig_41252 | Unigene002698 | GABQ01070306 |
| Contig_41266 | Unigene035762 | GABQ01057960 |
| Contig_41331 | Unigene047293 | GABQ01061786 |
| Contig_41401 | Unigene047994 | GABQ01059685 |
| Contig_41414 | Unigene034411 | GABQ01068150 |
| Contig_41486 | Unigene001793 | GABQ01077573 |
| Contig_41501 | Unigene039597 | GABQ01080670 |
| Contig_41552 | Unigene003199 | GABQ01080569 |
| Contig_41582 | Unigene031510 | GABQ01058609 |
| Contig_41584 | Unigene047405 | GABQ01061514 |
| Contig_41602 | Unigene147010 | GABQ01007242 |
| Contig_41650 | Unigene110633 | GABQ01071065 |
| Contig_41654 | Unigene028976 | GABQ01044565 |
| Contig_41715 | Unigene028841 | GABQ01024076 |
| Contig_41717 | Unigene045235 | GABQ01066652 |
| Contig_41730 | Unigene039942 | GABQ01079074 |
| Contig_41801 | Unigene047941 | GABQ01059886 |
| Contig_41832 | Unigene017689 | GABQ01068256 |
| Contig_41931 | Unigene046771 | GABQ01063179 |
| Contig_41944 | Unigene023764 | GABQ01034886 |
| Contig_42072 | Unigene015794 | GABQ01060702 |
| Contig_42087 | Unigene000978 | GABQ01078622 |
| Contig_42097 | Unigene041664 | GABQ01074466 |
| Contig_42114 | Unigene048671 | GABQ01057049 |
| Contig_42115 | Unigene001305 | GABQ01074218 |
| Contig_4217 | Unigene005134 | GABQ01067175 |
| Contig_422 | Unigene047179 | GABQ01062205 |
| Contig_42200 | Unigene015511 | GABQ01016576 |
| Contig_42217 | Unigene024152 | GABQ01044823 |
| Contig_42256 | Unigene052286 | GABQ01012407 |
| Contig_42312 | Unigene033950 | GABQ01048699 |
| Contig_42315 | Unigene002187 | GABQ01074087 |
| Contig_42342 | Unigene029672 | GABQ01034908 |
| Contig_42346 | Unigene047015 | GABQ01062664 |
| Contig_42364 | Unigene001245 | GABQ01078032 |
| Contig_42386 | Unigene027600 | GABQ01072462 |
| Contig_42435 | Unigene044043 | GABQ01068979 |
| Contig_42436 | Unigene026694 | GABQ01060770 |
| Contig_42460 | Unigene014030 | GABQ01055679 |
| Contig_42482 | Unigene003327 | GABQ01079808 |
| Contig_42504 | Unigene016908 | GABQ01014299 |
| Contig_42515 | Unigene014046 |  |
| Contig_42524 | Unigene025417 | GABQ01060606 |
| Contig_42588 | Unigene003145 | GABQ01080851 |
| Contig_42604 | Unigene040219 | GABQ01078109 |
| Contig_42748 | Unigene015318 | GABQ01023993 |
| Contig_42819 | Unigene046967 | GABQ01062790 |
| Contig_42844 | Unigene001822 | GABQ01077324 |
| Contig_42866 | Unigene032726 | GABQ01033127 |
| Contig_42904 | Unigene046343 | GABQ01064244 |
| Contig_42911 | Unigene037024 | GABQ01080926 |
| Contig_42963 | Unigene041022 | GABQ01075972 |
| Contig_43012 | Unigene018025 | GABQ01069929 |
| Contig_43041 | Unigene020410 | GABQ01074718 |
| Contig_43064 | Unigene030603 | GABQ01036110 |
| Contig_43073 | Unigene038544 | GABQ01071242 |
| Contig_43096 | Unigene103910 | GABQ01001412 |
| Contig_43169 | Unigene051304 | GABQ01035562 |
| Contig_4321 | Unigene001865 | GABQ01078046 |
| Contig_43214 | Unigene080282 | GABQ01018584 |
| Contig_43256 | Unigene124576 | GABQ01010035 |
| Contig_43262 | Unigene091240 | GABQ01027892 |
| Contig_43275 | Unigene048438 | GABQ01058049 |
| Contig_43286 | Unigene035826 | GABQ01052823 |
| Contig_43304 | Unigene092439 |  |
| Contig_4332 | Unigene045699 | GABQ01065670 |
| Contig_43322 | Unigene039478 | GABQ01030949 |
| Contig_43341 | Unigene043034 | GABQ01071073 |
| Contig_4337 | Unigene003675 | GABQ01077862 |
| Contig_43375 | Unigene040442 | GABQ01077477 |
| Contig_43395 | Unigene040555 | GABQ01077166 |
| Contig_43401 | Unigene103935 | GABQ01070715 |
| Contig_43431 | Unigene004367 | GABQ01073664 |
| Contig_43453 | Unigene015301 | GABQ01026468 |
| Contig_43490 | Unigene042617 | GABQ01072042 |
| Contig_43506 | Unigene000681 | GABQ01079071 |
| Contig_43552 | Unigene008023 | GABQ01062368 |
| Contig_436 | Unigene004085 | GABQ01075385 |
| Contig_43624 | Unigene017248 | GABQ01013786 |
| Contig_43642 | Unigene049795 | GABQ01050146 |
| Contig_43662 | Unigene000744 | GABQ01078054 |
| Contig_43745 | Unigene030572 | GABQ01057494 |
| Contig_43810 | Unigene002743 | GABQ01068028 |
| Contig_43815 | Unigene001398 |  |
| Contig_43857 | Unigene025445 | GABQ01017242 |
| Contig_43859 | Unigene020366 | GABQ01000805 |
| Contig_43865 | Unigene000895 | GABQ01080922 |
| Contig_43899 | Unigene042757 | GABQ01071811 |
| Contig_43920 | Unigene016490 | GABQ01026477 |
| Contig_43974 | Unigene042608 | GABQ01072003 |
| Contig_43991 | Unigene042702 | GABQ01071970 |
| Contig_44009 | Unigene003356 | GABQ01079690 |
| Contig_44014 | Unigene041473 | GABQ01074937 |
| Contig_44024 | Unigene049610 | GABQ01051926 |
| Contig_44027 | Unigene048813 | GABQ01056363 |
| Contig_44048 | Unigene052269 | GABQ01012837 |
| Contig_4409 | Unigene047212 | GABQ01062160 |
| Contig_4411 | Unigene002123 | GABQ01074634 |
| Contig_44115 | Unigene045101 | GABQ01066888 |
| Contig_44162 | Unigene049508 | GABQ01052383 |
| Contig_44168 | Unigene042267 | GABQ01073065 |
| Contig_44169 | Unigene110071 | GABQ01016306 |
| Contig_44219 | Unigene009634 | GABQ01050745 |
| Contig_4424 | Unigene010907 | GABQ01061320 |
| Contig_44345 | Unigene019359 | GABQ01018975 |
| Contig_4438 | Unigene004834 | GABQ01070018 |
| Contig_44411 | Unigene002082 | GABQ01078065 |
| Contig_44440 | Unigene042157 | GABQ01073344 |
| Contig_44491 | Unigene001621 | GABQ01078671 |
| Contig_44557 | Unigene017460 | GABQ01056147 |
| Contig_44564 | Unigene029993 | GABQ01029362 |
| Contig_44648 | Unigene123256 |  |
| Contig_44682 | Unigene043999 | GABQ01069065 |
| Contig_44691 | Unigene011363 | GABQ01060751 |
| Contig_44707 | Unigene003291 | GABQ01079989 |
| Contig_44708 | Unigene050474 | GABQ01045008 |
| Contig_44792 | Unigene013469 | GABQ01039209 |
| Contig_44822 | Unigene028182 | GABQ01037596 |
| Contig_44851 | Unigene044731 | GABQ01067502 |
| Contig_44857 | Unigene049762 | GABQ01050683 |
| Contig_44885 | Unigene033723 | GABQ01074414 |
| Contig_44917 | Unigene003745 | GABQ01077471 |
| Contig_44965 | Unigene002644 | GABQ01074114 |
| Contig_45082 | Unigene093039 | GABQ01012519 |
| Contig_45107 | Unigene045196 | GABQ01066710 |
| Contig_45134 | Unigene009091 | GABQ01007630 |
| Contig_45176 | Unigene004698 | GABQ01071199 |
| Contig_45187 | Unigene048793 | GABQ01056642 |
| Contig_45222 | Unigene044376 | GABQ01068279 |
| Contig_45304 | Unigene052598 |  |
| Contig_45305 | Unigene013354 | GABQ01025237 |
| Contig_45308 | Unigene022275 | GABQ01020221 |
| Contig_45372 | Unigene020622 | GABQ01017222 |
| Contig_45384 | Unigene007869 | GABQ01020123 |
| Contig_45385 | Unigene034545 | GABQ01021534 |
| Contig_45394 | Unigene045687 | GABQ01065665 |
| Contig_45403 | Unigene150372 |  |
| Contig_45415 | Unigene027162 | GABQ01003954 |
| Contig_45418 | Unigene040118 | GABQ01078432 |
| Contig_45454 | Unigene005433 | GABQ01062127 |
| Contig_45515 | Unigene051204 | GABQ01037157 |
| Contig_45544 | Unigene004418 | GABQ01073161 |
| Contig_45546 | Unigene007991 | GABQ01057999 |
| Contig_45577 | Unigene005830 | GABQ01064566 |
| Contig_45581 | Unigene004857 | GABQ01069861 |
| Contig_45582 | Unigene048900 | GABQ01056287 |
| Contig_45598 | Unigene028716 | GABQ01017834 |
| Contig_45664 | Unigene015055 | GABQ01024597 |
| Contig_45701 | Unigene051395 | GABQ01033963 |
| Contig_45710 | Unigene030953 | GABQ01056954 |
| Contig_45791 | Unigene009027 | GABQ01029236 |
| Contig_45832 | Unigene020418 | GABQ01053694 |
| Contig_45840 | Unigene026231 | GABQ01060300 |
| Contig_45843 | Unigene040219 | GABQ01078109 |
| Contig_45846 | Unigene001565 | GABQ01079383 |
| Contig_45858 | Unigene004505 | GABQ01072565 |
| Contig_45870 | Unigene005720 | GABQ01068581 |
| Contig_45890 | Unigene033131 | GABQ01047503 |
| Contig_45904 | Unigene042155 | GABQ01073297 |
| Contig_45912 | Unigene021743 | GABQ01035216 |
| Contig_45922 | Unigene040287 | GABQ01077894 |
| Contig_45923 | Unigene022481 | GABQ01033476 |
| Contig_45928 | Unigene049197 | GABQ01054545 |
| Contig_45930 | Unigene010125 | GABQ01020774 |
| Contig_45942 | Unigene044708 | GABQ01067615 |
| Contig_45971 | Unigene012588 | GABQ01043936 |
| Contig_45988 | Unigene051518 | GABQ01030963 |
| Contig_45998 | Unigene051154 | GABQ01037635 |
| Contig_460 | Unigene005354 | GABQ01063846 |
| Contig_46056 | Unigene006988 | GABQ01022046 |
| Contig_46091 | Unigene038012 | GABQ01076824 |
| Contig_46109 | Unigene000354 | GABQ01080552 |
| Contig_46133 | Unigene033754 | GABQ01053922 |
| Contig_46136 | Unigene046394 | GABQ01064119 |
| Contig_46141 | Unigene008553 | GABQ01057857 |
| Contig_46202 | Unigene045981 | GABQ01065034 |
| Contig_46208 | Unigene002714 | GABQ01066551 |
| Contig_46237 | Unigene000620 | GABQ01080937 |
| Contig_46296 | Unigene004303 | GABQ01074093 |
| Contig_46305 | Unigene045596 | GABQ01064752 |
| Contig_46306 | Unigene049378 | GABQ01053412 |
| Contig_46323 | Unigene002764 | GABQ01065149 |
| Contig_46329 | Unigene013218 | GABQ01028173 |
| Contig_46338 | Unigene017618 | GABQ01076785 |
| Contig_46342 | Unigene001177 | GABQ01080769 |
| Contig_46361 | Unigene052464 | GABQ01004919 |
| Contig_46433 | Unigene040651 | GABQ01076871 |
| Contig_46441 | Unigene044043 | GABQ01068979 |
| Contig_46444 | Unigene050693 | GABQ01042998 |
| Contig_46498 | Unigene045984 | GABQ01064970 |
| Contig_46511 | Unigene039659 | GABQ01080255 |
| Contig_46520 | Unigene013143 | GABQ01054227 |
| Contig_46536 | Unigene005927 | GABQ01044925 |
| Contig_46600 | Unigene000374 | GABQ01078712 |
| Contig_46608 | Unigene019548 | GABQ01066696 |
| Contig_46628 | Unigene008533 | GABQ01028677 |
| Contig_46720 | Unigene051232 | GABQ01036669 |
| Contig_46742 | Unigene085040 |  |
| Contig_46828 | Unigene029697 | GABQ01051501 |
| Contig_46848 | Unigene046643 | GABQ01063599 |
| Contig_46875 | Unigene044791 | GABQ01067432 |
| Contig_46917 | Unigene036802 | GABQ01077395 |
| Contig_46919 | Unigene006997 | GABQ01031744 |
| Contig_46922 | Unigene000662 | GABQ01076645 |
| Contig_46949 | Unigene027630 |  |
| Contig_47009 | Unigene020881 | GABQ01051009 |
| Contig_47022 | Unigene000540 | GABQ01072496 |
| Contig_47087 | Unigene001633 | GABQ01078844 |
| Contig_47105 | Unigene004937 | GABQ01069161 |
| Contig_47111 | Unigene001776 | GABQ01077603 |
| Contig_47165 | Unigene041006 | GABQ01076006 |
| Contig_47310 | Unigene028979 | GABQ01013309 |
| Contig_47347 | Unigene043612 | GABQ01069803 |
| Contig_47452 | Unigene013749 |  |
| Contig_47478 | Unigene010112 |  |
| Contig_47505 | Unigene013387 | GABQ01018349 |
| Contig_47659 | Unigene021270 | GABQ01060547 |
| Contig_47676 | Unigene014749 | GABQ01022716 |
| Contig_47761 | Unigene010322 | GABQ01060422 |
| Contig_478 | Unigene008861 | GABQ01075074 |
| Contig_47833 | Unigene005823 | GABQ01022035 |
| Contig_47890 | Unigene148763 | GABQ01026918 |
| Contig_48101 | Unigene036746 | GABQ01078087 |
| Contig_48117 | Unigene010319 | GABQ01010544 |
| Contig_48154 | Unigene049044 | GABQ01055434 |
| Contig_48185 | Unigene042752 | GABQ01071833 |
| Contig_48218 | Unigene039952 | GABQ01079034 |
| Contig_48223 | Unigene005259 | GABQ01065436 |
| Contig_48257 | Unigene000544 | GABQ01070115 |
| Contig_48267 | Unigene007380 | GABQ01042494 |
| Contig_4827 | Unigene013226 | GABQ01022097 |
| Contig_48270 | Unigene021793 | GABQ01022154 |
| Contig_48320 | Unigene002387 | GABQ01075767 |
| Contig_48340 | Unigene042560 | GABQ01072190 |
| Contig_48347 | Unigene044134 | GABQ01068495 |
| Contig_48373 | Unigene051454 | GABQ01032332 |
| Contig_48387 | Unigene013594 | GABQ01013230 |
| Contig_48396 | Unigene036773 | GABQ01066700 |
| Contig_48506 | Unigene068489 | GABQ01021004 |
| Contig_48538 | Unigene016349 | GABQ01015949 |
| Contig_48566 | Unigene014048 | GABQ01063424 |
| Contig_48573 | Unigene022161 | GABQ01021466 |
| Contig_48598 | Unigene048399 | GABQ01058276 |
| Contig_48642 | Unigene015153 | GABQ01018358 |
| Contig_48672 | Unigene001758 | GABQ01077705 |
| Contig_48680 | Unigene000895 | GABQ01080922 |
| Contig_48707 | Unigene037629 | GABQ01065687 |
| Contig_48717 | Unigene044374 | GABQ01068327 |
| Contig_4873 | Unigene043706 | GABQ01069483 |
| Contig_48750 | Unigene044182 | GABQ01068702 |
| Contig_48762 | Unigene052034 | GABQ01019074 |
| Contig_48775 | Unigene043364 | GABQ01070354 |
| Contig_48801 | Unigene155543 | GABQ01025765 |
| Contig_48825 | Unigene021737 | GABQ01052928 |
| Contig_48844 | Unigene041771 | GABQ01074265 |
| Contig_48865 | Unigene044150 | GABQ01068719 |
| Contig_4887 | Unigene038518 | GABQ01072001 |
| Contig_48871 | Unigene019384 |  |
| Contig_48890 | Unigene108057 | GABQ01010392 |
| Contig_48892 | Unigene008628 | GABQ01048893 |
| Contig_48929 | Unigene038091 | GABQ01077755 |
| Contig_48959 | Unigene001223 | GABQ01079085 |
| Contig_49279 | Unigene017976 | GABQ01041906 |
| Contig_49286 | Unigene006783 | GABQ01060025 |
| Contig_49318 | Unigene035678 | GABQ01044993 |
| Contig_4939 | Unigene003748 | GABQ01077480 |
| Contig_49410 | Unigene035474 | GABQ01059198 |
| Contig_49412 | Unigene039590 | GABQ01080724 |
| Contig_49477 | Unigene038008 | GABQ01077832 |
| Contig_49491 | Unigene049017 | GABQ01055537 |
| Contig_49495 | Unigene048501 | GABQ01057366 |
| Contig_49496 | Unigene023054 | GABQ01071584 |
| Contig_49579 | Unigene000370 | GABQ01079958 |
| Contig_4958 | Unigene014277 | GABQ01068539 |
| Contig_49593 | Unigene002560 | GABQ01071704 |
| Contig_4967 | Unigene017638 | GABQ01066622 |
| Contig_49696 | Unigene003230 | GABQ01080334 |
| Contig_49705 | Unigene031984 | GABQ01043991 |
| Contig_49730 | Unigene003126 |  |
| Contig_49760 | Unigene029696 | GABQ01036636 |
| Contig_49769 |  |  |
| Contig_4978 | Unigene040767 | GABQ01076628 |
| Contig_49793 | Unigene137114 | GABQ01044488 |
| Contig_49806 | Unigene037834 | GABQ01079320 |
| Contig_49862 | Unigene049956 | GABQ01049340 |
| Contig_49917 | Unigene109176 | GABQ01004108 |
| Contig_49928 | Unigene012299 | GABQ01029809 |
| Contig_4993 | Unigene007777 | GABQ01053239 |
| Contig_49969 | Unigene015290 | GABQ01008345 |
| Contig_5009 | Unigene034777 | GABQ01057819 |
| Contig_50154 | Unigene043158 | GABQ01070869 |
| Contig_50158 | Unigene025617 | GABQ01061329 |
| Contig_50287 | Unigene049341 | GABQ01053404 |
| Contig_50297 | Unigene034037 | GABQ01064808 |
| Contig_5031 | Unigene002452 | GABQ01070501 |
| Contig_50319 | Unigene007934 | GABQ01044232 |
| Contig_50320 | Unigene035902 | GABQ01022838 |
| Contig_50340 | Unigene045947 | GABQ01065145 |
| Contig_50369 | Unigene046083 | GABQ01064850 |
| Contig_50371 | Unigene012337 | GABQ01010162 |
| Contig_50374 | Unigene048166 | GABQ01059071 |
| Contig_50380 | Unigene016335 | GABQ01042927 |
| Contig_50402 | Unigene124075 | GABQ01015170 |
| Contig_50447 | Unigene037738 | GABQ01075520 |
| Contig_50461 | Unigene042098 | GABQ01073341 |
| Contig_50489 | Unigene024331 | GABQ01050106 |
| Contig_50498 | Unigene045742 | GABQ01065446 |
| Contig_50589 | Unigene044184 | GABQ01068544 |
| Contig_50712 | Unigene049580 | GABQ01051285 |
| Contig_50739 | Unigene003047 | GABQ01048349 |
| Contig_50749 | Unigene036012 | GABQ01059804 |
| Contig_50770 | Unigene049443 | GABQ01053068 |
| Contig_50839 | Unigene000618 | GABQ01068250 |
| Contig_50871 | Unigene002332 | GABQ01071887 |
| Contig_50872 | Unigene133025 | GABQ01013082 |
| Contig_5088 | Unigene043247 | GABQ01070594 |
| Contig_50889 | Unigene110545 | GABQ01003810 |
| Contig_50938 | Unigene012679 | GABQ01055676 |
| Contig_5094 | Unigene043562 | GABQ01069900 |
| Contig_50959 | Unigene011019 | GABQ01033422 |
| Contig_50984 | Unigene031289 | GABQ01066103 |
| Contig_51059 | Unigene001861 |  |
| Contig_5106 | Unigene010490 | GABQ01066010 |
| Contig_51101 | Unigene018891 | GABQ01031817 |
| Contig_51115 | Unigene016187 | GABQ01042515 |
| Contig_51118 | Unigene017307 | GABQ01011387 |
| Contig_51144 | Unigene001596 | GABQ01079202 |
| Contig_51247 | Unigene049305 | GABQ01054078 |
| Contig_51276 | Unigene029993 | GABQ01029362 |
| Contig_5128 | Unigene002374 | GABQ01074431 |
| Contig_51294 | Unigene032944 | GABQ01048287 |
| Contig_51297 | Unigene001220 | GABQ01079286 |
| Contig_51298 | Unigene047064 | GABQ01062401 |
| Contig_51336 | Unigene006547 | GABQ01011340 |
| Contig_51380 | Unigene106827 | GABQ01069702 |
| Contig_51391 | Unigene143104 | GABQ01046263 |
| Contig_5142 | Unigene028945 | GABQ01011008 |
| Contig_51480 | Unigene051618 | GABQ01030452 |
| Contig_51494 | Unigene041238 | GABQ01075412 |
| Contig_5151 | Unigene002089 | GABQ01080942 |
| Contig_51510 | Unigene041808 | GABQ01074187 |
| Contig_51561 | Unigene149244 |  |
| Contig_51611 | Unigene033337 | GABQ01023452 |
| Contig_51622 | Unigene042566 | GABQ01072284 |
| Contig_51635 | Unigene001882 | GABQ01078823 |
| Contig_51637 | Unigene002444 | GABQ01073723 |
| Contig_51660 | Unigene027783 |  |
| Contig_51698 | Unigene100369 | GABQ01023029 |
| Contig_51705 | Unigene004092 | GABQ01075375 |
| Contig_51739 | Unigene105779 |  |
| Contig_51758 | Unigene028413 | GABQ01003689 |
| Contig_5180 | Unigene001801 | GABQ01077402 |
| Contig_51826 | Unigene041412 | GABQ01075041 |
| Contig_51833 | Unigene025038 | GABQ01033104 |
| Contig_51837 | Unigene078823 | GABQ01018578 |
| Contig_51892 | Unigene052448 | GABQ01005531 |
| Contig_51915 | Unigene052021 | GABQ01019690 |
| Contig_51958 | Unigene043041 | GABQ01071075 |
| Contig_52050 | Unigene010610 | GABQ01045868 |
| Contig_52051 | Unigene047423 | GABQ01061518 |
| Contig_52071 | Unigene076491 | GABQ01009923 |
| Contig_52082 | Unigene120227 | GABQ01021196 |
| Contig_52105 | Unigene004712 | GABQ01071177 |
| Contig_52130 | Unigene010161 | GABQ01017724 |
| Contig_52159 | Unigene006868 | GABQ01045330 |
| Contig_52193 | Unigene035107 | GABQ01071565 |
| Contig_52204 | Unigene052254 | GABQ01009071 |
| Contig_52225 | Unigene050416 | GABQ01045400 |
| Contig_52237 | Unigene008206 | GABQ01076140 |
| Contig_52239 | Unigene043631 | GABQ01069612 |
| Contig_52242 | Unigene019837 | GABQ01071334 |
| Contig_52258 | Unigene004209 | GABQ01074658 |
| Contig_52361 | Unigene040055 | GABQ01078633 |
| Contig_52375 | Unigene161402 | GABQ01022646 |
| Contig_52379 | Unigene008924 | GABQ01057230 |
| Contig_52381 | Unigene044823 | GABQ01067386 |
| Contig_52399 | Unigene011481 | GABQ01051979 |
| Contig_52435 | Unigene000553 | GABQ01080889 |
| Contig_52538 | Unigene014129 | GABQ01030812 |
| Contig_52542 | Unigene021234 |  |
| Contig_52576 | Unigene009555 | GABQ01012283 |
| Contig_52604 | Unigene018435 |  |
| Contig_52611 | Unigene047088 | GABQ01062194 |
| Contig_52619 | Unigene036492 | GABQ01015447 |
| Contig_52629 | Unigene006999 | GABQ01022047 |
| Contig_52631 | Unigene005313 | GABQ01064733 |
| Contig_52676 | Unigene029580 | GABQ01031862 |
| Contig_52677 | Unigene052271 | GABQ01012839 |
| Contig_52684 | Unigene015690 | GABQ01025876 |
| Contig_5269 | Unigene000044 | GABQ01076881 |
| Contig_52740 | Unigene139328 |  |
| Contig_52766 | Unigene046975 | GABQ01062241 |
| Contig_52776 | Unigene026844 | GABQ01052006 |
| Contig_52778 | Unigene015053 | GABQ01075278 |
| Contig_52838 | Unigene040731 | GABQ01076627 |
| Contig_52844 | Unigene030556 | GABQ01042819 |
| Contig_52846 | Unigene015165 | GABQ01006698 |
| Contig_52865 | Unigene041196 | GABQ01075546 |
| Contig_52890 | Unigene119542 | GABQ01067683 |
| Contig_52908 | Unigene029584 | GABQ01063433 |
| Contig_52994 | Unigene077784 |  |
| Contig_53000 | Unigene041439 | GABQ01075008 |
| Contig_53020 | Unigene039189 | GABQ01068825 |
| Contig_53118 | Unigene046011 | GABQ01065009 |
| Contig_53119 | Unigene022619 | GABQ01022157 |
| Contig_53147 | Unigene001118 | GABQ01080353 |
| Contig_53154 | Unigene052596 |  |
| Contig_53266 | Unigene067630 |  |
| Contig_53324 | Unigene005216 | GABQ01066069 |
| Contig_53378 | Unigene109206 | GABQ01007828 |
| Contig_53414 | Unigene048865 | GABQ01056099 |
| Contig_53531 | Unigene043161 | GABQ01070870 |
| Contig_53566 | Unigene007683 |  |
| Contig_53625 | Unigene039373 | GABQ01040194 |
| Contig_53634 | Unigene029993 | GABQ01029362 |
| Contig_53654 | Unigene012214 | GABQ01016552 |
| Contig_53691 | Unigene131980 | GABQ01055262 |
| Contig_5372 | Unigene041516 | GABQ01074842 |
| Contig_53755 | Unigene024190 | GABQ01049556 |
| Contig_53762 | Unigene046207 | GABQ01064560 |
| Contig_53822 | Unigene047058 | GABQ01062533 |
| Contig_53828 | Unigene052059 | GABQ01017876 |
| Contig_53844 | Unigene044542 | GABQ01067836 |
| Contig_53863 | Unigene127666 |  |
| Contig_53864 | Unigene004684 | GABQ01071309 |
| Contig_53899 | Unigene048105 | GABQ01059345 |
| Contig_53925 | Unigene153922 |  |
| Contig_53931 | Unigene042141 | GABQ01073420 |
| Contig_53935 | Unigene004355 | GABQ01073978 |
| Contig_53942 | Unigene003956 | GABQ01076196 |
| Contig_53950 | Unigene002125 | GABQ01074856 |
| Contig_53963 | Unigene092535 | GABQ01007788 |
| Contig_53997 | Unigene041203 | GABQ01075556 |
| Contig_54082 | Unigene041518 | GABQ01074777 |
| Contig_54100 | Unigene048925 | GABQ01056014 |
| Contig_54114 | Unigene020925 | GABQ01032678 |
| Contig_54163 | Unigene039663 | GABQ01080210 |
| Contig_54196 | Unigene044024 | GABQ01069001 |
| Contig_54293 | Unigene052410 | GABQ01007710 |
| Contig_54329 | Unigene094279 |  |
| Contig_54332 | Unigene070807 | GABQ01010691 |
| Contig_54426 | Unigene134790 | GABQ01011704 |
| Contig_54750 | Unigene137108 | GABQ01026324 |
| Contig_54751 | Unigene029993 | GABQ01029362 |
| Contig_54762 | Unigene049773 | GABQ01050557 |
| Contig_54840 | Unigene046626 | GABQ01063174 |
| Contig_54919 | Unigene050442 | GABQ01045285 |
| Contig_54970 | Unigene041334 | GABQ01075263 |
| Contig_55031 | Unigene020736 | GABQ01031365 |
| Contig_55064 | Unigene028001 | GABQ01042264 |
| Contig_55065 | Unigene004255 | GABQ01074388 |
| Contig_55089 | Unigene029314 |  |
| Contig_55108 | Unigene029582 | GABQ01041167 |
| Contig_55129 | Unigene020184 | GABQ01018980 |
| Contig_55139 | Unigene036613 |  |
| Contig_55180 | Unigene049247 | GABQ01054170 |
| Contig_55371 | Unigene037034 | GABQ01079586 |
| Contig_55414 | Unigene012566 | GABQ01036815 |
| Contig_55420 | Unigene019643 | GABQ01059647 |
| Contig_55510 | Unigene020601 | GABQ01019559 |
| Contig_55520 | Unigene068212 | GABQ01049766 |
| Contig_55732 | Unigene138363 |  |
| Contig_55778 | Unigene141941 |  |
| Contig_5592 | Unigene046014 | GABQ01065011 |
| Contig_56 | Unigene001771 | GABQ01078134 |
| Contig_5606 | Unigene004035 | GABQ01075692 |
| Contig_56171 | Unigene041486 | GABQ01074915 |
| Contig_56198 | Unigene042188 | GABQ01073325 |
| Contig_56231 | Unigene038678 | GABQ01075448 |
| Contig_56258 | Unigene060213 | GABQ01013375 |
| Contig_56377 | Unigene025388 |  |
| Contig_56405 | Unigene050053 | GABQ01048176 |
| Contig_56410 | Unigene013175 | GABQ01032636 |
| Contig_56413 | Unigene040843 | GABQ01076445 |
| Contig_56439 | Unigene008040 | GABQ01078631 |
| Contig_56455 | Unigene054783 | GABQ01014409 |
| Contig_56462 | Unigene036430 | GABQ01068651 |
| Contig_56469 | Unigene052678 |  |
| Contig_56580 | Unigene126217 | GABQ01013588 |
| Contig_56591 | Unigene060367 | GABQ01070788 |
| Contig_56604 | Unigene005700 | GABQ01044650 |
| Contig_56607 | Unigene000441 | GABQ01077435 |
| Contig_56611 | Unigene000896 | GABQ01080911 |
| Contig_56635 | Unigene037281 | GABQ01078893 |
| Contig_56658 | Unigene035609 |  |
| Contig_56664 | Unigene149244 |  |
| Contig_56676 | Unigene159971 | GABQ01027557 |
| Contig_56708 | Unigene138737 | GABQ01017602 |
| Contig_56710 | Unigene052523 | GABQ01001313 |
| Contig_56723 | Unigene113775 |  |
| Contig_56810 | Unigene024832 |  |
| Contig_56848 | Unigene028315 | GABQ01064325 |
| Contig_56875 | Unigene147126 | GABQ01018834 |
| Contig_56894 | Unigene013518 | GABQ01007648 |
| Contig_56916 | Unigene064305 |  |
| Contig_56918 | Unigene104322 | GABQ01020539 |
| Contig_56963 | Unigene033327 | GABQ01034287 |
| Contig_56980 | Unigene045540 | GABQ01065940 |
| Contig_56992 | Unigene035081 | GABQ01028825 |
| Contig_57038 | Unigene019196 | GABQ01063034 |
| Contig_57107 | Unigene010258 | GABQ01060882 |
| Contig_57111 | Unigene023645 |  |
| Contig_57115 | Unigene040659 | GABQ01076905 |
| Contig_57126 | Unigene004845 | GABQ01069965 |
| Contig_5713 | Unigene038895 | GABQ01067035 |
| Contig_57162 | Unigene002016 | GABQ01078144 |
| Contig_57185 | Unigene035000 | GABQ01058472 |
| Contig_57208 | Unigene051967 | GABQ01020311 |
| Contig_57218 | Unigene000397 | GABQ01080894 |
| Contig_57248 | Unigene026148 | GABQ01071563 |
| Contig_57316 | Unigene040419 | GABQ01077544 |
| Contig_57347 | Unigene055662 | GABQ01033170 |
| Contig_57369 | Unigene050487 | GABQ01044583 |
| Contig_57388 | Unigene160820 | GABQ01006668 |
| Contig_57390 | Unigene013519 | GABQ01023980 |
| Contig_57421 | Unigene112176 | GABQ01035657 |
| Contig_57465 | Unigene017258 | GABQ01051004 |
| Contig_5747 | Unigene048631 | GABQ01057289 |
| Contig_57488 | Unigene031397 | GABQ01064423 |
| Contig_5749 | Unigene001879 | GABQ01076674 |
| Contig_57556 | Unigene150444 |  |
| Contig_57603 | Unigene003900 | GABQ01076377 |
| Contig_57613 | Unigene035592 | GABQ01054818 |
| Contig_5765 | Unigene005039 | GABQ01068291 |
| Contig_57659 | Unigene115889 | GABQ01010014 |
| Contig_57664 | Unigene001747 | GABQ01078774 |
| Contig_57710 | Unigene002841 | GABQ01063064 |
| Contig_57716 | Unigene041649 | GABQ01080975 |
| Contig_57717 | Unigene003054 | GABQ01046925 |
| Contig_57765 | Unigene002384 | GABQ01073991 |
| Contig_57791 | Unigene042452 | GABQ01072466 |
| Contig_5780 | Unigene041938 | GABQ01073840 |
| Contig_57821 | Unigene047031 | GABQ01062578 |
| Contig_57838 | Unigene039665 | GABQ01080226 |
| Contig_57868 | Unigene046821 | GABQ01063147 |
| Contig_57875 | Unigene009366 | GABQ01037319 |
| Contig_57884 | Unigene081600 | GABQ01001377 |
| Contig_57907 | Unigene048370 | GABQ01058360 |
| Contig_57928 | Unigene048167 | GABQ01059072 |
| Contig_57933 | Unigene002464 | GABQ01074060 |
| Contig_57947 | Unigene047732 | GABQ01060577 |
| Contig_57957 | Unigene008270 | GABQ01055767 |
| Contig_57997 | Unigene019901 | GABQ01062767 |
| Contig_58009 | Unigene038383 | GABQ01075244 |
| Contig_58012 | Unigene042460 | GABQ01072442 |
| Contig_58038 | Unigene009139 | GABQ01038191 |
| Contig_58063 | Unigene029993 | GABQ01029362 |
| Contig_58173 | Unigene159170 | GABQ01052082 |
| Contig_58187 | Unigene018891 | GABQ01031817 |
| Contig_5824 | Unigene140349 |  |
| Contig_58244 | Unigene002316 | GABQ01073159 |
| Contig_58272 | Unigene155771 |  |
| Contig_58281 | Unigene014363 | GABQ01066415 |
| Contig_5838 | Unigene050638 | GABQ01041803 |
| Contig_58420 | Unigene034634 | GABQ01016053 |
| Contig_58470 | Unigene040475 | GABQ01077353 |
| Contig_58506 | Unigene040234 | GABQ01077910 |
| Contig_5851 | Unigene041038 | GABQ01075957 |
| Contig_58528 | Unigene036049 |  |
| Contig_58548 | Unigene041558 | GABQ01074745 |
| Contig_58595 | Unigene038913 | GABQ01072753 |
| Contig_58693 | Unigene000276 | GABQ01077903 |
| Contig_58721 | Unigene023048 | GABQ01035792 |
| Contig_5879 | Unigene001378 | GABQ01073505 |
| Contig_5886 | Unigene065250 | GABQ01049613 |
| Contig_59016 | Unigene016604 | GABQ01052686 |
| Contig_59180 | Unigene100909 |  |
| Contig_59214 | Unigene037431 | GABQ01077433 |
| Contig_59227 | Unigene027493 | GABQ01071603 |
| Contig_59235 | Unigene023061 | GABQ01070960 |
| Contig_59249 | Unigene042750 | GABQ01071809 |
| Contig_59260 | Unigene002918 | GABQ01060811 |
| Contig_59288 | Unigene033126 | GABQ01076956 |
| Contig_59291 | Unigene010124 | GABQ01006080 |
| Contig_5933 | Unigene111663 | GABQ01071685 |
| Contig_59421 | Unigene042573 | GABQ01072211 |
| Contig_59424 | Unigene049077 | GABQ01053817 |
| Contig_59451 | Unigene041284 | GABQ01075381 |
| Contig_59514 | Unigene119094 | GABQ01022482 |
| Contig_59586 | Unigene000252 | GABQ01080480 |
| Contig_59645 | Unigene015678 | GABQ01063294 |
| Contig_59654 | Unigene041541 | GABQ01074812 |
| Contig_59671 | Unigene041917 | GABQ01073869 |
| Contig_59685 | Unigene019214 | GABQ01035780 |
| Contig_59718 | Unigene042100 | GABQ01073531 |
| Contig_59803 | Unigene042350 | GABQ01072870 |
| Contig_59827 | Unigene037658 | GABQ01072799 |
| Contig_59845 | Unigene008082 | GABQ01062895 |
| Contig_59888 | Unigene001521 | GABQ01079754 |
| Contig_59958 | Unigene002419 | GABQ01071744 |
| Contig_60034 | Unigene002173 | GABQ01074421 |
| Contig_60048 | Unigene002320 | GABQ01072248 |
| Contig_60055 | Unigene003142 |  |
| Contig_60077 | Unigene000350 | GABQ01066373 |
| Contig_60094 | Unigene004415 | GABQ01073356 |
| Contig_60105 | Unigene004533 | GABQ01072338 |
| Contig_60119 | Unigene000960 | GABQ01077299 |
| Contig_60185 | Unigene146429 | GABQ01005415 |
| Contig_60243 | Unigene024538 | GABQ01034552 |
| Contig_60316 | Unigene038913 | GABQ01072753 |
| Contig_60325 | Unigene041938 | GABQ01073840 |
| Contig_60335 | Unigene019653 | GABQ01011848 |
| Contig_60350 | Unigene004386 | GABQ01074172 |
| Contig_60370 | Unigene036067 | GABQ01052603 |
| Contig_60373 | Unigene034859 | GABQ01042567 |
| Contig_60393 | Unigene003731 | GABQ01077546 |
| Contig_60442 | Unigene051694 | GABQ01027192 |
| Contig_60443 | Unigene005077 |  |
| Contig_60466 | Unigene028869 | GABQ01058604 |
| Contig_60480 | Unigene048564 | GABQ01057514 |
| Contig_60494 | Unigene038543 | GABQ01072590 |
| Contig_60509 | Unigene032796 | GABQ01061018 |
| Contig_60519 | Unigene048456 | GABQ01058055 |
| Contig_60524 | Unigene002349 | GABQ01073308 |
| Contig_60526 | Unigene019131 | GABQ01049823 |
| Contig_60529 | Unigene001466 | GABQ01080216 |
| Contig_60567 | Unigene003128 | GABQ01080934 |
| Contig_60570 | Unigene035779 | GABQ01024719 |
| Contig_60581 | Unigene047944 | GABQ01059058 |
| Contig_60588 | Unigene005301 | GABQ01064957 |
| Contig_60600 | Unigene023723 |  |
| Contig_60602 | Unigene024295 | GABQ01047348 |
| Contig_60603 | Unigene052471 | GABQ01003981 |
| Contig_60608 | Unigene047558 | GABQ01061149 |
| Contig_60618 | Unigene044838 | GABQ01067308 |
| Contig_60624 | Unigene151633 | GABQ01006960 |
| Contig_60631 | Unigene002448 | GABQ01075405 |
| Contig_60635 | Unigene040749 | GABQ01076671 |
| Contig_60641 | Unigene015917 | GABQ01034854 |
| Contig_60649 | Unigene015987 | GABQ01020811 |
| Contig_60653 | Unigene008195 | GABQ01030261 |
| Contig_60667 | Unigene023324 | GABQ01036619 |
| Contig_60672 | Unigene047579 | GABQ01061040 |
| Contig_60697 | Unigene031512 | GABQ01058028 |
| Contig_60712 | Unigene052369 | GABQ01009073 |
| Contig_60742 | Unigene005735 | GABQ01040445 |
| Contig_60783 | Unigene021053 | GABQ01056062 |
| Contig_60815 | Unigene009137 | GABQ01026998 |
| Contig_60820 | Unigene005586 | GABQ01055949 |
| Contig_60832 | Unigene006751 | GABQ01017122 |
| Contig_60837 | Unigene002293 | GABQ01072861 |
| Contig_60843 | Unigene006476 | GABQ01016521 |
| Contig_60875 | Unigene037585 | GABQ01077960 |
| Contig_60892 | Unigene051939 | GABQ01021573 |
| Contig_60943 | Unigene015356 | GABQ01048656 |
| Contig_60987 | Unigene014392 | GABQ01013231 |
| Contig_60990 | Unigene010583 | GABQ01025830 |
| Contig_61034 | Unigene001492 | GABQ01080328 |
| Contig_61055 | Unigene072018 | GABQ01014466 |
| Contig_61061 | Unigene047082 | GABQ01062491 |
| Contig_61069 | Unigene030381 | GABQ01025345 |
| Contig_61098 | Unigene004125 | GABQ01075141 |
| Contig_61099 | Unigene045558 | GABQ01065910 |
| Contig_61104 | Unigene036989 | GABQ01070560 |
| Contig_61108 | Unigene044694 | GABQ01067681 |
| Contig_61115 | Unigene021758 | GABQ01030339 |
| Contig_61125 | Unigene003127 | GABQ01080936 |
| Contig_61130 | Unigene005815 | GABQ01046770 |
| Contig_61140 | Unigene044723 | GABQ01067583 |
| Contig_61151 | Unigene052280 | GABQ01012401 |
| Contig_6118 | Unigene006358 | GABQ01070938 |
| Contig_6119 | Unigene008491 | GABQ01050076 |
| Contig_61262 | Unigene052040 | GABQ01019079 |
| Contig_61269 | Unigene043001 | GABQ01071212 |
| Contig_61270 | Unigene010516 | GABQ01048896 |
| Contig_61287 | Unigene044134 | GABQ01068495 |
| Contig_61302 | Unigene026524 | GABQ01040171 |
| Contig_61327 | Unigene039382 | GABQ01036406 |
| Contig_61328 | Unigene003453 | GABQ01079104 |
| Contig_61330 | Unigene040795 | GABQ01076569 |
| Contig_61337 | Unigene020329 | GABQ01021458 |
| Contig_61354 | Unigene002171 | GABQ01074019 |
| Contig_61367 | Unigene042083 | GABQ01073244 |
| Contig_61415 | Unigene011244 | GABQ01016547 |
| Contig_61430 | Unigene002142 | GABQ01074420 |
| Contig_61441 | Unigene052401 | GABQ01008040 |
| Contig_61442 | Unigene040482 | GABQ01077364 |
| Contig_61459 | Unigene037967 | GABQ01077040 |
| Contig_61470 | Unigene003600 | GABQ01078372 |
| Contig_6150 | Unigene067934 |  |
| Contig_61505 | Unigene011099 | GABQ01012288 |
| Contig_61521 | Unigene043600 | GABQ01069865 |
| Contig_61524 | Unigene043485 | GABQ01070130 |
| Contig_61587 | Unigene051854 | GABQ01023498 |
| Contig_61629 | Unigene029029 | GABQ01043545 |
| Contig_61639 | Unigene020653 | GABQ01003382 |
| Contig_61647 | Unigene000140 | GABQ01071937 |
| Contig_61703 | Unigene000830 | GABQ01072353 |
| Contig_61708 | Unigene043639 | GABQ01069830 |
| Contig_61750 | Unigene051527 | GABQ01030970 |
| Contig_61753 | Unigene004754 | GABQ01070348 |
| Contig_61763 | Unigene046424 | GABQ01064042 |
| Contig_61776 | Unigene020823 | GABQ01059913 |
| Contig_61799 | Unigene039661 | GABQ01080241 |
| Contig_61809 | Unigene003727 | GABQ01077593 |
| Contig_6181 | Unigene122396 |  |
| Contig_61818 | Unigene009478 | GABQ01034500 |
| Contig_61826 | Unigene041921 | GABQ01073946 |
| Contig_61828 | Unigene050905 | GABQ01039884 |
| Contig_61851 | Unigene006897 | GABQ01001262 |
| Contig_61868 | Unigene008455 | GABQ01028675 |
| Contig_61882 | Unigene039897 | GABQ01079251 |
| Contig_61883 | Unigene043229 | GABQ01070738 |
| Contig_61907 | Unigene003089 | GABQ01042348 |
| Contig_61944 | Unigene000596 | GABQ01076790 |
| Contig_61966 | Unigene006446 |  |
| Contig_61970 | Unigene049517 | GABQ01052385 |
| Contig_61971 | Unigene038771 | GABQ01066190 |
| Contig_62081 | Unigene042743 | GABQ01071829 |
| Contig_62087 | Unigene028132 | GABQ01045636 |
| Contig_62131 | Unigene032073 | GABQ01026557 |
| Contig_62136 | Unigene052074 | GABQ01017886 |
| Contig_62149 | Unigene046335 |  |
| Contig_62151 | Unigene052512 | GABQ01002635 |
| Contig_62165 | Unigene018870 | GABQ01036055 |
| Contig_62183 | Unigene019568 | GABQ01061120 |
| Contig_62193 | Unigene020655 | GABQ01022752 |
| Contig_6220 | Unigene076250 | GABQ01064796 |
| Contig_62202 | Unigene035557 | GABQ01034294 |
| Contig_62212 | Unigene047311 | GABQ01061919 |
| Contig_62242 | Unigene029993 | GABQ01029362 |
| Contig_62268 | Unigene000681 | GABQ01079071 |
| Contig_6230 | Unigene000018 | GABQ01073380 |
| Contig_62303 | Unigene046845 | GABQ01062693 |
| Contig_62377 | Unigene051528 | GABQ01030971 |
| Contig_62412 | Unigene007357 | GABQ01026425 |
| Contig_62414 | Unigene026047 | GABQ01077449 |
| Contig_62423 | Unigene003804 | GABQ01077063 |
| Contig_6246 | Unigene004698 | GABQ01071199 |
| Contig_62465 | Unigene037834 | GABQ01079320 |
| Contig_62477 | Unigene035295 | GABQ01060314 |
| Contig_62481 | Unigene010053 | GABQ01025218 |
| Contig_62538 | Unigene003178 | GABQ01080647 |
| Contig_62553 | Unigene005687 | GABQ01077127 |
| Contig_62572 | Unigene050849 | GABQ01041485 |
| Contig_62582 | Unigene052451 | GABQ01005186 |
| Contig_62591 | Unigene023282 | GABQ01022764 |
| Contig_62593 | Unigene039160 | GABQ01046510 |
| Contig_62604 | Unigene030688 | GABQ01054607 |
| Contig_62615 | Unigene003655 | GABQ01078017 |
| Contig_62637 | Unigene019512 | GABQ01064868 |
| Contig_62652 | Unigene003142 |  |
| Contig_62666 | Unigene050388 | GABQ01045671 |
| Contig_62680 | Unigene041101 | GABQ01075823 |
| Contig_62740 | Unigene027967 | GABQ01051497 |
| Contig_62741 | Unigene051317 | GABQ01034951 |
| Contig_62769 | Unigene047698 | GABQ01060631 |
| Contig_62780 | Unigene047658 | GABQ01060679 |
| Contig_62782 | Unigene000198 |  |
| Contig_62787 | Unigene042825 | GABQ01071610 |
| Contig_6279 | Unigene002478 | GABQ01071431 |
| Contig_62805 | Unigene031998 | GABQ01055807 |
| Contig_62856 | Unigene040206 | GABQ01078149 |
| Contig_62871 | Unigene035993 | GABQ01038265 |
| Contig_62883 | Unigene162026 |  |
| Contig_62887 | Unigene001774 | GABQ01080178 |
| Contig_62899 | Unigene039520 | GABQ01017300 |
| Contig_62908 | Unigene045669 | GABQ01065632 |
| Contig_62959 | Unigene003569 | GABQ01078553 |
| Contig_62981 | Unigene050120 | GABQ01048054 |
| Contig_62983 | Unigene007293 | GABQ01024555 |
| Contig_62996 | Unigene009154 | GABQ01014799 |
| Contig_63030 | Unigene051573 | GABQ01029932 |
| Contig_63034 | Unigene041156 | GABQ01075651 |
| Contig_63075 | Unigene002914 | GABQ01061643 |
| Contig_63084 | Unigene031728 |  |
| Contig_63175 | Unigene032765 | GABQ01002342 |
| Contig_63181 | Unigene039917 | GABQ01079173 |
| Contig_63243 | Unigene002741 | GABQ01066715 |
| Contig_63256 | Unigene007514 | GABQ01025810 |
| Contig_63259 | Unigene008316 | GABQ01060591 |
| Contig_63260 | Unigene051609 | GABQ01029416 |
| Contig_63271 | Unigene021787 | GABQ01045248 |
| Contig_63290 | Unigene036529 | GABQ01049749 |
| Contig_63294 | Unigene051974 | GABQ01020316 |
| Contig_63301 | Unigene005862 | GABQ01062758 |
| Contig_63305 | Unigene049167 | GABQ01054729 |
| Contig_63318 | Unigene045253 | GABQ01066578 |
| Contig_63338 | Unigene050134 | GABQ01047923 |
| Contig_63342 | Unigene065287 | GABQ01007735 |
| Contig_63399 | Unigene000269 | GABQ01080839 |
| Contig_63401 | Unigene001223 | GABQ01079085 |
| Contig_63406 | Unigene004312 | GABQ01073992 |
| Contig_63409 | Unigene001969 | GABQ01076116 |
| Contig_63411 | Unigene047662 | GABQ01060446 |
| Contig_63425 | Unigene002439 | GABQ01073631 |
| Contig_63437 | Unigene035843 | GABQ01062139 |
| Contig_63458 | Unigene013574 | GABQ01071096 |
| Contig_63473 | Unigene025512 | GABQ01043975 |
| Contig_63497 | Unigene048547 | GABQ01057436 |
| Contig_63530 | Unigene052493 | GABQ01003710 |
| Contig_63537 | Unigene024206 | GABQ01045093 |
| Contig_63582 | Unigene027309 | GABQ01033500 |
| Contig_63616 | Unigene015230 | GABQ01013234 |
| Contig_6363 | Unigene118885 | GABQ01045837 |
| Contig_63677 | Unigene043459 | GABQ01070211 |
| Contig_63698 | Unigene048306 | GABQ01058191 |
| Contig_63744 | Unigene049476 | GABQ01052848 |
| Contig_63753 | Unigene005558 | GABQ01070895 |
| Contig_63767 | Unigene011837 | GABQ01020158 |
| Contig_6377 | Unigene029183 | GABQ01028267 |
| Contig_63793 | Unigene007408 | GABQ01009363 |
| Contig_63805 | Unigene044954 | GABQ01067114 |
| Contig_63828 | Unigene048679 | GABQ01057142 |
| Contig_63842 | Unigene038327 | GABQ01074742 |
| Contig_63870 | Unigene006022 | GABQ01039587 |
| Contig_63876 | Unigene005836 | GABQ01016517 |
| Contig_63900 | Unigene001236 | GABQ01078976 |
| Contig_63912 | Unigene009892 |  |
| Contig_6392 | Unigene079734 | GABQ01040225 |
| Contig_63939 | Unigene044850 | GABQ01066845 |
| Contig_63941 | Unigene044472 | GABQ01068119 |
| Contig_63961 | Unigene045971 | GABQ01065068 |
| Contig_63971 | Unigene047760 | GABQ01059873 |
| Contig_63999 | Unigene048529 | GABQ01057508 |
| Contig_64029 | Unigene050562 | GABQ01044005 |
| Contig_64039 | Unigene037343 | GABQ01072040 |
| Contig_64046 | Unigene043118 | GABQ01070933 |
| Contig_64073 | Unigene042368 | GABQ01072695 |
| Contig_64081 | Unigene048323 | GABQ01058572 |
| Contig_64097 | Unigene025648 | GABQ01042093 |
| Contig_64130 | Unigene071881 | GABQ01031511 |
| Contig_64136 | Unigene052152 | GABQ01016082 |
| Contig_64137 | Unigene003136 | GABQ01080912 |
| Contig_64199 | Unigene046365 | GABQ01064114 |
| Contig_6422 | Unigene017664 | GABQ01039226 |
| Contig_64233 | Unigene041481 | GABQ01074883 |
| Contig_64322 | Unigene009544 | GABQ01015899 |
| Contig_64350 | Unigene067638 | GABQ01030496 |
| Contig_64372 | Unigene040206 | GABQ01078149 |
| Contig_64381 | Unigene025176 | GABQ01063379 |
| Contig_64397 | Unigene010612 |  |
| Contig_64415 | Unigene039814 | GABQ01079533 |
| Contig_64419 | Unigene092189 | GABQ01038539 |
| Contig_64421 | Unigene101485 | GABQ01030592 |
| Contig_64427 | Unigene008267 | GABQ01056473 |
| Contig_6445 | Unigene038515 | GABQ01072294 |
| Contig_64470 | Unigene047457 | GABQ01060970 |
| Contig_64471 | Unigene037031 | GABQ01076314 |
| Contig_64506 | Unigene047940 | GABQ01059743 |
| Contig_64522 | Unigene002890 | GABQ01066586 |
| Contig_64529 | Unigene001725 | GABQ01078337 |
| Contig_64542 | Unigene049447 | GABQ01053071 |
| Contig_6457 | Unigene123225 | GABQ01071742 |
| Contig_64571 | Unigene001398 |  |
| Contig_64582 | Unigene002508 | GABQ01069926 |
| Contig_64587 | Unigene025127 |  |
| Contig_64614 | Unigene046244 | GABQ01064505 |
| Contig_64677 | Unigene049171 | GABQ01054731 |
| Contig_64688 | Unigene003453 | GABQ01079104 |
| Contig_64699 | Unigene042928 | GABQ01071405 |
| Contig_64708 | Unigene041082 | GABQ01075850 |
| Contig_64745 | Unigene042957 | GABQ01071301 |
| Contig_64752 | Unigene041177 | GABQ01075630 |
| Contig_64773 | Unigene003738 | GABQ01077516 |
| Contig_64787 | Unigene003411 | GABQ01079367 |
| Contig_6480 | Unigene073826 | GABQ01064253 |
| Contig_64827 | Unigene131265 |  |
| Contig_64846 | Unigene000598 | GABQ01076553 |
| Contig_64865 | Unigene001503 | GABQ01079988 |
| Contig_64891 | Unigene128118 | GABQ01023786 |
| Contig_64907 | Unigene001068 | GABQ01077080 |
| Contig_64932 | Unigene002042 | GABQ01076074 |
| Contig_64969 | Unigene001132 | GABQ01080173 |
| Contig_64984 | Unigene037412 | GABQ01080222 |
| Contig_65002 | Unigene022461 | GABQ01063036 |
| Contig_65037 | Unigene010837 | GABQ01074918 |
| Contig_65082 | Unigene016890 |  |
| Contig_65092 | Unigene026381 | GABQ01019019 |
| Contig_65101 | Unigene068736 | GABQ01007740 |
| Contig_65129 | Unigene149396 |  |
| Contig_65145 | Unigene041571 | GABQ01074712 |
| Contig_65146 | Unigene047648 | GABQ01060730 |
| Contig_65156 | Unigene089304 |  |
| Contig_65205 | Unigene052559 |  |
| Contig_65223 | Unigene019461 | GABQ01051238 |
| Contig_65374 | Unigene017846 | GABQ01066264 |
| Contig_65382 | Unigene043000 | GABQ01071211 |
| Contig_65405 | Unigene042706 | GABQ01071930 |
| Contig_6541 | Unigene048127 | GABQ01059210 |
| Contig_65427 | Unigene007198 | GABQ01047447 |
| Contig_6544 | Unigene114448 | GABQ01048994 |
| Contig_65466 | Unigene035422 | GABQ01053303 |
| Contig_65484 | Unigene051828 | GABQ01024144 |
| Contig_65486 | Unigene048142 | GABQ01059214 |
| Contig_65532 | Unigene041630 | GABQ01074583 |
| Contig_65533 | Unigene052302 | GABQ01011913 |
| Contig_65542 | Unigene001240 | GABQ01078210 |
| Contig_65565 | Unigene041576 | GABQ01074698 |
| Contig_65574 | Unigene040881 | GABQ01076316 |
| Contig_65578 | Unigene048399 | GABQ01058276 |
| Contig_65599 | Unigene001174 | GABQ01080801 |
| Contig_65605 | Unigene050602 | GABQ01043874 |
| Contig_65630 | Unigene161054 | GABQ01028651 |
| Contig_65643 | Unigene050268 | GABQ01046700 |
| Contig_65657 | Unigene003131 | GABQ01080929 |
| Contig_65699 | Unigene010246 | GABQ01042500 |
| Contig_65719 | Unigene040509 | GABQ01077137 |
| Contig_65721 | Unigene009679 | GABQ01014269 |
| Contig_65723 | Unigene010753 | GABQ01004542 |
| Contig_65724 | Unigene008718 | GABQ01018912 |
| Contig_65731 | Unigene044805 | GABQ01067457 |
| Contig_65733 | Unigene025807 | GABQ01001294 |
| Contig_65743 | Unigene039901 | GABQ01079242 |
| Contig_65801 | Unigene049208 | GABQ01053931 |
| Contig_65807 | Unigene025171 | GABQ01060039 |
| Contig_65814 | Unigene000978 | GABQ01078622 |
| Contig_65827 | Unigene048912 | GABQ01056111 |
| Contig_65842 | Unigene032865 | GABQ01022825 |
| Contig_65847 | Unigene042928 | GABQ01071405 |
| Contig_65849 | Unigene007098 |  |
| Contig_65855 | Unigene112222 |  |
| Contig_65856 | Unigene149244 |  |
| Contig_65879 | Unigene129690 |  |
| Contig_65885 | Unigene006959 | GABQ01053668 |
| Contig_65898 | Unigene048397 | GABQ01058274 |
| Contig_65900 | Unigene002071 | GABQ01075327 |
| Contig_65910 | Unigene048775 | GABQ01056000 |
| Contig_65947 | Unigene001968 | GABQ01076246 |
| Contig_65954 | Unigene002071 | GABQ01075327 |
| Contig_65961 | Unigene024072 | GABQ01041770 |
| Contig_65966 | Unigene003849 | GABQ01076707 |
| Contig_66021 | Unigene040421 | GABQ01077527 |
| Contig_6603 | Unigene020730 | GABQ01024026 |
| Contig_66039 | Unigene051535 | GABQ01029921 |
| Contig_66067 | Unigene052346 | GABQ01010228 |
| Contig_66072 | Unigene099149 | GABQ01031576 |
| Contig_66088 | Unigene001272 | GABQ01076516 |
| Contig_66092 | Unigene010844 | GABQ01025833 |
| Contig_66109 | Unigene023500 | GABQ01053900 |
| Contig_66112 | Unigene014744 | GABQ01071783 |
| Contig_66114 | Unigene013124 | GABQ01015922 |
| Contig_66126 | Unigene044550 | GABQ01067882 |
| Contig_66152 | Unigene044199 | GABQ01068566 |
| Contig_66170 | Unigene026964 | GABQ01046188 |
| Contig_66172 | Unigene050552 | GABQ01044454 |
| Contig_66216 | Unigene048978 | GABQ01055833 |
| Contig_66225 | Unigene003153 | GABQ01080798 |
| Contig_66226 | Unigene051474 | GABQ01031908 |
| Contig_66232 | Unigene026810 | GABQ01056946 |
| Contig_66239 | Unigene005223 | GABQ01065984 |
| Contig_66268 | Unigene046406 | GABQ01063671 |
| Contig_66281 | Unigene036754 | GABQ01080581 |
| Contig_66333 | Unigene008044 | GABQ01011347 |
| Contig_66381 | Unigene038864 | GABQ01070613 |
| Contig_66385 | Unigene005608 | GABQ01054578 |
| Contig_66387 | Unigene000645 | GABQ01080432 |
| Contig_66410 | Unigene002971 | GABQ01056731 |
| Contig_66423 | Unigene041123 | GABQ01075694 |
| Contig_66424 | Unigene013437 | GABQ01039613 |
| Contig_66446 | Unigene037188 | GABQ01068734 |
| Contig_66470 | Unigene004948 | GABQ01069303 |
| Contig_6648 | Unigene034937 |  |
| Contig_66497 | Unigene003935 | GABQ01076311 |
| Contig_66510 | Unigene014053 |  |
| Contig_66517 | Unigene020613 | GABQ01051128 |
| Contig_66537 | Unigene047861 | GABQ01060058 |
| Contig_66538 | Unigene136832 | GABQ01055158 |
| Contig_66574 | Unigene008214 | GABQ01066815 |
| Contig_66579 | Unigene008876 | GABQ01009366 |
| Contig_66624 | Unigene156742 | GABQ01041106 |
| Contig_66625 | Unigene039487 | GABQ01043999 |
| Contig_66638 | Unigene000895 | GABQ01080922 |
| Contig_66658 | Unigene010508 | GABQ01057467 |
| Contig_66684 | Unigene041179 | GABQ01075598 |
| Contig_66698 | Unigene159473 |  |
| Contig_66708 | Unigene005881 | GABQ01000002 |
| Contig_66713 | Unigene049386 | GABQ01052256 |
| Contig_66759 | Unigene048749 | GABQ01056796 |
| Contig_66765 | Unigene036885 | GABQ01080903 |
| Contig_66767 | Unigene002802 | GABQ01065344 |
| Contig_66782 | Unigene043043 | GABQ01071134 |
| Contig_66790 | Unigene017963 | GABQ01009798 |
| Contig_6680 | Unigene085539 | GABQ01073086 |
| Contig_66816 | Unigene024099 | GABQ01049060 |
| Contig_66826 | Unigene047730 | GABQ01060323 |
| Contig_66830 | Unigene019157 | GABQ01012325 |
| Contig_66855 | Unigene040323 | GABQ01077787 |
| Contig_66872 | Unigene029963 | GABQ01050259 |
| Contig_66880 | Unigene003285 | GABQ01079993 |
| Contig_66895 | Unigene046824 | GABQ01063192 |
| Contig_66903 | Unigene043874 | GABQ01069224 |
| Contig_66905 | Unigene016588 | GABQ01060216 |
| Contig_6691 | Unigene134038 | GABQ01006008 |
| Contig_6692 | Unigene004548 | GABQ01072123 |
| Contig_66942 | Unigene052590 |  |
| Contig_66993 | Unigene044757 | GABQ01067556 |
| Contig_66994 | Unigene024645 | GABQ01028239 |
| Contig_66998 | Unigene040555 | GABQ01077166 |
| Contig_6700 | Unigene019899 | GABQ01074786 |
| Contig_67001 | Unigene040892 | GABQ01076305 |
| Contig_67015 | Unigene040386 | GABQ01077626 |
| Contig_67026 | Unigene000166 | GABQ01080901 |
| Contig_67027 | Unigene005917 | GABQ01008653 |
| Contig_67037 | Unigene003131 | GABQ01080929 |
| Contig_67075 | Unigene012644 | GABQ01015338 |
| Contig_67094 | Unigene000200 | GABQ01078249 |
| Contig_67095 | Unigene050450 | GABQ01045140 |
| Contig_67100 | Unigene049090 | GABQ01055140 |
| Contig_67101 | Unigene043797 | GABQ01069373 |
| Contig_67108 | Unigene136607 | GABQ01026873 |
| Contig_67114 | Unigene050360 | GABQ01045949 |
| Contig_67137 | Unigene017572 |  |
| Contig_67147 | Unigene031733 |  |
| Contig_67221 | Unigene017188 | GABQ01056835 |
| Contig_67245 | Unigene032018 | GABQ01066076 |
| Contig_67257 | Unigene001696 | GABQ01080401 |
| Contig_67264 | Unigene008549 | GABQ01044373 |
| Contig_67274 | Unigene097889 |  |
| Contig_67289 | Unigene050864 | GABQ01040873 |
| Contig_67300 | Unigene038129 | GABQ01072978 |
| Contig_67349 | Unigene050793 | GABQ01042149 |
| Contig_67350 | Unigene001128 | GABQ01066374 |
| Contig_67358 | Unigene139003 | GABQ01052419 |
| Contig_67408 | Unigene109668 | GABQ01004109 |
| Contig_67433 | Unigene043550 | GABQ01070008 |
| Contig_67489 | Unigene047606 | GABQ01060320 |
| Contig_67503 | Unigene037862 | GABQ01080392 |
| Contig_67514 | Unigene003638 | GABQ01078145 |
| Contig_67519 | Unigene161535 | GABQ01050608 |
| Contig_67522 | Unigene044303 | GABQ01068132 |
| Contig_67523 | Unigene088677 | GABQ01054459 |
| Contig_67572 | Unigene017867 | GABQ01080517 |
| Contig_67580 | Unigene010421 | GABQ01059707 |
| Contig_67639 | Unigene006695 | GABQ01034804 |
| Contig_67649 | Unigene041726 | GABQ01074309 |
| Contig_67654 | Unigene052400 | GABQ01008039 |
| Contig_67665 | Unigene044347 | GABQ01068053 |
| Contig_67679 | Unigene005677 | GABQ01073414 |
| Contig_67700 | Unigene050541 | GABQ01044450 |
| Contig_67737 | Unigene119622 |  |
| Contig_67748 | Unigene051766 | GABQ01025397 |
| Contig_67751 | Unigene008638 | GABQ01031302 |
| Contig_67763 | Unigene000038 | GABQ01074383 |
| Contig_67766 | Unigene042757 | GABQ01071811 |
| Contig_67780 | Unigene000572 | GABQ01078852 |
| Contig_67782 | Unigene001399 | GABQ01080914 |
| Contig_67839 | Unigene068604 |  |
| Contig_67845 | Unigene003453 | GABQ01079104 |
| Contig_67861 | Unigene020629 | GABQ01030330 |
| Contig_6788 | Unigene039759 | GABQ01079761 |
| Contig_67884 | Unigene004545 | GABQ01072252 |
| Contig_67959 | Unigene138950 | GABQ01023823 |
| Contig_68008 | Unigene001328 | GABQ01080941 |
| Contig_68012 | Unigene041490 | GABQ01074687 |
| Contig_68040 | Unigene000043 | GABQ01075921 |
| Contig_68053 | Unigene003163 | GABQ01080749 |
| Contig_68073 | Unigene028048 |  |
| Contig_68091 | Unigene030187 | GABQ01022803 |
| Contig_68102 | Unigene037946 | GABQ01078953 |
| Contig_68110 | Unigene003428 | GABQ01079301 |
| Contig_68149 | Unigene000133 | GABQ01080485 |
| Contig_68157 | Unigene128846 | GABQ01029677 |
| Contig_68220 | Unigene044962 | GABQ01067166 |
| Contig_68223 | Unigene037682 | GABQ01069692 |
| Contig_68251 | Unigene044962 | GABQ01067166 |
| Contig_68258 | Unigene084369 | GABQ01075799 |
| Contig_68288 | Unigene118349 | GABQ01070190 |
| Contig_68324 | Unigene023251 | GABQ01054249 |
| Contig_6850 | Unigene058168 | GABQ01073454 |
| Contig_6906 | Unigene063800 | GABQ01071618 |
| Contig_6947 | Unigene112153 | GABQ01055751 |
| Contig_7003 | Unigene087679 | GABQ01047274 |
| Contig_7014 | Unigene107030 | GABQ01054645 |
| Contig_7020 | Unigene027475 | GABQ01057803 |
| Contig_7120 | Unigene148296 | GABQ01063320 |
| Contig_7142 | Unigene059855 | GABQ01072411 |
| Contig_7226 | Unigene044176 | GABQ01068372 |
| Contig_728 | Unigene027264 | GABQ01071337 |
| Contig_7343 | Unigene005120 | GABQ01067332 |
| Contig_7449 | Unigene044421 | GABQ01068220 |
| Contig_7498 | Unigene047711 | GABQ01060571 |
| Contig_7517 | Unigene134114 | GABQ01009680 |
| Contig_7532 | Unigene158771 | GABQ01051215 |
| Contig_7540 | Unigene068864 | GABQ01073544 |
| Contig_7561 | Unigene145530 | GABQ01050337 |
| Contig_7582 | Unigene065579 | GABQ01056116 |
| Contig_7656 | Unigene048419 | GABQ01058203 |
| Contig_7712 | Unigene090010 | GABQ01021088 |
| Contig_777 | Unigene004941 | GABQ01069086 |
| Contig_7799 | Unigene086895 | GABQ01019200 |
| Contig_7813 | Unigene112631 | GABQ01012580 |
| Contig_7835 | Unigene048374 | GABQ01058266 |
| Contig_7837 | Unigene002478 | GABQ01071431 |
| Contig_7899 | Unigene080108 | GABQ01013440 |
| Contig_7904 | Unigene117138 | GABQ01070030 |
| Contig_7913 | Unigene130157 | GABQ01010445 |
| Contig_7924 | Unigene096662 | GABQ01021759 |
| Contig_7942 | Unigene048166 | GABQ01059071 |
| Contig_7983 | Unigene012589 | GABQ01075009 |
| Contig_7984 | Unigene088444 | GABQ01032424 |
| Contig_8066 | Unigene011421 | GABQ01042224 |
| Contig_8068 | Unigene068074 | GABQ01070662 |
| Contig_808 | Unigene002271 | GABQ01076061 |
| Contig_8105 | Unigene037734 | GABQ01076573 |
| Contig_8116 | Unigene043370 | GABQ01070274 |
| Contig_8131 | Unigene014744 | GABQ01071783 |
| Contig_8158 | Unigene038948 | GABQ01069094 |
| Contig_8198 | Unigene019860 | GABQ01055971 |
| Contig_8199 | Unigene038094 | GABQ01075476 |
| Contig_8326 | Unigene046976 | GABQ01062794 |
| Contig_8358 | Unigene054237 | GABQ01022247 |
| Contig_8363 | Unigene005479 | GABQ01060995 |
| Contig_84 | Unigene040611 | GABQ01077045 |
| Contig_8442 | Unigene158783 | GABQ01042485 |
| Contig_8466 | Unigene080819 | GABQ01064171 |
| Contig_8491 | Unigene000272 | GABQ01079297 |
| Contig_8515 | Unigene004032 | GABQ01075723 |
| Contig_8703 | Unigene003652 | GABQ01077996 |
| Contig_8721 | Unigene040342 | GABQ01077720 |
| Contig_8723 | Unigene000280 | GABQ01070344 |
| Contig_8754 | Unigene154666 | GABQ01072220 |
| Contig_8768 | Unigene022571 | GABQ01005494 |
| Contig_8812 | Unigene089014 | GABQ01056293 |
| Contig_8814 | Unigene047158 | GABQ01062315 |
| Contig_8847 | Unigene068288 | GABQ01072245 |
| Contig_8883 | Unigene081901 | GABQ01073141 |
| Contig_892 | Unigene002123 | GABQ01074634 |
| Contig_8956 | Unigene026320 | GABQ01046961 |
| Contig_8975 | Unigene140349 |  |
| Contig_902 | Unigene046850 | GABQ01063108 |
| Contig_905 | Unigene004105 | GABQ01075293 |
| Contig_9081 | Unigene041129 | GABQ01075745 |
| Contig_9240 | Unigene043788 | GABQ01069465 |
| Contig_9252 | Unigene048255 | GABQ01058790 |
| Contig_9387 | Unigene092952 | GABQ01072952 |
| Contig_9450 | Unigene105678 | GABQ01071553 |
| Contig_9472 | Unigene160564 | GABQ01072721 |
| Contig_9500 | Unigene038581 | GABQ01070534 |
| Contig_951 | Unigene018250 | GABQ01065809 |
| Contig_9548 | Unigene021913 | GABQ01071202 |
| Contig_9578 | Unigene006454 | GABQ01077025 |
| Contig_9612 | Unigene008618 | GABQ01070216 |
| Contig_9629 | Unigene063723 | GABQ01055246 |
| Contig_9654 | Unigene070530 | GABQ01068067 |
| Contig_9671 | Unigene041030 | GABQ01075944 |
| Contig_9713 | Unigene042525 | GABQ01072296 |
| Contig_9717 | Unigene050887 | GABQ01040542 |
| Contig_9751 | Unigene064438 |  |
| Contig_9800 | Unigene072669 |  |
| Contig_9811 | Unigene031264 | GABQ01069949 |
| Contig_9881 | Unigene011336 | GABQ01070509 |
| Contig_9896 | Unigene038020 | GABQ01075615 |
| Contig_9936 | Unigene027953 | GABQ01075002 |
| Contig_9948 | Unigene015787 | GABQ01009789 |
| Contig_9958 | Unigene040240 | GABQ01078030 |
| Contig_997 | Unigene040369 | GABQ01077676 |
| Contig_9975 | Unigene041580 | GABQ01074664 |
| Contig_9978 | Unigene034588 | GABQ01065786 |

**Table S5**. Expression values for the RT-*q*PCRs in the *Arabidopsis* mutants. *svp*-41 has a 2bp deletion with frameshift change. *dwf*4-101 has a single nucleotide substitution resulting in a residue substitution in a highly conserved domain.

| **Gene** | **Mutant** | **Tissue** | **Primer** | **Expression Ratio Mutant/WT** | **Primer** | **Expression Ratio Mutant/WT** |
| --- | --- | --- | --- | --- | --- | --- |
| CDC45 | SALK_128351C | 2 leaves stage | L7 | 3.009 |  |  |
| SCL30 | SALK_139541C | 2 leaves stage | C2 | 0.041 | C15 | 2.610 |
| EDL2 | SALK_018189 | 2 leaves stage | AB1 | 0 | AB14 | 0.149 |
|  | SALK_097615C |  |  | 0.008 |  | 0.011 |
| SVP | SALK_072930C | 2 leaves stage | EF4 | 0.014 | EF17 | 0.377 |
|  | *svp-41* |  |  | 13.379 |  | 8.679 |
| TPR1 | SALK_065650C | 2 leaves stage | W12 | 1.759 |  |  |
